# Molecular and behavioral studies reveal differences in olfaction between winter and summer morphs of *Drosophila suzukii*

**DOI:** 10.7717/peerj.13825

**Published:** 2022-09-16

**Authors:** Timothy W. Schwanitz, James J. Polashock, Dara G. Stockton, Cesar Rodriguez-Saona, Diego Sotomayor, Greg Loeb, Chloe Hawkings

**Affiliations:** 1 Entomology, Rutgers, The State University of New Jersey, New Brunswick, NJ, United States of America; 2 Genetic Improvement of Fruits and Vegetables Laboratory, USDA-ARS, Chatsworth, NJ, United States of America; 3 Entomology, Cornell University, Geneva, NY, United States of America; 4 Agro-Environmental Science Department, University of Puerto Rico, Mayagüez, Puerto Rico, United States of America

**Keywords:** Transcriptomics, Behavior, Olfaction, Entomology

## Abstract

Spotted-wing drosophila, *Drosophila suzukii* (Matsumura), is a major economic pest of several fruit crops in Europe, North and South America, and other parts of the world because it oviposits in ripening thin-skinned fruits. This vinegar fly exhibits two distinct morphotypes: a summer and a winter morph. Although adaptations associated with the winter morph enhance this invasive pest’s capacity to survive in cold climates, winter is still a natural population bottleneck. Since monitoring early spring populations is important for accurate population forecasts, understanding the winter morph’s response to olfactory cues may improve current *D. suzukii* management programs. In this study, a comparative transcriptome analysis was conducted to assess gene expression differences between the female heads of the two *D. suzukii* morphs, which showed significant differences in 738 genes (*p* ≤ 0.0001). Out of twelve genes related to olfaction determined to be differentially expressed in the transcriptome, *i.e.*, those related to location of food sources, chemosensory abilities, and mating behavior, nine genes were upregulated in the winter morph while three were downregulated. Three candidate olfactory-related genes that were most upregulated or downregulated in the winter morph were further validated using RT-qPCR. In addition, behavioral assays were performed at a range of temperatures to confirm a differing behavioral response of the two morphs to food odors. Our behavioral assays showed that, although winter morphs were more active at lower temperatures, the summer morphs were generally more attracted to food odors. This study provides new insights into the molecular and behavioral differences in response to olfactory cues between the two *D. suzukii* morphs that will assist in formulating more effective monitoring and physiological-based control tools.

## Introduction

Spotted-wing drosophila, *Drosophila suzukii* (Matsumura) (Diptera: Drosophilidae), is an invasive pest of thin-skinned fruits such as blueberries, raspberries, and strawberries, and stone fruits such as peaches, cherries, and apricots (Asplen et al., 2015). Females use their characteristic serrated ovipositor to lay eggs in ripening or ripe fruit, unlike many closely related drosophilds that oviposit in rotting fruit. There is low consumer tolerance for infested fruit, as larvae feed internally and render it inedible. Moreover, fruit harvests that are infested with *D. suzukii* larvae may be placed under a zero-tolerance quarantine, which would prevent export to areas outside of the quarantine (Feng et al., 2018). Current management of this invasive pest relies on frequent insecticide applications, *e.g.*, five to eight sprays per season in blueberry fields (Mermer et al., 2020). Because of substantial dependance on insecticides, novel strategies seek to use more environmentally sustainable solutions (Tait et al., 2021). Integrated pest management (IPM) programs for managing *D. suzukii* infestations offer a number of such potential solutions. One key component of successful IPM programs is a shift away from calendar-based sprays; instead, insecticides should only be applied when the pest pressure and risk of infestation warrant spraying (Tait et al., 2021). An effective assessment of infestation risk, however, requires reliable population monitoring methods, and these methods often depend on lures. Furthermore, numerous alternative strategies to chemical control have been proposed and tested to manage this invasive fly (Tait et al., 2021), including several that require effective lures, *e.g.*, attract-and-kill systems (Klick et al., 2019; Bianchi et al., 2020). Yet, the effectiveness of all of these strategies, particularly those based on lures and *D. suzukii* behavior, can be influenced by seasonal changes in the fly’s olfaction.

**Figure 1:**
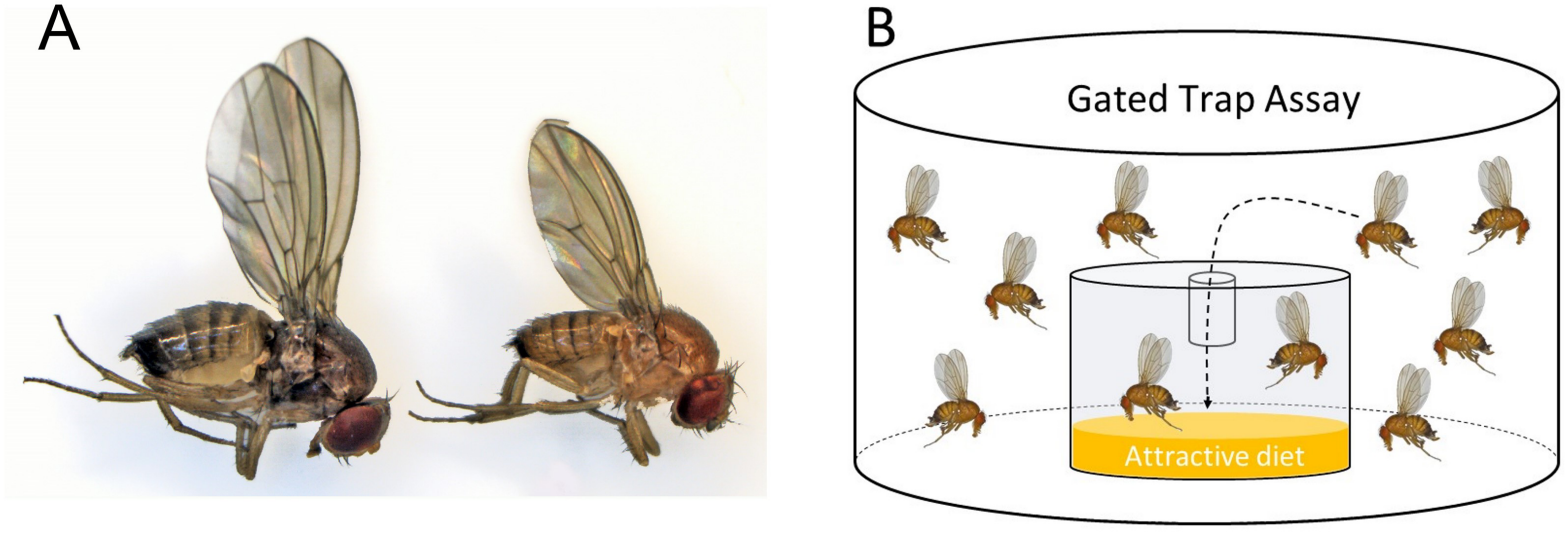
Morphotype illustration. (A) Image of winter morph (left) and summer morph (right) *Drosophila suzukii* flies. The winter morph of this fly is larger, darker, and has longer wings. (B) A gated trap-capture assay was used to measure the behavioral response of adult female *Drosophila suzukii* at five different temperatures ranging from 0–25 °C for the summer morph and winter morph phenotypes.

*Drosophila suzukii* flies overwinter as adults, usually in a distinct seasonal form referred to as the winter morphotype (Stephens et al., 2015). Winter morph flies are morphologically and physiologically distinct from summer morph flies—they are larger and display darker cuticular pigmentation (Fig. 1A). They develop from larvae exposed to shorter photo-periods and colder temperatures (Shearer et al., 2016; Tran, Hutchison & Asplen, 2020; Stockton et al., 2020). The number of adult *D. suzukii* flies that successfully overwinter determines the fly population size in early spring and summer (Briem et al., 2018). Thus, assessing winter morph population size is important for developing IPM programs, as the overwintering flies can infest the season’s first commercial crops (Panel et al., 2020). Wintertime is a natural population bottleneck when most of the surviving *D. suzukii* adults appear to be clustered in hedges and woodlands, where they likely encounter a different odor space and therefore respond differently to olfactory cues (Pelton et al., 2016); hence, winter may be an ideal time to implement control measures to reduce spring and early summer populations (Briem et al., 2018; Leach et al., 2019).

In northern temperate regions, lure-based monitoring traps deployed during the winter fail to attract many flies—despite experimental evidence that suggests these flies should be active during warm winter days, and despite studies that have trapped these flies in below-freezing weather (Stockton, Wallingford & Loeb, 2018; Stockton et al., 2019). Lures that effectively attract summer morph flies may not be as attractive to winter morph flies. For instance, a study using electroantennogram and behavioral assays found differences in the attractiveness of various volatiles to adult winter and summer *D. suzukii* morphs (Kirkpatrick et al., 2018). Winter morph flies may feed on and seek out non-fruit resources, thus shifting their olfactory preferences (Stockton, Brown & Loeb, 2019). However, in another study that investigated this possibility, the authors found no significant difference in the attractiveness of certain baits to adult winter and summer *D. suzukii* morphs (Wong et al., 2018). Therefore, a better understanding of the molecular and behavioral differences in the response of winter and summer *D. suzukii* morphs to olfactory cues involved in food and mate location will help to develop more effective monitoring tools. In fact, transcriptome studies have already shown that cold acclimation in *D. suzukii* is associated with major changes in gene expression, as 2,908 genes were found to be differentially expressed in flies acclimated to 10 ° C relative to flies that had been kept at 25 °C (Enriquez & Colinet, 2019).

The first goal of this paper was to determine differential expression of genes between winter and summer *D. suzukii* morphs and to evaluate candidate genes that could play a role in the regulation of olfactory cues and potentially be the cause for lower efficacy of monitoring traps during the winter. Here, we performed a transcriptome analysis in the heads of winter and summer *D. suzukii* morphs to identify genes related to olfaction. Heads were used because olfactory integration occurs in the brain, and the antennae are the primary olfactory organs. As our second goal, we conducted behavioral assays to determine if winter and summer *D. suzukii* morphs do indeed respond differently to food odors. This study aims to provide the molecular basis for the differential behavioral response of winter and summer *D. suzukii* morphs to odors and lure-based monitoring traps.

## Materials and Methods

### Data source for transcriptome analysis

For the transcriptome analysis, publicly available raw data files were downloaded from the sequence read archives from the European Nucleotide Archive (ENA) with the accession number PRJNA294845, on the ENA website (https://www.ebi.ac.uk/ena). These reads were uploaded by Shearer et al. (2016). The libraries were prepared with the Illumina TruSeq RNA Sample Preparation kit (Illumina, Inc.; San Diego, CA, USA), and samples were sequenced on the Illumina HiSeq 2000 (Illumina, Inc.; San Diego, CA, USA) using paired-end 100 bp sequencing (Shearer et al., 2016). We downloaded three RNA read samples of the heads of female winter morph *D. suzukii* flies and three RNA samples of the heads of female summer morph *D. suzukii* flies. According to Shearer et al. (2016), each RNA sample was comprised of 15 individual female flies of either the winter or summer morph from a laboratory-reared colony.

### Transcriptome analysis and gene annotation

Bioinformatic analyses were performed using the Galaxy platform and web interface (Afgan et al., 2018). The RNA-seq reads that passed the quality filters (FASTQC tools) were mapped to the *D. suzukii* genome (Shearer et al., 2016) using HISAT2. Transcripts were assembled using StringTie (using only reference transcripts). Differentially expressed genes were identified and visualized using DESeq2 (Love, Huber & Anders, 2014). The identification of differentially expressed genes was performed with the following criteria: false discovery rate (FDR < 0.01), with a *p* value ≤ 0.0001 and a log2 fold-change value of ≥ 0.5, for upregulated genes, or ≤ −0.5, for downregulated genes on reads per Kb per million reads (RPKM). Differentially expressed genes were annotated using BLAST similarity searches of the NCBI and FlyBase databases (NCBIResourceCoordinators, 2012; Chiu et al., 2013; Larkin et al., 2021). Differentially expressed genes with olfactory-related functions were identified using gene ontology (GO) terms for biological process: “olfactory behavior”, “olfactory learning”, “sensory perception of chemical stimulus”, or “sensory perception of smell”.

### Flies used for RT-qPCR: transcriptome validation and behavioral experiments

Summer and winter morph *D. suzukii* flies used for validation of the transcriptome analysis and to conduct behavioral assays were obtained from colonies reared at the Rutgers Philip E. Marucci Center for Blueberry and Cranberry Research and Extension (Chatsworth, New Jersey, USA) and Cornell AgriTech (Geneva, New York, USA), respectively. Summer morph flies were kept at standard conditions (25 ± 1 ° C, 16:8 h light:dark, 60–65% humidity). To get winter morph flies, larvae were reared at conditions shown to induce the winter morph (15 ± 1 °C, 12:12 h L:D, 60–65% humidity), according to Wallingford & Loeb (2016) and Stockton, Wallingford & Loeb (2018). Flies were reared on a standard *Drosophila* artificial diet (Dalton et al., 2011; Jaramillo, Mehlferber & Moore, 2015) in 50-mL polystyrene tubes (Fisher Scientific; Nazareth, Pennsylvania, USA) with ∼15 mL of diet and plugged with BuzzPlugs (Fisher Scientific; Nazareth, Pennsylvania, USA). All adult flies were sexually mature (≥ 3 days old) when used in experiments. For RT-qPCR, flies of the same age (between 5 and 7 days old) were taken at the same time of day and immediately stored in a freezer at −80 °C.

### RT-qPCR extraction and purification

Total RNA was extracted following the manufacturer’s protocol using the RNeasy Plant Mini Kit (Qiagen; Hilden, Germany) from heads that were quickly removed with forceps while the flies were kept on dry ice. Samples were ground up using the Qiagen TissueLyser II. RNA quality was assessed using the DeNovix DS-11 FX Spectrophotometer (DeNovix Inc.; Wilmington, Delaware, USA) and gel electrophoresis on the QIAxcel Advanced (Qiagen; Hilden, Germany). We used the Invitrogen SuperScript IV VILO Master Mix with ezDNase Enzyme (ThermoFisher Scientific; Waltham, Massachusetts, USA) to generate cDNA according to the manufacturer’s protocol from 100 ng of total RNA per reaction.

### Transcriptomic validation by RT-qPCR of selected genes

To validate the differential expression in the transcriptome analysis, the expression of two olfactory genes that were most upregulated in the winter morph female heads and one olfactory gene that was one of the most downregulated genes in the winter morph female heads were analyzed using RT-qPCR. Genes chosen for verification were *CheA87a*, *Obp44a*, and *Obp83ef*. Primers were designed using Primer-BLAST (NCBIResourceCoordinators, 2012; https://www.ncbi.nlm.nih.gov/tools/primer-blast/index.cgi). *TBP* was used as the reference (control) gene (Zhai et al., 2014). All primer sets were designed to result in amplicon sizes between 167 and 188 base pairs (Table 1).

For RT-qPCR analysis, cDNAs were synthesized from five biological replicates for the winter morph and five biological replicates for the summer morph, with each biological replicate containing 20 female heads of the respective morph following the manufacturer’s protocol (SuperScript IV VILO Master Mix with ezDNase enzyme; ThermoFisher Scientific; Waltham, Massachusetts, USA).

Reactions for RT-qPCR were performed using the Applied Biosystems QuantStudio 5 RT-qPCR System with 100 ng of cDNA per reaction, 250 nM of forward primer, and 250 nM of reverse primer. Power SYBR Green PCR Master Mix volumes were used according to the manufacturer’s protocol (Applied Biosystems; Foster City, California, USA). Reactions were run under the following conditions: 50 °C for 2 min, 95 °C for 10 min, and 40 cycles at 95 °C for 15 s and 58 °C for 1 min, with the melting curve set at 95 °C for 15 s, 60 °C for 1 min, and 95 °C for 1 s. There were five biological replicates per gene (per morph), and three technical replicates were run per biological replicate. Data were analyzed using the comparative C_T_ method (Schmittgen & Livak, 2008) using the reference gene as an internal control. Statistical differences were determined by comparing ΔCt of each gene between samples using an unpaired *t*-test in Microsoft Excel (2019).

### Behavioral assays

We investigated the differential olfactory response of winter and summer *D. suzukii* morphs to food odors across a range of temperatures (0−25 °C). We used a gated trap assay to measure the fly’s response toward odors from diet, the preferred target of the summer morph flies (Stockton, Cha & Loeb, 2021). A single tube with the bottom cut off was inserted into the lid of a clear 1.5-ounce plastic cup, which acted as a trap and allowed easy entry but difficult exit for the flies. Inside the glass, 5 mL of a standard cornmeal diet was placed to be used as an attractant. Standard diet was used because this was what the flies were reared on, and it was the only food source with which they were experienced; hence, it did not bias results as a novel fruit odor could have. Temperature conditions were manipulated by setting environmental growth chambers at 0 °C, 5 °C, 10 °C, 15 °C, and 25 °C. Prior to the study, the flies were acclimated to their respective temperatures for 72 h.

**Table 1:** List of primers. Primers used for gene expression validation.

**Gene symbol**	**Primers** ^a^	**Amplicon size (bp)**
TBP^b^	F: CCACGGTGAATCTGTGCT R: GGAGTCGTCCTCGCTCTT	186
CheA87a	F: GTGATGGCAGCTATGAGAGGA R: CTTTAGCACACGTACGTCCA	188
Obp44a	F: TGACATCACCCGCAACTACA R: CTTCTGCTCGTTCTTGTCGG	169
Obp83ef	F: ATGGCCTTCTACGATTCCGC R: TCCTGGTACATCCAGGAGCA	167

**Notes.**

a Forward (F), Reverse (R).

b Reference gene.

Assay arenas were set up using polypropylene deli cups (473 mL, 11.7 × 7.6 × 8.9 cm; Pro-Kal, PK165-C; Fabri-Kal, Plastics Place, Kalamazoo, Michigan, USA). The arenas were ventilated by securing the top with mesh fabric and a rubber band. Inside the container, a gated trap was filled with five mL cornmeal diet (Fig. 1B). The gated trap was constructed from polystyrene shot glasses (30 mL, 1.7 × 0.8 × 1.3 cm; Comet, PR384788; Cometware, Chelmsford, Massachusetts, USA). In the middle of the shot glass lid, a 0.6 mL microcentrifuge tube (Fisherbrand; Pittsburg, Pennsylvania, USA) was inserted to “gate” the trap. The last 2 mm of the tip of the tube was shortened to create a diameter just big enough for a fly to enter. Three trials were carried out over a period of three weeks and each trial consisted of a total of four to five temperature treatments with five replicates for each treatment for each morphotype. We visually assessed activity after 24 h using a 4-point rating scale: 0 –least active (chill coma); 1 –standing but not walking; 2 –walking or jumping but not flying; 3 –very active and capable of flying. Survival was assessed after 24 and 48 h. Choice (response) was measured as the number of flies entering the trap out of the total number released.

### Statistical analysis for behavioral data

The behavioral data were analyzed using R i386 (Version 3.0.2; the R Foundation for statistical software R; Vienna, Austria). Generalized linear mixed models (GLMM) with binomial distribution were used to model the effects of fly morphotype and temperature on the proportion of flies captured in the gated trap capture assay and the proportion of flies that survived the assay after 24 h using the package “lme4” and the function *glmer*. Activity level was modeled using a linear mixed model regression and the function *lmer*. Because the assays were repeated serially over time, experimental replicate (date) was treated as a random factor in all models. *Post hoc* mean Tukey comparisons were conducted using the package “emmeans.” For binomial models, the responses are given on the log odds ratio (not the response) scale.

## Results

### Transcriptome analysis

Transcriptome analysis of the head regions of female flies of the winter and summer *D. suzukii* morphs showed significant differences (*p* ≤ 0.0001) in the expression of 737 genes with a log2 fold-change value of ≥ 0.5 (upregulated genes) or ≤ −0.5 (downregulated genes). Of these, 217 genes were upregulated in the heads of female winter morph flies and 520 genes were downregulated in the heads of female winter morph flies. Here, we focused on genes related to olfactory behavior and metabolism since these genes are involved in host and mate location.

**Figure 2:**
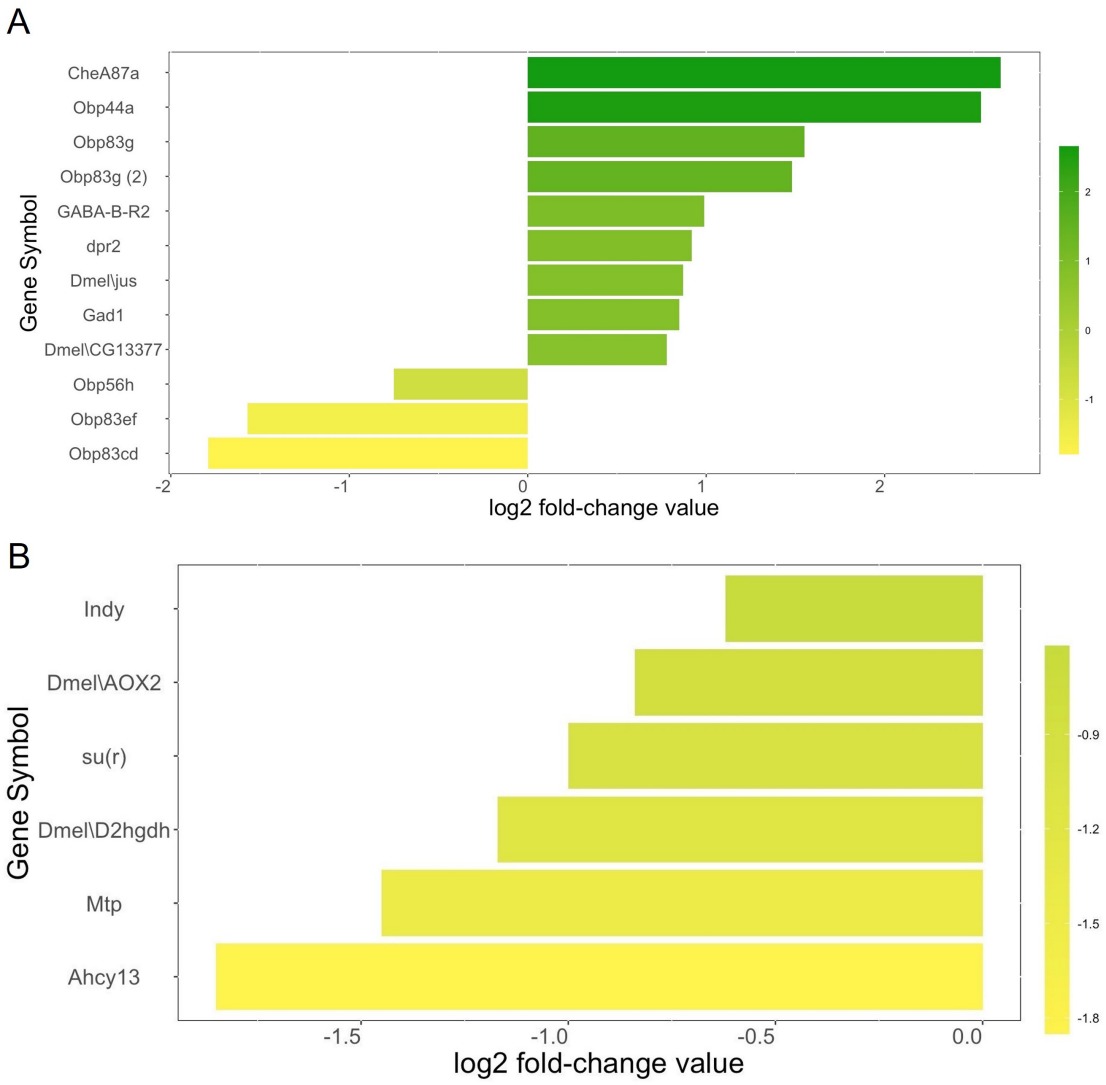
Metabolism genes. (A) Genes related to olfactory behavior and log2 fold-change values according to our transcriptome analysis. Genes that are upregulated in the *Drosophila suzukii* winter morph female heads are towards the top of the chart and in green. Downregulated genes are towards the bottom and in yellow. (B) Selected genes related to metabolism and log2 fold-change values are shown with the same color scheme, with the most downregulated genes towards the bottom and in yellow. Genes with brighter yellows are more strongly downregulated in the winter morph female fly heads.

We identified a total of twelve genes related to olfactory behavior (Fig. 2A). Nine olfactory-related genes (75%) were upregulated in the heads of female winter morph flies while three (25%) were downregulated in the heads of female winter morph flies, a trend opposite of that shown by the overall transcriptome analysis, where most genes tended to be downregulated in the winter morph female heads.

Among the differentially expressed genes linked to metabolism, and with a known function in either FlyBase or SpottedWingFlyBase, 82 were listed with the keyword “metabolic” (see annotation, Data S1). Of these genes, 74% (61) were downregulated in the female winter morph heads. Notably, we found six of the differentially expressed metabolism genes of interest in the transcriptome analysis that were downregulated in the female winter morph heads (Fig. 2B).

### RT-qPCR validation

**Figure 3:**
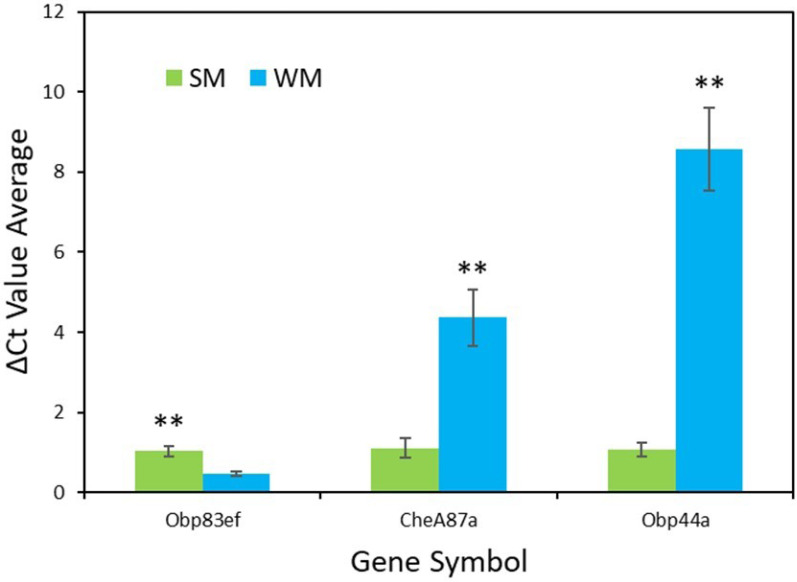
qPCR validation of genes identified in transcriptome analysis. The graph shows the average ΔCt values of the five RT-qPCR replicates for each morph (Data S2). Higher ΔCt values indicate upregulation in either the summer morph (SM, green bars) or the winter morph (WM, blue bars). SEM error bars are shown. Differences in relative expression of *Obp83ef*, *CheA87a*, and *Obp44a* were statistically significant at *p* ≤ 0.01 (*p* = 0.01, 0.007, and 0.003, respectively) and are marked with asterisks (**).

RT-qPCR validation confirmed the results from the transcriptome analysis for the genes *Obp83ef* (*t* =  − 4.0, *df* = 5, *p* = 0.01), *CheA87a* (*t* = 4.4, *df* = 5, *p* = 0.007), and *Obp44a* (*t* = 6.4, *df* = 4, *p* = 0.003) (Fig. 3). All genes were found to be expressed as expected, though *Obp44a* was more strongly upregulated than *CheA87a*, a slight difference from the transcriptome analysis. Average ΔCt values from five replicates for each gene are as follows: *Obp83ef* winter morph 0.5, summer morph 1.0; *CheA87a* winter morph 4.4, summer morph 1.1; and *Obp44a* winter morph 8.6, summer morph 1.1 (Data S2).

### Behavioral assays

**Figure 4:**
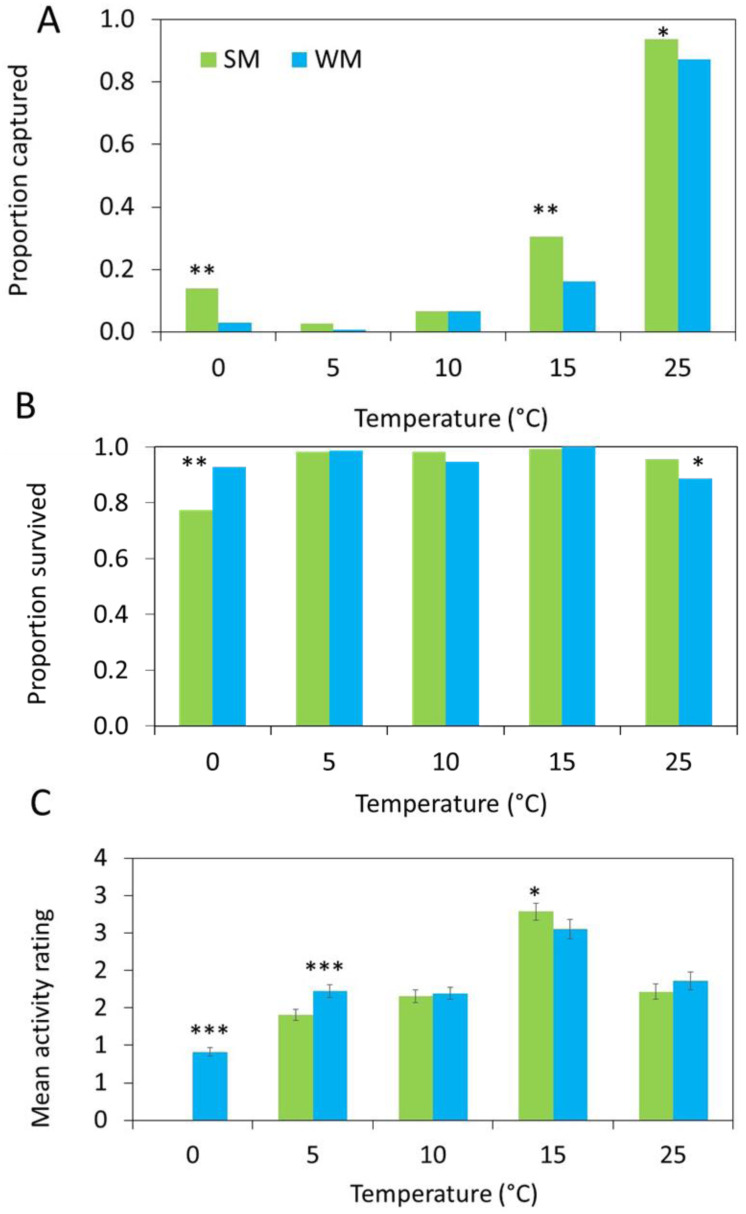
Behavioral data show differences between morphs. (A) In each arena, 10 flies were released, and three different response measures were recorded after 24 hours (*n* = 15 replicates). First, we measured attraction to the diet and recorded the proportion of summer morph (SM, green bars) and winter morph (WM, blue bars) flies that were captured in each trap. (B) Second, we recorded the proportion of flies that survived the assay. Generalized linear mixed models (GLMM) with binomial distribution were used to model the effects of phenotype on both trap capture and survival. (C) Lastly, we observed the mean activity level of 25 flies per treatment using a 4-point rating scale. Differences in activity level were compared between SM and WM flies using linear mixed model regression. Asterisks indicate statistically significant differences between SM and WM responses for all figures based on Tukey posthoc mean comparisons: * ≤ 0.05, ** ≤ 0.01, *** ≤ 0.001.

The number of flies captured in the gated trap capture assay increased with temperature (Table 2) and the highest proportion of flies entered the traps at 25 °C (Fig. 4A). More summer morph flies entered the traps than winter morph flies, even at lower temperatures (Table 2).

**Table 2:** The effect of temperature on activity of *Drosophila suzukii* flies. The effect of temperature and morphotype variation on the response, survival, and activity of *Drosophila suzukii* flies.

	**Fixed Effects**	*χ* ^2^	**df**	*p* >*χ*^2^
Response^a^	Temperature^*^	334.64	4	<0.001
	Morphotype^*^	15.45	1	<0.001
	Temp × Morph	5.50	4	0.239
Survival^a^	Temperature*	41.97	4	<0.001
	Morphotype	0.02	1	0.897
	Temp × Morph^*^	17.03	4	0.002
Activity^b^	Temperature^*^	455.24	4	<0.001
	Morphotype^*^	7.45	1	0.006
	Temp × Morph^*^	41.04	4	<0.001

**Notes.**

a Based on GLMM with binomial distribution.

b Based on linear mixed model regression.

* Indicates significantly different effects (*p* ≤ 0.05).

Survival was high for both morphotypes, except at freezing where the survival of the summer morph flies was reduced relative to the winter morphs, leading to a significant interaction between temperature and morphotype (Table 2; Fig. 4B).

Winter morph flies were significantly more active than the summer morph flies at 0 °C and 5 °C, while at warmer temperatures winter morph activity was similar or lower compared to the summer flies (Table 2; Fig. 4C).

## Discussion

In the present study, we combined molecular and behavioral approaches to understand differences in olfactory responses between the winter and summer morphs of *D. suzukii*. Our molecular studies revealed that several olfactory genes related to food and mate location are differentially expressed in winter versus summer morph *D. suzukii* flies. In behavioral studies, we demonstrated that, although winter morph flies were more active at lower temperatures, the summer morph flies were generally more attracted to food odors.

Several of the differentially expressed genes related to olfactory behavior we found have been linked to food-seeking and mating behaviors. *CheA87a* has been implicated in sex-specific pheromone detection among *Drosophila* flies, and the gene may have been involved in evolutionary changes in host plant preference between *Drosophila simulans* (Sturtevant) and *Drosophila sechellia* (Tsacas & Bachli) (Shiao et al., 2015). *CheA87a* is the most upregulated gene in the heads of female winter morph *D. suzukii* flies, and its potential link to host plant preference in closely related species (Shiao et al., 2015) suggests that it might influence olfactory predilection in *D. suzukii*. Inhibition of *Obp56h* decreases the appetite of *Drosophila melanogaster* (Meigen) for consuming bitter foods (Swarup et al., 2014). *Obp56h* is downregulated in the heads of female winter morph *D. suzukii* flies; thus, they may be less attracted to bitter compounds while diapausing in winter, a finding consistent with the idea that the winter morph has food preferences distinct from the summer morph (Stockton, Brown & Loeb, 2019).

Seven of the differentially expressed olfactory-related genes we found are odorant binding proteins. While the function of these proteins is still being investigated, they may influence the rate at which odorants diffuse into the sensilla (Zhao & McBride, 2020). After mating, female *D. suzukii* flies exhibit broad upregulation in several classes of olfactory-related genes, including odorant binding proteins, possibly to improve the female flies’ abilities to locate fruit and oviposition sites (Crava et al., 2019). Given the potential importance of differences in olfactory-related gene transcription after mating for resource-seeking behavior, it is probable that the differences in transcription between the winter and the summer morph may also be representative of differences in food- or shelter-seeking behavior. Indeed, our behavioral experiments show that winter morph flies appeared less motivated to seek food, despite overall higher activity levels than summer morph flies at low temperatures.

Analysis of the transcriptome data suggests that winter morph *D. suzukii* flies should be less active due to the broad downregulation of metabolism-related genes. The six metabolism genes of interest play a variety of metabolic roles (Fig. 2B). For example, *Indy* is important in the Krebs cycle, and downregulation of this gene leads to increased longevity (Willmes & Birkenfeld, 2013). The gene *su(r)* is important in pyrimidine catabolism and for the production of NADP+, and it is linked to changes in diet sensitivity and cuticle coloration (Data S3; Rawls, 2006).

Two vitellogenin genes related to yolk production, *Yp1* and *Yp2*, were downregulated in the female winter morph heads (Data S3), indicating potential differences in nutritional signaling and reproductive competency between the two morphs (Søndergaard et al., 1995). Downregulation of *Yp1* and *Yp2* has been negatively correlated with longevity; moreover, these genes are crucial for yolk deposition (Tarone et al., 2012) and could thus ultimately be correlated with a differential response to olfactory cues. It would be of interest to investigate the role of yolk proteins in nutritional signaling between the summer and winter *D. suzukii* morphs to indicate response to nutritional resources.

Our behavioral results dovetail with the findings of the transcriptome analysis. Despite high survival at all temperatures tested, there were significant effects of temperature on movement (*i.e.*, activity) and diet selection in this study. Our findings indicate that winter morph flies have the capacity to be active during cool winter and early spring conditions; however, they may also indicate shifts in the ecological priorities of *D. suzukii* depending on morphotype. We used standard cornmeal diet as the bait in our behavioral experiments to avoid biasing results, and the high response rate of flies of both morphotypes in warm conditions indicates that the bait was effective. It would be interesting to assess the effects of different baits such as fruit odors. It could also be worthwhile to assess whether or not differences in rearing temperatures contribute to significantly different winter morph transcriptomes—the flies used for behavioral studies and the transcriptome validation were reared at 15 °C while those used for our transcriptome analysis were reared at 10 °C (see Shearer et al., 2016 for rearing methods of flies used for the transcriptome analysis; see Wallingford & Loeb, 2016 for detailed rearing methods used also in this study). Given the overall congruence of our results and that all flies exhibit the darker phenotype characteristic of the winter morph, it appears likely that the winter morphotype can be induced by a broad range of temperatures.

While summer morph flies develop under warm temperatures and are primarily motivated to seek food and reproduce, winter morph flies may have suppressed metabolic function, requiring less food to survive (Stockton, Brown & Loeb, 2019). For this reason, seeking sheltered microclimates may be more important for winter morph flies during cool conditions. This conclusion from the behavioral assays is based on captures relative to activity level. More winter morph flies should have entered the gated trap at lower temperatures due to their higher activity level at those temperatures. As this outcome is not what we recorded, it seems that the winter morph flies are less motivated to seek out food. Although flies could both fly and walk into the trap, it is possible that the height of the trap opening biased results toward flying insects. A lower lying trap that allows winter morph flies to more easily walk in could make for an interesting follow-up study, especially if it could be conducted in combination with field-assays of ground-level traps.

Current tools for monitoring *D. suzukii* adult populations during the growing season, *i.e.*, summer and fall, rely on food-based odors derived from fermentation products (Burrack et al., 2015; Cha et al., 2018; Cloonan et al., 2019). Data from these previous studies suggest that winter morph flies may not be strongly attracted to standard trapping systems baited with food odors, potentially leading to an underestimation of their population. Research evaluating the effects of alternative diet sources on the different *D. suzukii* morphotypes has helped to better understand the feeding dynamics of this species (Fountain et al., 2018; Rendon et al., 2018). Our study contributes to these previous studies by showing significant differential expression of olfactory-related genes and behavioral responses to food odors between the winter and summer *D. suzukii* morphs. We also note there is a discrepancy in the differential expression of *CheA87a* in our study that was not identified in the Shearer et al. (2016) study. This emphasizes the importance of reanalyzing transcriptome data as bioinformatic tools improve and newer pipelines for analysis are developed. Further research should be directed towards understanding metabolic priorities of winter morph flies to be able to develop winter morph-specific attractants that growers could use for IPM monitoring during the offseason. Future investigations will also be needed to understand the network of factors that drive the attraction of winter *D. suzukii* morphs to food sources, *i.e.*, visual cues, olfactory sensory abilities, or a combination of both.

##  Supplemental Information

10.7717/peerj.13825/supp-1 Supplemental Information 1 Transcriptome Analysis Fold change values for genes identified as significantly different in bioinformatic analysis

10.7717/peerj.13825/supp-2 Supplemental Information 2 Gene Annotations Gene annotation categories distinguisged for each differentially expressed gene found in transcriptome

10.7717/peerj.13825/supp-3 Supplemental Information 3 Oflactory Genes in Bioinformatic Analysis Olfactory related genes with ID from gene databases

10.7717/peerj.13825/supp-4 Supplemental Information 4 Transcriptome Raw Data

10.7717/peerj.13825/supp-5 Supplemental Information 5 Raw data of the behavioral analysis

10.7717/peerj.13825/supp-6 Supplemental Information 6 Raw qPCR data from gene validation

## Additional Information and Declarations

### Data Availability

The following information was supplied regarding data availability: The raw data from qPCR analysis and behavioral analysis are available in the Supplementary Files.
