# Supplementary material for: Molecular and behavioral studies reveal differences in olfaction between winter and summer morphs of Drosophila suzukii
Source: PeerJ. 2022 Sep 16;10:e13825. doi: 10.7717/peerj.13825 (PMC9484457; doi:10.7717/peerj.13825)
Supplement: Supplemental Information 1 — Fold change values for genes identified as significantly different in bioinformatic analysis [file peerj-10-13825-s001.docx]

Gene annotations for all genes with a *p*-value of less than or equal to 0.0001. Annotations generated from information on SpottedWingFlyBase (Process) and FlyBase (*Drosophila melanogaster*, DM Ortholog).

| **Gene ID** | **Gene Name** | **Process** | **P-Value** | **DM Ortholog** |
| --- | --- | --- | --- | --- |
| DS10_00011701 | eff | Molecular function: protein binding; ubiquitin-protein ligase activity. Biological process: single-organism developmental process; biological regulation; cellular component organization or biogenesis; sensory organ development; catabolic process; cell cycle; regulation of developmental process; primary metabolic process; neuron differentiation; multicellular organism reproduction; macromolecule catabolic process. | 2.93626594863274e-89 | effete ([eff](http://flybase.org/search/eff)) effete ([eff](http://flybase.org/search/eff)) encodes a conserved class I E2 ubiquitin-conjugating enzyme that contributes to the protein ubiquitination and degradation pathway. The roles of the product of [eff](http://flybase.org/search/eff) include female germline stem cell maintenance, eye development, apoptosis regulation and chromatin organization. |
| DS10_00000162 | Adhr | Molecular function: alcohol dehydrogenase (NAD) activity; nucleotide binding. Biological process: oxidation-reduction process. | 5.90868814874962e-65 | Adh-related (CG3484) [Function not well-known] |
| DS10_00006773 | N/A | Molecular function is unknown. Biological process is unknown. | 8.00558613657813e-45 |  |
| DS10_00005973 | N/A | Molecular function is unknown. Biological process is unknown. | 9.12927908815363e-35 | FBgn0029990; Dmel\CG2233 |
| DS10_00004670 | per | Molecular function: protein binding; transcription factor binding; protein heterodimerization activity. Biological process: biological regulation; rhythmic process; response to stimulus; multi-organism reproductive process; response to stress; mating; single-organism developmental process; multicellular organismal development; learning or memory; determination of adult lifespan; locomotory behavior; age-dependent response to oxidative stress. | 1.58763001596745e-34 | Dmel\per Females heterozygous for per+ and a deletion of the locus or a per0 allele show longer-than-normal periods. per flies can be classified on the basis of their circadian rhythms as: (1) Cryptic period mutants (per0, per-) which have a 10-15 hr (ultradian) period and appear arrhythmic except in special algorhythmic tests (Dowse et al., 1987); (2) Long period mutants (perL), 29 hr; (3) Long-period variable mutants (perLvar), which in homozygotes or heterozygotes are arrhythmic but in combination with certain partial deletions of the per locus result in a 30-34 hr period. (Konopka, 1987); (4) Short period mutants (pers), 19 hr; (5) Short period variable mutants (persvar), some flies having a 20 hr period and the others a normal 24 hr period for locomotor activity. In temperature-change experiments on pers and perL1, the locomotor activity periods were found to be nearer to 24 hr at low temperatures, but to diverge further from normal upon heating (Konopka, Pittendrigh, and Orr; Hamblen, Ewer, and Hall). perL2 shows lengthening of the periods at high temperatures. The mutant types affecting circadian rhythms (per0, perL, and pers) may cause similar kinds of changes in the rhythmic fluctuations in courtship song interpulse intervals (IPIs) of the male (Crossley, 1988; Ewing, 1988; Kyriacou and Hall, 1980, 1986, 1988). per0 mutants show nonrhythmic variations in the interval between pulses of wing vibration. |
| DS10_00009142 | Cpr100A | Molecular function: structural constituent of chitin-based cuticle. Biological process is unknown. | 7.96931333271105e-31 | Dmel\Cpr100A |
| DS10_00004890 | Yp1 | Molecular function: structural molecule activity. Biological process: vitellogenesis. | 4.0038961584228e-29 | Dmel\Yp1 Structural gene for the yolk protein YP1 found in recently-emerged female flies. Protein migrates at different rates in SDS-polyacrylamide gels when encoded by the electrophoretic variants Yp1F (fast) and Yp1S (slow), alleles that are female fertile and produce normal amounts of YP1. Yp1ts1, which maps near the Yp1 locus and is believed to be an allele, produces a slow-migrating translation product that is present in reduced amounts in the hemolymph and the ovaries (Bownes and Hodson, 1980); this mutant is female sterile.  Its molecular function is described by: lipase activity; carboxylic ester hydrolase activity. It is involved in the biological process described with: sex differentiation; lipid catabolic process. |
| DS10_00004891 | Yp2 | Molecular function: structural molecule activity. Biological process: neurogenesis. | 2.13292529170438e-28 | Dmel\Yp2 carboxylic ester hydrolase activity; lipase activity. It is involved in the biological process described with: lipid catabolic process; sex differentiation.  Structural gene for the yolk protein YP2 found in recently-emerged female flies. Protein migrates at different rates in SDS-polyacrylamide gels when encoded by the electrophoretic variants Yp2F (fast) and Yp2S (slow), alleles that are female fertile and produce normal amounts of YP2. A mutant Yp2M (=Yp212-1245) is female fertile but lays fewer eggs than normal (Mohler, Postlethwait, and Shirk) and does not contain yolk protein in the hemolymph or ovaries.  Vitellogenin is the major yolk protein of eggs where it is used as a food source during embryogenesis. |
| DS10_00006272 | N/A | Molecular function: choline dehydrogenase activity. Biological process: oxidation-reduction process; alcohol metabolic process. | 7.40818805213837e-28 | Dmel\CG9521 |
| DS10_00000247 | vri | Molecular function: RNA polymerase II core promoter proximal region sequence-specific DNA binding transcription factor activity involved in negative regulation of transcription. Biological process: cellular process; circadian rhythm; chaeta morphogenesis; open tracheal system development; imaginal disc-derived wing hair organization. | 1.04368955162501e-26 | Dmel\vri  vrille ([vri](http://flybase.org/search/vri)) encodes a bZIP transcription factor acting as an enhancer of dpp phenotypes both in embryo and in wing. It is involved in hair and cell growth and in tracheal development. Vri is a clock-controlled gene acting as a repressor of the products of [Clk](http://flybase.org/search/Clk) and [cry](http://flybase.org/search/cry). [Date last reviewed:2019-03-21] |
| DS10_00010719 | Nplp3 | Molecular function: neuropeptide hormone activity. Biological process: neuropeptide signaling pathway. | 1.43050117820826e-26 | Dmel\Nplp3  Neuropeptide-like precursor 3 |
| DS10_00012411 | N/A | Molecular function: argininosuccinate synthase activity. Biological process: argininosuccinate metabolic process. | 2.61616177507011e-26 | Dmel\Ass |
| DS10_00006843 | N/A | Molecular function is unknown. Biological process is unknown. | 1.31946883110005e-25 | Dmel\CG15347 |
| DS10_00004445 | Cpr62Bb | Molecular function: structural constituent of chitin-based cuticle. Biological process is unknown. | 4.16277585925895e-25 | Dmel\Cpr62Bb |
| DS10_00006717 | Obp44a | Molecular function: odorant binding. Biological process: sensory perception of chemical stimulus. | 1.69259074585476e-24 | Dmel\Obp44a |
| DS10_00002426 | Ef1alpha48D | Molecular function: translation elongation factor activity. Biological process: determination of adult lifespan. | 4.94151209992811e-24 | Multiple; see work on Wednesday, April 22 in this notebook |
| DS10_00007068 | Rfabg | Molecular function: retinol binding; lipid transporter activity; receptor binding; microtubule binding; heme binding. Biological process: smoothened signaling pathway; lipid transport; Wnt receptor signaling pathway. | 7.50916037417084e-24 | Dmel\apolpp  apolipophorin ([apolpp](http://flybase.org/search/apolpp)) encodes an apolipoprotein of the ApoB family. It is complexed with lipids to assemble the major hemolymph lipoprotein, lipophorin. Lipophorin transports neutral lipids, phospholipids and sterols between tissues. Lipophorin also carries lipophilic hormones and signaling molecules. [Date last reviewed: 2019-03-07] |
| DS10_00006763 | Mal-A5 | Molecular function: catalytic activity; cation binding. Biological process: carbohydrate metabolic process | 2.73605450144372e-23 | Dmel\Mal-A5 maltose alpha-glucosidase activity. It is involved in the biological process described with: carbohydrate metabolic process. |
| DS10_00012848 | N/A | Molecular function: glycine C-acetyltransferase activity. Biological process: cellular amino acid metabolic process | 1.75709759118584e-22 | Dmel\CG10361 |
| DS10_00008400 | Yp3 | Molecular function: structural molecule activity. Biological process: embryo development; neurogenesis. | 2.58852522071809e-22 | Dmel\Yp3 |
| DS10_00011418 | N/A | Molecular function: transketolase activity. Biological process: regulation of chromatin silencing; cytoplasmic microtubule organization. | 8.45032802136262e-22 | Dmel\CG8036 |
| DS10_00005484 | Tal | Molecular function: sedoheptulose-7-phosphate:D-glyceraldehyde-3-phosphate glyceronetransferase activity. Biological process: pentose-phosphate shunt, non-oxidative branch. | 9.81331192082055e-22 | Dmel\Taldo |
| DS10_00007451 | N/A | Molecular function: structural constituent of cuticle. Biological process is unknown. | 9.69466067572376e-22 | Dmel\CG8927 |
| DS10_00004647 | TRAM | Molecular function is unknown. Biological process: phagocytosis, engulfment. | 1.61122785792172e-21 | Dmel\TRAM |
| DS10_00011507 | N/A | Molecular function: glycine N-methyltransferase activity. Biological process is unknown. | 1.92044073586051e-21 | Dmel\Gnmt  Glycine N-methyltransferase ([Gnmt](http://flybase.org/search/Gnmt)) encodes an enzyme that catalyzes the methylation reaction of glycine to generate sarcosine (N-methylglycine). It is abundantly expressed in the fat body and controls the amount of methyl donor S-adenosylmethionine. [Date last reviewed: 2019-03-07] |
| DS10_00013076 | verm | Molecular function: chitin binding; chitin deacetylase activity. Biological process: dorsal trunk growth, open tracheal system; trachea morphogenesis; visual behavior; open tracheal system development; regulation of tube length, open tracheal system. | 1.50726754777535e-20 | Dmel\verm  vermiform ([verm](http://flybase.org/search/verm)) encodes a chitin deacetylase-like protein that is secreted by tracheal cells and accumulates in the embryonic tracheal lumen. Its functions include cuticle development and tracheal tube size control. [Date last reviewed: 2019-03-21] |
| DS10_00007513 | N/A | Molecular function: oxidoreductase activity; oxidoreductase activity, acting on the CH-OH group of donors, NAD or NADP as acceptor. Biological process: metabolic process. | 1.63763746920319e-20 | Dmel\CG40486 |
| DS10_00012395 | retinin | Molecular function is unknown. Biological process is unknown. | 1.72728548066233e-20 | Dmel\retinin |
| DS10_00006716 | Pabp2 | Molecular function: RNA binding; poly(A) RNA binding. Biological process: mRNA polyadenylation; nuclear-transcribed mRNA poly(A) tail shortening; neurogenesis |  | Dmel\Pabp2  Pabp2 ([Pabp2](http://flybase.org/search/Pabp2)) encodes a nuclear poly(A) binding protein that is involved in both the cleavage and polyadenylation steps of the nuclear cleavage/polyadenylation reaction. Cytoplasmic protein encoded by [Pabp2](http://flybase.org/search/Pabp2) acts to shorten the poly(A) tails of specific mRNAs in oocytes and syncytial blastoderm embryos. [Date last reviewed: 2018-09-13] |
| DS10_00012387 | Cpr72Ec | Molecular function: structural constituent of chitin-based cuticle. Biological process is unknown. | 8.10060215845872e-20 | Dmel\Cpr72Ec |
| DS10_00001532 | N/A | Molecular function: neurotransmitter transporter activity. Biological process: neurotransmitter transport. | 9.94605058428118e-20 | Dmel\CG13795 |
| DS10_00010584 | Tig | Molecular function is unknown. Biological process: axon guidance; cell-substrate adhesion; substrate adhesion-dependent cell spreading; regulation of cell adhesion mediated by integrin; cell adhesion mediated by integrin; phagocytosis, engulfment. | 4.32910338582061e-19 | Dmel\Tig  Tiggrin ([Tig](http://flybase.org/search/Tig)) is an extracellular matrix protein and integrin ligand that accumulates at the embryonic and larval muscle attachment site. It is also found as a component of the hemolymph clot. [Date last reviewed: 2018-09-13] |
| DS10_00005788 | N/A | Molecular function is unknown. Biological process is unknown. | 6.01768151259219e-19 | Dmel\CG15080 |
| DS10_00002806 | Ndg | Molecular function: calcium ion binding. Biological process: cell-matrix adhesion. | 9.07338610283592e-19 | Dmel\Ndg |
| DS10_00002372 | Pu | Molecular function: GTP cyclohydrolase I regulator activity; GTP cyclohydrolase I activity. Biological process: preblastoderm mitotic cell cycle; regulation of GTP cyclohydrolase I activity; cuticle pigmentation; pteridine biosynthetic process; regulation of epithelial cell migration, open tracheal system; embryonic pattern specification; compound eye pigmentation; larval chitin-based cuticle development. | 5.45887668163091e-18 | Dmel\Pu  Punch! |
| DS10_00010769 | Bap55 | Molecular function: transcription coactivator activity. Biological process: positive regulation of gene silencing by miRNA; dendrite morphogenesis; mitosis; cytokinesis; muscle organ development; positive regulation of transcription, DNA-dependent; dendrite guidance. | 6.24315255258891e-18 | Dmel\Bap55  Brahma associated protein 55kD ([Bap55](http://flybase.org/search/Bap55)) encodes a member of two chromatin remodeling complexes. As part of the Brahma complex, it is needed for cell growth and survival in the wing imaginal disc; as a member of the TIP60 complex, it is thought to regulate dendrite wiring specificity in olfactory projection neurons. [Date last reviewed: 2018-10-18] |
| DS10_00001733 | N/A | Molecular function is unknown. Biological process is unknown | 1.13701276523023e-17 | Dmel\Vajk3 |
| DS10_00012599 | Nplp3 | Molecular function: neuropeptide hormone activity. Biological process: neuropeptide signaling pathway. | 1.88343911546013e-17 | Dmel\Nplp3 |
| DS10_00004328 | N/A | Molecular function: CDP-diacylglycerol-serine O-phosphatidyltransferase activity. Biological process: neurogenesis. | 3.05161594567972e-17 | FLy Base Hit List; see Sunday, April 26, 2020 for table |
| DS10_00002541 | No results | No results; used BLAST on nucleotide sequence - most similar to an uncharacterized protein coding DNA in *D. simulans* | 3.56817876359675e-17 | No results |
| DS10_00009650 | N/A | Molecular function: xylulokinase activity. Biological process: carbohydrate metabolic process. | 3.98341594058037e-17 | Dmel\CG3534  Xylulokinase |
| DS10_00003021 | Nurf-38 | Molecular function: inorganic diphosphatase activity. Biological process: ecdysone receptor-mediated signaling pathway; transcription, DNA-dependent; nucleosome mobilization; positive regulation of Notch signaling pathway; regulation of hemocyte proliferation; chromatin remodeling. | 4.22266616855064e-17 | Dmel\Nurf-38  Nucleosome remodeling factor - 38kD  Inorganic diphosphatase |
| DS10_00006834 | Tps5D | Molecular function is unknown. Biological process is unknown. | 4.33580068051974e-17 | Dmel\Tsp5D  Tetraspanin 5D |
| DS10_00001452 | N/A | Molecular function: D-aspartate oxidase activity. Biological process: oxidation-reduction process. | 2.2375536313757e-16 | Dmel\CG11236  D-amino-acid oxidase |
| DS10_00006223 | Ahcy13 | Molecular function: adenosylhomocysteinase activity. Biological process: one-carbon metabolic process. | 4.15368845535494e-16 | Dmel\Ahcy  Adenosylhomocysteinase ([Ahcy](http://flybase.org/search/Ahcy)) encodes S-adenosyl-L-homocysteine hydrolase, the rate-limiting enzyme in methionine metabolism. This tetrameric enzyme catalyzes the reversible hydrolysis of S-Adenosylhomocysteine (SAH) to adenosine and L-homocysteine. The function of [Ahcy](http://flybase.org/search/Ahcy) product is required to maintain proper concentrations of SAH, which serves as an inhibitor of S-adenosylmethionine-dependent methylation reactions. [Date last reviewed: 2018-09-13] |
| DS10_00000924 | N/A | Molecular function: serine-type endopeptidase activity. Biological process: proteolysis. | 4.40474853963304e-16 | TWO: 1) protein coding gene: Dmel\CG18477  2) protein coding gene: Dmel\CG31780 |
| DS10_00005787 | N/A | Molecular function: 3-hydroxyisobutyrate dehydrogenase activity. Biological process: cellular amino acid metabolic process. | 6.70603745926029e-16 | Dmel\CG15093  3-hydroxyisobutyrate dehydrogenase  Protein coding gene |
| DS10_00001472 | ade3 | Molecular function: phosphoribosylamine-glycine ligase activity; phosphoribosylformylglycinamidine cyclo-ligase activity. Biological process: 'de novo' IMP biosynthetic process. | 1.35677831396515e-15 | Dmel\Gart  GART trifunctional enzyme ([Gart](http://flybase.org/search/Gart)) encodes a multifunctional enzyme that exhibits phosphoribosylamine-glycine ligase, phosphoribosylformylglycinamidine cyclo-ligase and phosphoribosylglycinamide formyltransferase activities. [Date last reviewed: 2019-09-12] |
| DS10_00011638 | serp | Molecular function: chitin binding; chitin deacetylase activity. Biological process: open tracheal system development; regulation of tube length, open tracheal system. | 1.737618152546e-15 | Dmel\serp  serpentine ([serp](http://flybase.org/search/serp)) encodes a putative chitin deacetylase. It is expressed by the epidermal and tracheal cells and is secreted into the apical extracellular space. Its activity maintains the strength of cuticles and restricts tracheal tube length. [Date last reviewed: 2019-03-14] |
| DS10_00013161 | N/A | Molecular function: metallocarboxypeptidase activity. Biological process: proteolysis. | 2.09193563618856e-15 | Dmel\CG4408 |
| DS10_00008965 | N/A | Molecular function: phosphatidylserine decarboxylase activity. Biological process: phospholipid biosynthetic process. | 2.55349061478552e-15 | Dmel\Pisd  Phosphatidylserine decarboxylase  Protein coding gene |
| DS10_00000293 | Cyp309a2 | Molecular function: electron carrier activity. Biological process: oxidation-reduction process. | 6.30844960365998e-15 | Dmel\Cyp309a2 |
| DS10_00001002 | fbp | Molecular function: fructose 1,6-bisphosphate 1-phosphatase activity. Biological process: carbohydrate metabolic process. | 6.34295311097597e-15 | Dmel\fbp  Fructose-1,6-bisphosphatase  Protein coding gene |
| DS10_00007357 | N/A | Molecular function: oxoglutarate dehydrogenase (succinyl-transferring) activity. Biological process: tricarboxylic acid cycle. | 9.77447814598773e-15 | Dmel\CG1544  Protein coding gene  Oxoglutarate dehydrogenase (succinyl-transferring) |
| DS10_00003597 | Ance-5 | Molecular function: peptidyl-dipeptidase activity. Biological process: proteolysis. | 1.06755043861554e-14 | Dmel\Ance-5  Protein coding gene |
| DS10_00010034 | N/A | Molecular function: serine-type endopeptidase activity. Biological process: proteolysis | 1.05974364017953e-14 | Dmel\CG14990 |
| DS10_00009067 | N/A | Molecular function: aldose 1-epimerase activity. Biological process: hexose metabolic process. | 1.1510459258589e-14 | Dmel\CG32444  Aldose 1-epimerase |
| DS10_00009435 | Mlc2 | Molecular function: microfilament motor activity; calcium ion binding. Biological process: muscle system process. | 1.19684869507434e-14 | Dmel\Mlc2  Myosin light chain 2 ([Mlc2](http://flybase.org/search/Mlc2)) encodes a subunit of the myosin complex involved in myofibril assembly. It contributes to different flight-related processes including wing beat frequency, and indirect flight muscle contraction. [Date last reviewed: 2019-07-11] |
| DS10_00001732 | N/A | Molecular function is unknown. Biological process is unknown. | 1.22886642875583e-14 | Dmel\Vajk2 |
| DS10_00012965 | No Results | No results;  Used BLAST, top match from *D. suzukii*:  chymotrypsin-like protease CTRL-1 | 2.03211012489828e-14 | No results |
| DS10_00008644 | Tctp | Molecular function: guanyl-nucleotide exchange factor activity; Ras guanyl-nucleotide exchange factor activity; Ras GTPase binding. Biological process: positive regulation of cell size; positive regulation of multicellular organism growth. | 2.24456640700121e-14 | Dmel\Tctp  Translationally controlled tumor protein ([Tctp](http://flybase.org/search/Tctp)) encodes a protein that belongs to the TCTP family. It interacts with the GTPase encoded by [Rheb](http://flybase.org/search/Rheb) to regulate organ growth. It is required for DNA damage response and genome stability. [Date last reviewed: 2019-03-14] |
| DS10_00001969 | Cry | Molecular function: structural constituent of eye lens. Biological process is unknown. | 2.32530358136801e-14 | Dmel\Crys  Crystallin ([Crys](http://flybase.org/search/Crys)) encodes a chitin binding protein involved in the formation of the peritrophic matrix, a chitinous layer lining the midgut. [Date last reviewed: 2019-03-07] |
| DS10_00003383 | trpl | Molecular function: protein binding; calmodulin binding; protein heterodimerization activity; calcium channel activity; light-activated voltage-gated calcium channel activity. Biological process: sensory perception of sound; calcium-mediated signaling; calcium ion transmembrane transport; phototransduction, visible light; body fluid secretion; calcium ion transport; cellular response to anoxia. | 3.51434598133699e-14 | Dmel\trpl  transient receptor potential-like ([trpl](http://flybase.org/search/trpl)) encodes a non-selective plasma membrane cation channel with modest Ca[2+] permeability. It is highly eye-enriched and contributes to the electrical response to light in photoreceptors. [Date last reviewed: 2019-03-14] |
| DS10_00002421 | N/A | Molecular function is unknown. Biological process: regulation of transcription, DNA-dependent. | 4.72447834975635e-14 | Dmel\exp  expansion ([exp](http://flybase.org/search/exp)) encodes an atypical Smad-like protein which regulates tube size in the trachea through receptor tyrosine kinase (RTK) pathways, as well as apical secretion pathways. [Date last reviewed: 2019-03-07] |
| DS10_00013455 | Pxd | Molecular function: peroxidase activity. Biological process: neurogenesis; phagocytosis, engulfment. | 4.90300783459366e-14 | Dmel\Pxd  Peroxidase ([Pxd](http://flybase.org/search/Pxd)) encodes an enzyme that converts hydrogen peroxide to water and molecular oxygen, in the process oxidizing glutathione to glutathione disulfide. It is involved in the cellular response to oxidative stress. [Date last reviewed: 2019-03-14] |
| DS10_00005003 | N/A | Molecular function: oxidoreductase activity, acting on the CH-OH group of donors, NAD or NADP as acceptor. Biological process: metabolic process. | 7.55172440854603e-14 | Dmel\CG10962  3-beta-hydroxysteroid 3-dehydrogenase ([1.1.1.270](https://enzyme.expasy.org/EC/1.1.1.270))  17-beta-estradiol 17-dehydrogenase ([1.1.1.62](https://enzyme.expasy.org/EC/1.1.1.62)) |
| DS10_00006251 | N/A | Molecular function: dihydrolipoamide branched chain acyltransferase activity. Biological process: phagocytosis, engulfment. | 1.35358481642677e-13 | Dmel\CG5599 |
| DS10_00009184 | Cpr97Ea | Molecular function: structural constituent of chitin-based cuticle. Biological process is unknown. | 1.44585529477985e-13 | Dmel\Cpr97Ea  Cuticular protein 97Ea |
| DS10_00001278 | fon | Molecular function is unknown. Biological process: hemolymph coagulation; metamorphosis | 1.50030899487202e-13 | Dmel\fon  fondue ([fon](http://flybase.org/search/fon)) encodes a protein produced by the fat body that is involved in the clotting reaction. [Date last reviewed: 2019-03-07] |
| DS10_00004123 | N/A | molecular function is unknown. Biological process: regulation of alternative mRNA splicing, via spliceosome; neurogenesis. | 1.74602377453172e-13 | Dmel\CG10418 |
| DS10_00008424 | N/A | Molecular function: secondary active organic cation transmembrane transporter activity. Biological process: transmembrane transport. | 2.03493412893185e-13 | Dmel\CG42269 |
| DS10_00010970 | Cpr76Bd | Molecular function: structural constituent of chitin-based cuticle. Biological process is unknown. | 2.03167551134315e-13 | Dmel\Cpr76Bd  Cuticular protein 76Bd ([Cpr76Bd](http://flybase.org/search/Cpr76Bd)) encodes a cuticle component involved in adult fly melanization. [Date last reviewed: 2019-03-07] |
| DS10_00002689 | tapdelta | Molecular function: signal sequence binding. Biological process: protein retention in ER lumen. | 4.80003675102179e-13 | Dmel\Tapδ  Translocon-associated protein delta, l(2)k17005 |
| DS10_00006431 | ade5 | Molecular function: phosphoribosylaminoimidazolesuccinocarboxamide synthase activity. Biological process: 'de novo' IMP biosynthetic process; inter-male aggressive behavior; imaginal disc-derived wing morphogenesis. | 5.56241783217551e-13 | TWO: 1) Dmel\CR40801 pseudogene attribute  2)Dmel\Paics  PAICS bifunctional enzyme ([Paics](http://flybase.org/search/Paics)) encodes an essential enzyme in the pathway for de novo synthesis of the purine nucleotide inosine monophosphate (IMP). IMP is the precursor for purine nucleotides required for nucleic acids, energy transfer, cell signaling, and coenzymes. [Date last reviewed: 2019-03-14] |
| DS10_00002210 | N/A | Molecular function: glucuronosyltransferase activity. Biological process: metabolic process. | 5.85861431027844e-13 | Dmel\Ugt49B1  UDP-glycosyltransferase family 49 member B1 |
| DS10_00007600 | N/A | Molecular function: metallopeptidase activity. Biological process: proteolysis. | 6.97308872643523e-13 | Dmel\Nepl11  Neprilysin-like 11 |
| DS10_00009055 | Ubpy | Molecular function: ubiquitin-specific protease activity. Biological process: protein deubiquitination; positive regulation of canonical Wnt receptor signaling pathway; imaginal disc-derived wing margin morphogenesis; positive regulation of smoothened signaling pathway. | 7.23150744148007e-13 | Dmel\Usp8  Ubiquitin specific protease 8 ([Usp8](http://flybase.org/search/Usp8)) encodes a protease involved in protein deubiquitination. It contributes to the integrity of ESCRT sorting machinery and the regulation of Hedgehog and Wingless signaling pathways. [Date last reviewed: 2019-03-21] |
| DS10_00005778 | Aats-asp | Molecular function: aspartate-tRNA ligase activity. Biological process: growth; neurogenesis. | 8.49923410082469e-13 | Dmel\AspRS  Aspartyl-tRNA synthetase ([AspRS](http://flybase.org/search/AspRS)) is an essential gene that is predicted to encode an aspartate-tRNA ligase. It functions as a [Sxl](http://flybase.org/search/Sxl) dosage-sensitive maternal modifier. [Date last reviewed: 2019-08-01] |
| DS10_00011371 | N/A | Molecular function: carbohydrate binding. Biological process is unknown. | 1.6873623532275e-12 | Dmel\CG7763 |
| DS10_00006145 | N/A | Molecular function: argininosuccinate lyase activity. Biological process: arginine biosynthetic process via ornithine. | 2.10265520749758e-12 | Dmel\Argl  Argininosuccinate lyase ([4.3.2.1](https://enzyme.expasy.org/EC/4.3.2.1)) |
| DS10_00013396 | No results | No results;  Top BLAST results: Drosophila suzukii neuropeptide-like 2 (LOC108013988), mRNA | 2.75668171053754e-12 | No results |
| DS10_00001906 | N/A | Molecular function: phosphoglycerate dehydrogenase activity. Biological process: oxidation-reduction process; L-serine biosynthetic process. | 3.14473527822538e-12 | Dmel\CG6287 |
| DS10_00006337 | comt | Molecular function: ATPase activity. Biological process: Golgi organization; regulation of short-term neuronal synaptic plasticity; phagocytosis, engulfment. | 3.18846963681341e-12 | Dmel\comt  comatose ([comt](http://flybase.org/search/comt)) encodes the N-ethylmaleimide-Sensitive Factor 1 protein. It is required for maintenance of neurotransmitter release through disassembly or rearrangement of plasma membrane SNARE complexes following synaptic vesicle fusion. [Date last reviewed: 2019-03-07]  Adenosinetriphosphatase ([3.6.1.3](https://enzyme.expasy.org/EC/3.6.1.3)) |
| DS10_00006011 | Gip | molecular function: hydroxypyruvate isomerase activity. Biological process: glyoxylate metabolic process. | 4.4680823256942e-12 | Dmel\Gip  Hydroxypyruvate isomerase ([5.3.1.22](https://enzyme.expasy.org/EC/5.3.1.22)) |
| DS10_00010487 | kkv | Molecular function: chitin synthase activity. Biological process: trachea morphogenesis; Malpighian tubule morphogenesis; response to wounding; regulation of tube diameter, open tracheal system; terminal branching, open tracheal system; embryonic epithelial tube formation; open tracheal system development; regulation of tube length, open tracheal system; chitin-based embryonic cuticle biosynthetic process; cuticle chitin biosynthetic process. | 4.78724598106116e-12 | Dmel\kkv  krotzkopf verkehrt ([kkv](http://flybase.org/search/kkv)) encodes a membrane-inserted glycosyltransferase family 2 chitin synthase that uses GlcNAc to produce the polysaccharide chitin, which is extruded to the differentiating cuticle and tracheal system (tracheal cuticle, tube diameter regulation and gas filling). [Date last reviewed: 2019-03-14] |
| DS10_00004344 | N/A | Molecular function: 4-hydroxyphenylpyruvate dioxygenase activity. Biological process: oxidation-reduction process; aromatic amino acid family metabolic process. | 5.3060025189256e-12 | Dmel\Hpd |
| DS10_00008639 | N/A | Molecular function is unknown. Biological process is unknown. | 5.37610143907704e-12 | Dmel\CG17726  Type II protein arginine methyltransferase ([2.1.1.320](https://enzyme.expasy.org/EC/2.1.1.320)) |
| DS10_00000408 | N/A | Molecular function is unknown. Biological process: cotranslational protein targeting to membrane. | 5.67827706955924e-12 | Dmel\CG5885 |
| DS10_00006426 | N/A | Molecular function: metalloendopeptidase activity. Biological process: proteolysis. | 5.72861228588263e-12 | Dmel\Nep6  Neprilysin 6 |
| DS10_00000250 | Cyp4ac1 | Molecular function: electron carrier activity. Biological process: insecticide catabolic process; hormone metabolic process. | 6.62126790369395e-12 | Dmel\Cyp4ac1 |
| DS10_00007413 | N/A | Molecular function: methylcrotonoyl-CoA carboxylase activity. Biological process: leucine metabolic process. | 7.67980887110227e-12 | Dmel\Mccc1  Methylcrotonoyl-CoA carboxylase 1 ([Mccc1](http://flybase.org/search/Mccc1)) encodes the biotin-attachment subunit of the 3-methylcrotonyl-CoA carboxylase. It catalyzes the conversion of 3-methylcrotonyl-CoA to 3-methylglutaconyl-CoA, a critical step for leucine and isovaleric acid catabolism. [Date last reviewed: 2019-03-14] |
| DS10_00011200 | N/A | Molecular function: transferase activity, transferring phosphorus-containing groups. Biological process is unknown. | 8.13986643662998e-12 | Dmel\CG31380 |
| DS10_00003962 | N/A | Molecular function: acireductone dioxygenase [iron(II)-requiring] activity. Biological process: oxidation-reduction process. | 8.81559783897659e-12 | Dmel\Adi1  Acireductone dioxygenase 1 ([Adi1](http://flybase.org/search/Adi1)) encodes an enzyme in the methionine salvage pathway (MTA cycle) that is required for fecundity. [Date last reviewed: 2019-03-07] |
| DS10_00004932 | l(1)G0320 | Molecular function: signal sequence binding. Biological process is unknown. | 8.84091383656262e-12 | Dmel\l(1)G0320  lethal (1) G0320 |
| DS10_00011639 | No results | No Results; Top BLAST result:  Drosophila suzukii uncharacterized LOC108011063 (LOC108011063), mRNA  Several other Drosophilids have this, so it seems well-conserved | 1.22411920526284e-11 | No results |
| DS10_00011383 | N/A | Molecular function: transferase activity, transferring phosphorus-containing groups. Biological process is unknown. | 1.31139279582265e-11 | Dmel\CG10514 |
| DS10_00012228 | Tctp | Molecular function: guanyl-nucleotide exchange factor activity; Ras guanyl-nucleotide exchange factor activity; Ras GTPase binding. Biological process: positive regulation of cell size; positive regulation of multicellular organism growth. | 2.68864305467319e-11 | Dmel\Tctp  Translationally controlled tumor protein ([Tctp](http://flybase.org/search/Tctp)) encodes a protein that belongs to the TCTP family. It interacts with the GTPase encoded by [Rheb](http://flybase.org/search/Rheb) to regulate organ growth. It is required for DNA damage response and genome stability. [Date last reviewed: 2019-03-14] |
| DS10_00003614 | N/A | Molecular function: scavenger receptor activity. Biological process: autophagic cell death; salivary gland cell autophagic cell death. | 2.8843576220273e-11 | Dmel\CG3829 |
| DS10_00002659 | Listericin | Molecular function is unknown. Biological process: defense response to Gram-positive bacterium; defense response to Gram-negative bacterium; peptidoglycan recognition protein signaling pathway; positive regulation of peptidoglycan recognition protein signaling pathway. | 3.18138313765267e-11 | Dmel\Listericin  Listericin ([Listericin](http://flybase.org/search/Listericin)) encodes an antibacterial protein whose induction is cooperatively regulated by the product of [PGRP-LE](http://flybase.org/search/PGRP-LE) and the JAK-STAT pathway. [Date last reviewed: 2019-03-14] |
| DS10_00008394 | N/A | Molecular function: metal ion binding. Biological process: L-methionine salvage. | 3.24203173534401e-11 | Dmel\CG11134  Methylthioribulose 1-phosphate dehydratase |
| DS10_00009966 | Tm1 | Molecular function: actin filament binding. Biological process: regulation of lamellipodium assembly; dendrite morphogenesis; pole plasm oskar mRNA localization. | 3.28834561698759e-11 | Tropomyosin 1  Dmel\Tm1 |
| DS10_00003868 | RpS9 | Molecular function: structural constituent of ribosome. Biological process: mitotic spindle elongation; mitotic spindle organization. | 3.70570778102165e-11 | Dmel\RpS9  Ribosomal protein S9 ([RpS9](http://flybase.org/search/RpS9)) encodes a structural constituent of ribosomes. [Date last reviewed: 2019-07-11] |
| DS10_00007564 | N/A | Molecular function: phosphotransferase activity, alcohol group as acceptor. Biological process: carbohydrate metabolic process. | 3.76067158592064e-11 | Dmel\CG11594  D-ribulokinase |
| DS10_00011510 | kar | Molecular function: monocarboxylic acid transmembrane transporter activity. Biological process: ommochrome biosynthetic process. | 3.98949773223149e-11 | Dmel\kar  karmoisin ([kar](http://flybase.org/search/kar)) encodes a protein involved in ommochrome biosynthesis. [Date last reviewed: 2019-09-12] |
| DS10_00002640 | Spn47C | Molecular function is unknown. Biological process: multicellular organism reproduction | 4.81538694765645e-11 | Dmel\Spn47C  Serpin 47C |
| DS10_00010717 | N/A | Molecular function: cation channel activity. Biological process: monovalent inorganic cation transport. | 4.85432230909978e-11 | Dmel\CG33061 |
| DS10_00004912 | N/A | Molecular function: GTP binding. Biological process is unknown. | 5.34740259532908e-11 | Dmel\CG1354  Adenosinetriphosphatase |
| DS10_00000944 | N/A | Molecular function: serine-type endopeptidase activity. Biological process: positive regulation of hemocyte proliferation. | 5.63702278010971e-11 | Dmel\CG4793 |
| DS10_00010604 | N/A | Molecular function: metalloendopeptidase activity. Biological process: proteolysis. | 5.68528921582198e-11 | Dmel\Nepl13  Neprilysin-like 13 |
| DS10_00005038 | Spat | Molecular function: alanine-glyoxylate transaminase activity. Biological process: glyoxylate catabolic process. | 5.74814954609099e-11 | Dmel\Spat  Serine pyruvate aminotransferase ([Spat](http://flybase.org/search/Spat)) encodes an alanine-glyoxylate transaminase involved in glyoxylate catabolism. [Date last reviewed: 2019-09-19] |
| DS10_00000519 | N/A | Molecular function: oxidoreductase activity, acting on the CH-OH group of donors, NAD or NADP as acceptor. Biological process: metabolic process. | 6.05992760236442e-11 | Dmel\CG6012 |
| DS10_00011092 | RpL10Ab | Molecular function: structural constituent of ribosome. Biological process: mitotic spindle elongation; mitotic spindle organization; centrosome duplication. | 6.32602210137763e-11 | Dmel\RpL10Ab  Ribosomal protein L10Ab ([RpL10Ab](http://flybase.org/search/RpL10Ab)) is an essential gene during oogenesis that encodes a ribosomal protein of the large ribosome subunit. [Date last reviewed: 2018-10-25] |
| DS10_00007788 | PGRP-SD | Molecular function: peptidoglycan binding. Biological process: defense response to Gram-positive bacterium. | 6.48829212836914e-11 | PGRP-SD  Peptidoglycan recognition protein SD ([PGRP-SD](http://flybase.org/search/PGRP-SD)) encodes a secreted member of the peptidoglycan recognition protein of from Gram negative bacteria. It is a positive regulator of the Imd pathway by promoting the recognition of peptidoglycan. [Date last reviewed: 2019-03-21] |
| DS10_00011384 | No results | No results; BLAST top reslut: Drosophila suzukii uncharacterized LOC108007920 (LOC108007920), mRNA  ; in several other species this is similar to an uncharacterized protein | 6.46899572175007e-11 | No results |
| DS10_00010040 | Rop | Molecular function: SNARE binding. Biological process: synaptic transmission; response to light stimulus; neurotransmitter secretion; cytokinesis; regulation of pole plasm oskar mRNA localization; secretion by cell. | 6.99254477968744e-11 | Dmel\Rop  Ras opposite |
| DS10_00000939 | Tep1 | Molecular function: endopeptidase inhibitor activity. Biological process: antibacterial humoral response. | 8.96855958686763e-11 | Dmel\Tep1  Thioester-containing protein 1 |
| DS10_00005807 | DptB | Molecular function is unknown. Biological process: humoral immune response; defense response to Gram-positive bacterium. | 1.03309857944096e-10 | Dmel\DptB  Diptericin B ([DptB](http://flybase.org/search/DptB)) encodes an immune inducible antibacterial peptide with activity against Gram-negative bacteria. It is expressed in the fat body during the systemic immune response and in various epithelia. Its expression is regulated at the transcriptional level by the immune deficiency pathway. |
| DS10_00010653 | N/A | Molecular function is unknown. Biological process is unknown. | 1.1802606283775e-10 | Dmel\CG18249 |
| DS10_00008815 | N/A | Molecular function: short-branched-chain-acyl-CoA dehydrogenase activity. Biological process: oxidation-reduction process. | 1.3150857815797e-10 | Dmel\CG3902 |
| DS10_00013334 | No results | No results; top NCBI nucleotide BLAST result: PREDICTED: Drosophila suzukii glutathione S-transferase D4-like (LOC108007823), mRNA | 1.31217162192507e-10 | No results |
| DS10_00000157 | lectin-24Db | Molecular function: mannose binding; fucose binding. Biological process is unknown | 1.44799208668127e-10 | Dmel\lectin-24Db  lectin-24Db ([lectin-24Db](http://flybase.org/search/lectin-24Db)) encodes a protein that interacts specifically with fucose and mannose. [Date last reviewed: 2019-09-19] |
| DS10_00000055 | Spp | Molecular function: aspartic endopeptidase activity, intramembrane cleaving. Biological process: cellular response to unfolded protein; signal peptide processing; open tracheal system development. | 1.6925537083771e-10 | Dmel\Spp  Signal peptide peptidase |
| DS10_00004958 | Cys | Molecular function: cysteine-type endopeptidase inhibitor activity. Biological process: multicellular organism reproduction | 1.72018072023115e-10 | Dmel\Cys  Cystatin-like |
| DS10_00009646 | N/A | Molecular function: N6-(1,2-dicarboxyethyl)AMP AMP-lyase (fumarate-forming) activity. Biological process: purine nucleotide metabolic process. | 1.90109079697404e-10 | Dmel\AdSL  Adenylosuccinate Lyase ([AdSL](http://flybase.org/search/AdSL)) encodes a protein that might be a lyase involved in purine nucleotide metabolic process based on orthology. [Date last reviewed: 2019-03-07] |
| DS10_00007855 | Drsl6 | Molecular function is unknown. Biological process: defense response to fungus. | 2.0820245187904e-10 | Dmel\Drsl6  Drosomycin-like 6 ([Drsl6](http://flybase.org/search/Drsl6)) encodes a secreted peptide with homology to the antifungal peptide encoded by [Drs](http://flybase.org/search/Drs). [Date last reviewed: 2019-03-07] |
| DS10_00002289 | N/A | Molecular function is unknown. Biological process is unknown. | 2.51669732382284e-10 | Dmel\CG16786 |
| DS10_00010756 | Surf4 | Molecular function is unknown. Biological process: lateral inhibition | 2.58074320017413e-10 | Dmel\Surf4  Surfeit 4 |
| DS10_00012740 | N/A | Molecular function: metalloendopeptidase activity. Biological process: proteolysis. | 2.90774791000445e-10 | Dmel\Nepl5  Neprilysin-like 5 |
| DS10_00009645 | N/A | Molecular function: N6-(1,2-dicarboxyethyl)AMP AMP-lyase (fumarate-forming) activity. Biological process: purine nucleotide metabolic process. [THIS IS EXACTLY THE SAME AS A FEW ABOVE??] | 3.19062776173238e-10 | Dmel\AdSL  Adenylosuccinate Lyase ([AdSL](http://flybase.org/search/AdSL)) encodes a protein that might be a lyase involved in purine nucleotide metabolic process based on orthology. [Date last reviewed: 2019-03-07] |
| DS10_00003751 | N/A | Molecular function: isovaleryl-CoA dehydrogenase activity. Biological process: leucine catabolic process. | 3.51540205840755e-10 | Dmel\CG6638  Isovaleryl-CoA dehydrogenase ([1.3.8.4](https://enzyme.expasy.org/EC/1.3.8.4)) |
| DS10_00003730 | ImpE1 | Molecular function is unknown. Biological process: imaginal disc eversion. | 3.59724301467751e-10 | Dmel\ImpE1  cdysone-inducible gene E1 ([ImpE1](http://flybase.org/search/ImpE1)) encodes a protein similar to a low-density lipoprotein (LDL) receptor. It is hypothesized to play a role in the cell rearrangements associated with morphogenesis of the disc epithelium. [ImpE1](http://flybase.org/search/ImpE1) transcription is upregulated by 20-hydroxyecdysone. [Date last reviewed: 2019-03-07] |
| DS10_00003772 | Prm | Molecular function: structural constituent of muscle. Biological process: myofibril assembly; mesoderm development. | 3.5765487467836e-10 | Dmel\Prm  Paramyosin ([Prm](http://flybase.org/search/Prm)) encodes a muscle protein found only in invertebrates. It dimerizes into a coiled-coil that occupies the interior of the thick filament. Its role is thought to be structural, although it can be phosphorylated and this is important for muscle function. [Date last reviewed: 2019-03-14] |
| DS10_00006498 | N/A | Molecular function is unknown. Biological process is unknown. Top BLAST result: PREDICTED: Drosophila suzukii neurogenic locus notch homolog protein 1 (LOC108005481), mRNA | 3.59013841024011e-10 | Dmel\CG9572 |
| DS10_00003798 | pix | Molecular function: ATP binding; ribosomal small subunit binding. Biological process: negative regulation of neuron apoptotic process; translational initiation; cell growth; translation. | 3.73278632231122e-10 | Dmel\pix  pixie ([pix](http://flybase.org/search/pix)) encodes ABCE1, which is an ATPase that functions as a translation recycling factor. It is required for the assembly of complexes involved in translation initiation, and plays a role in the regulation of cellular and organismal growth. [Date last reviewed: 2019-03-14] |
| DS10_00003073 | N/A | Molecular function is unknown. Biological process is unknown. TOP BLAST RESULT: PREDICTED: Drosophila suzukii flocculation protein FLO11 (LOC108009713), transcript variant X3, mRNA; there are several isoforms of this | 3.81176885299959e-10 | Dmel\CG10936 |
| DS10_00008292 | No results | No results; Top BLAST result: PREDICTED: Drosophila suzukii vasotab (LOC108020833), mRNA | 4.34984053062211e-10 | No results |
| DS10_00002842 | N/A | Molecular function: D-aspartate oxidase activity. Biological process: oxidation-reduction process. | 5.40283027837346e-10 | Dmel\CG12338  D-amino-acid oxidase |
| DS10_00000242 | TotM | Molecular function is unknown. Biological process: response to bacterium; defense response to Gram-positive bacterium; cellular response to heat. | 6.09318682024664e-10 | Dmel\TotM  Turandot M ([TotM](http://flybase.org/search/TotM)) encodes a protein that belongs to a class of poorly characterized secreted peptides. Some Turandot genes are expressed in response to stress in the fat body by the JAK-STAT pathway. [Date last reviewed: 2019-03-14] |
| DS10_00006144 | Tsp29Fb | Molecular function is unknown. Biological process is unknown. | 6.75084326561885e-10 | Dmel\Tsp29Fb  Tetraspanin 29Fb |
| DS10_00011764 | Obp83g | Molecular function: odorant binding. Biological process: sensory perception of chemical stimulus. | 7.21435487310578e-10 | Dmel\Obp83g  Odorant-binding protein 83g |
| DS10_00002055 | RpL11 | Molecular function: protein binding; structural constituent of ribosome. Biological process: mitotic spindle elongation; mitotic spindle organization; centrosome duplication. | 8.17664044350065e-10 | Dmel\RpL11  Ribosomal protein L11 |
| DS10_00001525 | No results | No results; top Nucleotide BLAST result: PREDICTED: Drosophila suzukii slowpoke-binding protein (LOC108015014), transcript variant X3, mRNA. Top Protein BLAST result: PREDICTED: serine-rich adhesin for platelets isoform X3 [Drosophila suzukii] | 8.76065020199881e-10 | No results |
| DS10_00003263 | Ef1beta | Molecular function: translation elongation factor activity; translation release factor activity. Biological process: translational elongation. | 8.80267332810507e-10 | Dmel\eEF1β  eukaryotic translation elongation factor 1 beta |
| DS10_00012308 | Ef1gamma | Molecular function: translation elongation factor activity. Biological process: autophagic cell death; salivary gland cell autophagic cell death. | 9.03124249540135e-10 | Dmel\eEF1γ  eukaryotic translation elongation factor 1 gamma ([eEF1γ](http://flybase.org/search/eEF1%CE%B3)) encodes a protein that associates with other EF1 subunits (α/β/δ). It regulates organelle movement along microtubules. It binds tubulin and the protein kinase encoded by [Doa](http://flybase.org/search/Doa), which in turns phosphorylates the product of [eEF1γ](http://flybase.org/search/eEF1%CE%B3). [Date last reviewed: 2019-03-07] |
| DS10_00005017 | RpL17 | Molecular function: structural constituent of ribosome. Biological process: mitotic spindle elongation; mitotic spindle organization; centrosome duplication. | 9.19946616320083e-10 | Dmel\RpL17  Ribosomal protein L17 |
| DS10_00005962 | cyr | Molecular function is unknown. Biological process: epithelial cell morphogenesis. | 9.26248880223595e-10 | Dmel\cyr  cypher |
| DS10_00004161 | N/A | Molecular function: aldose 1-epimerase activity. Biological process: hexose metabolic process. | 9.92662710054965e-10 | Dmel\CG10467  Aldose 1-epimerase |
| DS10_00000452 | eEF1delta | Molecular function: translation elongation factor activity. Biological process: translational elongation. | 1.20595752026146e-09 | Dmel\eEF1δ  eukaryotic translation elongation factor 1 delta |
| DS10_00010605 | N/A | Molecular function: metalloendopeptidase activity. Biological process: compound eye morphogenesis. | 1.22763178584275e-09 | Dmel\Nepl15  Neprilysin-like 15 |
| DS10_00006885 | v | Molecular function: tryptophan 2,3-dioxygenase activity. Biological process: ommochrome biosynthetic process. |  | Dmel\v  Tryptophan 2,3-dioxygenase  vermilion |
| DS10_00004718 | N/A | Molecular function is unknown. Biological process is unknown. Top Nucleotide BLAST result: PREDICTED: Drosophila suzukii zinc finger protein 853 (LOC108015156), mRNA;  Protein BLAST: PREDICTED: zinc finger protein 853 [Drosophila suzukii] | 1.60104345354576e-09 | Dmel\CG11380 |
| DS10_00013527 | Amyrel | Molecular function: oligo-1,6-glucosidase activity; alpha-amylase activity. Biological process: carbohydrate metabolic process. | 1.65653307427303e-09 | Dmel\Amyrel  Amyrel ([Amyrel](http://flybase.org/search/Amyrel)) encodes an amylolytic activity protein that is expressed mainly in larval midgut. [Date last reviewed: 2018-10-04] |
| DS10_00001512 | N/A | Molecular function: retinal binding. Biological process: transport. | 1.74705411960861e-09 | Dmel\CG5958 |
| DS10_00001575 | Aldh | Molecular function: aldehyde dehydrogenase (NAD) activity; acetaldehyde dehydrogenase (acetylating) activity. Biological process: response to ethanol; acetaldehyde metabolic process. | 1.74165230829486e-09 | Dmel\Aldh  Aldehyde dehydrogenase ([Aldh](http://flybase.org/search/Aldh)) encodes an NAD[+] dependent mitochondrial aldehyde dehydrogenase. Its functions include detoxifying endogenous aldehydes generated by lipid peroxidation, and detoxifying acetaldehyde derived from dietary ethanol. [Date last reviewed: 2019-03-07] |
| DS10_00009592 | RpL4 | Molecular function: structural constituent of ribosome. Biological process: centrosome duplication. | 1.76030987614712e-09 | Dmel\RpL4  Ribosomal protein L4 |
| DS10_00009895 | No results | No results; Top nucleotide BLAST result: PREDICTED: Drosophila suzukii heparan sulfate 2-O-sulfotransferase pipe (LOC108020433), partial mRNA Top protein BLAST result: blast:Heparan sulfate 2-O-sulfotransferase pipe [Drosophila guanche] | 1.7491661537042e-09 | No results |
| DS10_00004468 | N/A | Molecular function: ATPase activity, coupled; motor activity. Biological process: microtubule-based movement | 2.02831586421294e-09 | Dmel\CG13930 |
| DS10_00010851 | Dbp73D | Molecular function: ATP-dependent RNA helicase activity. Biological process: neurogenesis. | 2.1846068570482e-09 | Dmel\Dbp73D  Dead box protein 73D |
| DS10_00010707 | fax | Molecular function is unknown. Biological process: axonogenesis; neurogenesis | 2.23091230881678e-09 | Dmel\fax  failed axon connections |
| DS10_00010808 | N/A | Molecular function: UDP-galactose transmembrane transporter activity. Biological process: transmembrane transport. | 2.29992748073955e-09 | Dmel\meigo  medial glomeruli ([meigo](http://flybase.org/search/meigo)) encodes an evolutionarily conserved, multi-membrane pass protein that is mainly localized at the endoplasmic reticulum. [meigo](http://flybase.org/search/meigo) genetically interacts with [Ephrin](http://flybase.org/search/Ephrin) in dendrite targeting of olfactory projection neurons. [Date last reviewed: 2019-03-14] |
| DS10_00009397 | RpS20 | Molecular function: structural constituent of ribosome. Biological process: translation. | 2.42562595782519e-09 | Dmel\RpS20  Ribosomal protein S20 |
| DS10_00012957  [POTENTIAL DOUBLE GENE SEE ABOVE] | RpS20 | Molecular function: structural constituent of ribosome. Biological process: translation. | 2.469622954786e-09 | Dmel\RpS20  Ribosomal protein S20 |
| DS10_00011509 | N/A | Molecular function: glycine N-methyltransferase activity. Biological process is unknown. | 2.60137858404862e-09 | Dmel\Gnmt  Glycine N-methyltransferase ([Gnmt](http://flybase.org/search/Gnmt)) encodes an enzyme that catalyzes the methylation reaction of glycine to generate sarcosine (N-methylglycine). It is abundantly expressed in the fat body and controls the amount of methyl donor S-adenosylmethionine. [Date last reviewed: 2019-03-07] |
| DS10_00003612 | Phk-3 | Molecular function: diacylglycerol binding; protein serine/threonine kinase activity. Biological process: response to bacterium; metamorphosis. | 2.66072016748725e-09 | Dmel\Phk-3  Pherokine 3 |
| DS10_00000482 | lectin-28C | Molecular function: galactose binding; carbohydrate binding. Biological process is unknown. | 2.75017941970771e-09 | Dmel\lectin-28C |
| DS10_00004748 | Sec61gamma | Molecular function: protein transporter activity. Biological process: negative regulation of autophagy. | 2.77134507731322e-09 | Dmel\Sec61γ  Sec61 γ subunit  Mitochondrial protein-transporting ATPase ([7.4.2.3](https://enzyme.expasy.org/EC/7.4.2.3))  Chloroplast protein-transporting ATPase ([7.4.2.4](https://enzyme.expasy.org/EC/7.4.2.4)) |
| DS10_00004320 | cmpy | Molecular function: protein binding. Biological process: negative regulation of BMP signaling pathway by negative regulation of BMP secretion; negative regulation of BMP signaling pathway; neuromuscular junction development. | 2.85736302830712e-09 | Dmel\cmpy  crimpy |
| DS10_00009057 | N/A | Molecular function is unknown. Biological process is unknown. Top Nucleotide BLAST result: PREDICTED: Drosophila suzukii leucine-rich repeat-containing G-protein coupled receptor 5 (LOC108018377), mRNA Top PROTEIN BLAST result: PREDICTED: leucine-rich repeat-containing G-protein coupled receptor 5 [Drosophila suzukii] | 2.84753473680043e-09 | Dmel\cDIP  Common Dpr-interacting protein |
| DS10_00004860 | Cyp6v1 | Molecular function: electron carrier activity. Biological process: oxidation-reduction process. | 2.92884267902518e-09 | Dmel\Cyp6v1 |
| DS10_00001395 | Sec61alpha | Molecular function: protein transporter activity. Biological process: dorsal closure; neuron projection morphogenesis; positive regulation of cell death; cuticle development; head involution; cell death; negative regulation of autophagy. | 2.98146920224779e-09 | Dmel\Sec61α  Sec61 α subunit ([Sec61α](http://flybase.org/search/Sec61%CE%B1)) encodes a subunit of the the translocon, a protein-conducting channel that mediates the co-translational transport of nascent polypeptides into the endoplasmic reticulum. It is a subunit of the Sec61 heterotrimer that forms the core of translocon channel. [Date last reviewed: 2019-03-14] |
| DS10_00009232 | RpL35A | Molecular function: structural constituent of ribosome. Biological process: translation. | 2.97841855491474e-09 | Dmel\RpL35A  Ribosomal protein L35A |
| DS10_00011471 | Adgf-A | Molecular function: growth factor activity; adenosine deaminase activity. Biological process: cell proliferation; hemocyte differentiation. | 3.17907783021914e-09 | Dmel\Adgf-A  Adenosine deaminase-related growth factor A ([Adgf-A](http://flybase.org/search/Adgf-A)) encodes an extracellular deaminase that regulates the level of extracellular adenosine by converting adenosine to inosine. This activity is important for regulation of systemic metabolism, especially during stress and infection. [Date last reviewed: 2019-03-07] |
| DS10_00011761 | Gasp | Molecular function: chitin binding; structural constituent of peritrophic membrane. Biological process: chitin metabolic process | 3.39484920005417e-09 | Dmel\Gasp  Gasp ([Gasp](http://flybase.org/search/Gasp)) encodes a protein involved in cuticle biosynthesis during embryonic tracheal system development. [Date last reviewed: 2019-09-19] |
| DS10_00012294 | ade2 | Molecular function: phosphoribosylformylglycinamidine synthase activity. Biological process: 'de novo' IMP biosynthetic process. | 3.48204785402014e-09 | Dmel\Pfas  Phosphoribosylformylglycinamidine synthase ([Pfas](http://flybase.org/search/Pfas)) encodes a phosphoribosylformylglycinamidine synthase, which is an essential enzyme in the pathway for de novo synthesis of the purine nucleotide inosine monophosphate (IMP). IMP is the precursor for purine nucleotides required for nucleic acids, energy transfer, cell signaling, and coenzymes. [Date last reviewed: 2019-03-14] |
| DS10_00000313 | N/A | Molecular function: nucleotide binding; oxidoreductase activity. Biological process is unknown. | 4.09697802405746e-09 | Dmel\CG13280 |
| DS10_00001601 | Apoltp | Molecular function: lipid transporter activity. Biological process: acylglycerol transport; sterol transport. | 4.16170064361603e-09 | Dmel\Apoltp  Apolipoprotein lipid transfer particle ([Apoltp](http://flybase.org/search/Apoltp)) encodes a apolipoprotein of the ApoB family. It is assembled with lipids to form high density, low abundance lipoproteins named lipid transfer particles (LTP). LTP catalyzes the transfer of lipids from the gut to circulating lipophorin and from lipophorin to peripheral tissues, such as imaginal discs and ovaries. [Date last reviewed: 2019-03-07] |
| DS10_00003620 | gol | Molecular function: zinc ion binding. Biological process: regulation of transcription, DNA-dependent; mesoderm development. | 4.37209197741265e-09 | Dmel\gol  goliath ([gol](http://flybase.org/search/gol)) encodes an E3 ubiquitin ligase, whose cellular substrates are unknown. During embyrogenesis [gol](http://flybase.org/search/gol) is expressed in fusion-competence myoblasts of the somatic and visceral mesoderm. [Date last reviewed: 2019-03-07] |
| DS10_00005431 | N/A | Molecular function is unknown. Biological process is unknown Top NUCLEOTIDE BLAST result: PREDICTED: Drosophila suzukii 28 kDa heat- and acid-stable phosphoprotein (LOC108016631), mRNA Top PROTEIN BLAST result: PREDICTED: 28 kDa heat- and acid-stable phosphoprotein [Drosophila suzukii] . | 4.38161242887115e-09 | Dmel\CG11444 |
| DS10_00010712 | Cpr72Ec | Molecular function: structural constituent of chitin-based cuticle. Biological process is unknown | 4.41424280781943e-09 | Dmel\Cpr72Ec  Cuticular protein 72Ec |
| DS10_00008766 | sxe2 | Molecular function: phospholipase activity. Biological process: lipid catabolic process. | 4.68782041741355e-09 | Dmel\sxe2  sex-specific enzyme 2 |
| DS10_00007854 | Drsl4 | Molecular function is unknown. Biological process: defense response to fungus. | 5.14094082183924e-09 | Dmel\Drsl4  Drosomycin-like 4 ([Drsl4](http://flybase.org/search/Drsl4)) encodes a secreted peptide with homology to the antifungal peptide encoded by [Drs](http://flybase.org/search/Drs). [Date last reviewed: 2019-03-07] |
| DS10_00007928 | RpLP0 | Molecular function: structural constituent of ribosome. Biological process: translation | 6.22569362366458e-09 | Dmel\RpLP0  Ribosomal protein LP0 |
| DS10_00013296 | N/A | Molecular function is unknown. Biological process is unknown. TOP nucleotide BLAST result: PREDICTED: Drosophila suzukii uncharacterized LOC108007321 (LOC108007321), mRNA Top protein BLAST result: PREDICTED: cuticle protein 65 [Drosophila eugracilis] | 6.3823622182447e-09 | Dmel\CG4962 |
| DS10_00009930 | Mlc1 | Molecular function: microfilament motor activity. Biological process: mesoderm development | 6.56579587123765e-09 | Dmel\Mlc1  Myosin alkali light chain 1 |
| DS10_00005305 | N/A | Molecular function: methylmalonate-semialdehyde dehydrogenase (acylating) activity. Biological process: valine metabolic process; pyrimidine nucleobase metabolic process. | 7.00159599611309e-09 | Dmel\CG17896  Malonate-semialdehyde dehydrogenase (acetylating) ([1.2.1.18](https://enzyme.expasy.org/EC/1.2.1.18))  Methylmalonate-semialdehyde dehydrogenase (CoA acylating) |
| DS10_00010809 | N/A | Molecular function is unknown. Biological process is unknown. Top nucleotide BLAST: PREDICTED: Drosophila suzukii leucine-rich repeat transmembrane neuronal protein 2-like (LOC108014408), mRNA Top Protein BLAST: PREDICTED: leucine-rich repeat transmembrane neuronal protein 2-like [Drosophila suzukii] | 6.96485084126739e-09 | Dmel\cDIP  Common Dpr-interacting protein |
| DS10_00008814 | N/A | Molecular function: short-branched-chain-acyl-CoA dehydrogenase activity. Biological process: oxidation-reduction process. | 7.0733404202174e-09 | Dmel\CG3902 |
| DS10_00005253 | Pgd | Molecular function: phosphogluconate dehydrogenase (decarboxylating) activity. Biological process: pentose-phosphate shunt. | 7.28379514774723e-09 | Dmel\Pgd  Phosphogluconate dehydrogenase ([Pgd](http://flybase.org/search/Pgd)) encodes a cytosolic enzyme that catalyzes the oxidation of 6-phosphogluconate to ribulose 5-phosphate, with the concomitant reduction of NADP[+] to NADPH. The products of [Pgd](http://flybase.org/search/Pgd) and [Zw](http://flybase.org/search/Zw) are the two NADP[+] reducing enzymes in the pentose phosphate pathway. [Date last reviewed: 2019-03-14] |
| DS10_00000986 | Hf | Molecular function is unknown. Biological process: innate immune response. | 7.52327816187413e-09 | Dmel\Hf  Helical Factor |
| DS10_00003540 | N/A | Molecular function is unknown. Biological process is unknown. TOp Nucleotide BLAST result: PREDICTED: Drosophila suzukii uncharacterized LOC108009954 (LOC108009954), mRNA Top Proteing BLAST result: PREDICTED: uncharacterized protein LOC108009954 [Drosophila suzukii] | 7.61433065978142e-09 | Dmel\CG11400 |
| DS10_00002423 | N/A | Molecular function is unknown. Biological process: regulation of transcription, DNA-dependent. | 7.68658193569152e-09 | Dmel\reb  rebuf ([reb](http://flybase.org/search/reb)) and the related [exp](http://flybase.org/search/exp) encode proteins that belong to the Smad/FHA superfamily and have interchangeable functions. Both are required for the process of chitin deposition, together with the chitin synthase encoded by [kkv](http://flybase.org/search/kkv). [Date last reviewed: 2019-03-14] |
| DS10_00011027 | N/A | Molecular function: metalloendopeptidase activity. Biological process: proteolysis. | 7.71424007956136e-09 | Dmel\Nepl21  Neprilysin-like 21 |
| DS10_00003495 | N/A | Molecular function is unknown. Biological process is unknown Top Nucleotide BLAST result: PREDICTED: Drosophila suzukii ovochymase-2 (LOC108009588), mRNA Top Protein BLAST result: PREDICTED: ovochymase-2 [Drosophila suzukii] | 8.08991560715428e-09 | Dmel\CG43742 |
| DS10_00008722 | No results | No results; Top nucleotide BLAST results: PREDICTED: Drosophila suzukii tropomyosin-1, isoforms 33/34 (LOC108011512), mRNA; top protein BLAST result: PREDICTED: golgin-84 isoform X6 [Drosophila biarmipes] | 8.18510245730199e-09 | No results |
| DS10_00009036 | Dhc93AB | Molecular function: ATPase activity, coupled; motor activity. Biological process: sensory perception of sound. | 9.03802885253012e-09 | Dmel\Dhc93AB  Dynein heavy chain at 93AB ([Dhc93AB](http://flybase.org/search/Dhc93AB)) encodes a motor protein involved in hearing. [Date last reviewed: 2019-08-01] Minus-end-directed kinesin ATPase |
| DS10_00002493 | RpS23 | Molecular function: structural constituent of ribosome. Biological process: centrosome organization; centrosome duplication. | 9.67371121262244e-09 | Dmel\RpS23  Ribosomal protein S23 |
| DS10_00007026 | RpS6 | Molecular function: structural constituent of ribosome. Biological process: mitotic spindle elongation; mitotic spindle organization; centrosome duplication. | 9.60795824256412e-09 | Dmel\RpS6  Ribosomal protein S6 ([RpS6](http://flybase.org/search/RpS6)) encodes a key component of the small (40S) ribosomal subunit. [Date last reviewed: 2019-02-28] |
| DS10_00010876 | RpS12 | Molecular function: structural constituent of ribosome. Biological process: translation. | 9.64398920420448e-09 | Multiple results, see April 30, 2020; ribosomal protein |
| DS10_00006029 | Peritrophin-A | Molecular function: chitin binding; structural constituent of peritrophic membrane. Biological process: multicellular organism reproduction. | 1.03973177141566e-08 | Dmel\Peritrophin-A |
| DS10_00005486 | Ca-P60A | Molecular function: protein binding. Biological process: neuromuscular synaptic transmission; cellular calcium ion homeostasis; stabilization of membrane potential; regulation of sequestering of calcium ion; flight behavior. | 1.04721610355617e-08 | Dmel\SERCA  Sarco/endoplasmic reticulum Ca(2+)-ATPase ([SERCA](http://flybase.org/search/SERCA)) encodes an endoplasmic reticulum (ER) calcium pump with roles in ER calcium homeostasis and lipid storage  P-type H(+)-exporting transporter ([7.1.2.1](https://enzyme.expasy.org/EC/7.1.2.1))  P-type Ca(2+) transporter |
| DS10_00007507 | N/A | Molecular function: calcium, potassium:sodium antiporter activity. Biological process: transmembrane transport. | 1.07454074964044e-08 | Multiple results, see April 30, 2020 |
| DS10_00005550 | N/A | Molecular function: glucose transmembrane transporter activity. Biological process: transmembrane transport. | 1.18651356948907e-08 | Dmel\CG8249 |
| DS10_00008637 | RpL3 | Molecular function: structural constituent of ribosome. Biological process: centrosome organization; mitotic spindle elongation; mitotic spindle organization; centrosome duplication. | 1.25765296280208e-08 | Dmel\RpL3  Ribosomal protein L3 |
| DS10_00006893 | N/A | Molecular function is unknown. Biological process is unknown. Top Nucleotide BLAST result: PREDICTED: Drosophila suzukii uncharacterized protein CG1552 (LOC108021828), transcript variant X2, mRNA; Top PROTEIN BLAST result: PREDICTED: uncharacterized protein CG1552 isoform X2 [Drosophila suzukii] | 1.27377175250342e-08 | Dmel\CG1552 |
| DS10_00007035 | RpS14a | Molecular function: structural constituent of ribosome. Biological process: translation. | 1.31249711147633e-08 | Dmel\RpS14a  Ribosomal protein S14a |
| DS10_00004381 | dos | Molecular function: SH2 domain binding; protein binding. Biological process: regulation of Ras protein signal transduction; antimicrobial humoral response. | 1.50413609943671e-08 | Dmel\dos  daughter of sevenless |
| DS10_00007341 | Mfe2 | Molecular function: enoyl-CoA hydratase activity; 3R-hydroxyacyl-CoA dehydratase activity; protein homodimerization activity. Biological process: fatty acid beta-oxidation using acyl-CoA oxidase. | 1.51033271632685e-08 | Dmel\Mfe2  peroxisomal Multifunctional enzyme type 2 ([Mfe2](http://flybase.org/search/Mfe2)) encodes a multifunctional protein harboring two separate enzyme activities: 2-enoyl-CoA hydratase 2 and 3-hydroxyacyl-CoA dehydrogenase. They function in the peroxisomal fatty acid breakdown spiral of beta-oxidation utilizing the (R)-isomer of the CoA-containing substrate. [Date last reviewed: 2019-03-14] |
| DS10_00013055 | N/A | Molecular function is unknown. Biological process is unknown. Top Nucleotide Blast result: PREDICTED: Drosophila suzukii uncharacterized LOC108020185 (LOC108020185), mRNA Top Preoteing BLAST result: PREDICTED: uncharacterized protein LOC108020185 [Drosophila suzukii] | 1.5137597153753e-08 | Dmel\CG12581 |
| DS10_00002184 | TM4SF | Molecular function is unknown. Biological process is unknown. | 1.5862571198369e-08 | Dmel\TM4SF  Transmembrane 4 superfamily |
| DS10_00004676 | RpL35 | Molecular function: structural constituent of ribosome. Biological process: mitotic spindle elongation; mitotic spindle organization; centrosome duplication. | 1.68492803672414e-08 | Dmel\RpL35  Ribosomal protein L35 |
| DS10_00003562 | N/A | Molecular function: S-methyl-5-thioadenosine phosphorylase activity. Biological process: phosphorylation. | 1.7007663397458e-08 | Dmel\Mtap  Methylthioadenosine phosphorylase ([Mtap](http://flybase.org/search/Mtap)) encodes an enzyme that plays a key role in the methionine salvage pathway and in the production of endogenous cellular adenine. [Date last reviewed: 2018-10-18] |
| DS10_00012358 | N/A | Molecular function is unknown. Biological process is unknown. Top NUCLEOTIDE BLAST RESULT: PREDICTED: Drosophila suzukii uncharacterized LOC108015619 (LOC108015619), mRNA Top PROTEIN BLAST result: PREDICTED: uncharacterized protein LOC108015619 [Drosophila suzukii] | 1.82511432351173e-08 | Dmel\CG34253 |
| DS10_00009621 | SpdS | Molecular function: spermidine synthase activity. Biological process: spermidine biosynthetic process. | 1.90612442741029e-08 | Dmel\SpdS |
| DS10_00013331 | No results | No results. Top NUCLEOTIDE BLAST result: PREDICTED: Drosophila suzukii glutathione S-transferase D2-like (LOC108007773), mRNA Top Protein blast result: PREDICTED: glutathione S-transferase D2-like [Drosophila suzukii] | 2.06087617061533e-08 | No results |
| DS10_00003695 | N/A | Molecular function: chitin binding. Biological process: chitin metabolic process. | 2.12516670553961e-08 | Dmel\CG13676 |
| DS10_00005254 | N/A | Molecular function: UDP-N-acetylmuramate dehydrogenase activity; flavin adenine dinucleotide binding. Biological process: oxidation-reduction process. | 2.13913347487572e-08 | Dmel\D2hgdh  D-2-hydroxyglutaric acid dehydrogenase ([D2hgdh](http://flybase.org/search/D2hgdh)) encodes an enzyme involved in oxidation-reduction metabolism. [Date last reviewed: 2019-09-19] |
| DS10_00011150 | SsRbeta | Molecular function: signal sequence binding. Biological process: protein retention in ER lumen. | 2.24844148002931e-08 | Dmel\SsRβ  Signal sequence receptor β |
| DS10_00005802 | Jheh2 | Molecular function: epoxide hydrolase activity. Biological process: juvenile hormone catabolic process. | 2.30438441350939e-08 | Dmel\Jheh2  Juvenile hormone epoxide hydrolase 2 ([Jheh2](http://flybase.org/search/Jheh2)) encodes an enzyme that hydrolyzes cis-stilbene oxide. [Jheh2](http://flybase.org/search/Jheh2) expression changes are associated with increased tolerance to oxidative stress. [Date last reviewed: 2019-03-07] |
| DS10_00004924 | Aats-lys | Molecular function: lysine-tRNA ligase activity. Biological process: lysyl-tRNA aminoacylation | 2.76367397379709e-08 | Dmel\LysRS  Lysyl-tRNA synthetase |
| DS10_00010849 | n/a | Molecular function is unknown. Biological process is unknown. Top NUCLEOTIDE BLAST result: PREDICTED: Drosophila suzukii uncharacterized LOC108006677 (LOC108006677), mRNA Top PROTEIN BLAST result: PREDICTED: uncharacterized protein LOC108006677 [Drosophila suzukii] | 2.85028027978666e-08 | Dmel\CG13026 |
| DS10_00011233 | Ela | Molecular function is unknown. Biological process: response to nicotine. | 2.96371105808442e-08 | Dmel\Elal  Elastin-like |
| DS10_00011970 | N/A | Molecular function: 2 iron, 2 sulfur cluster binding; iron ion binding; electron carrier activity; oxidoreductase activity, acting on CH-OH group of donors; flavin adenine dinucleotide binding. Biological process: oxidation-reduction process. | 3.0513619249797e-08 | Dmel\AOX2  Aldehyde oxidase 2 ([AOX2](http://flybase.org/search/AOX2)) encodes a pyridoxal oxidase involved in pyridoxal metabolism. [Date last reviewed: 2019-09-19] |
| DS10_00010254 | N/A | Molecular function is unknown. Biological process is unknown. Nucleotide BLAST result: PREDICTED: Drosophila suzukii protein tramtrack, alpha isoform (LOC108020484), mRNA Protein BLAST: PREDICTED: protein tramtrack, alpha isoform [Drosophila suzukii] | 3.0821809051258e-08 | Dmel\CG34376 |
| DS10_00002585 | N/A | Molecular function is unknown. Biological process is unknown. Top Nucleotide BLAST result: PREDICTED: Drosophila suzukii proteasome-associated protein ECM29 homolog (LOC108009228), mRNA Proteing BLAST: PREDICTED: proteasome-associated protein ECM29 homolog [Drosophila suzukii] | 3.23154804142867e-08 | Dmel\CG8858 |
| DS10_00004798 | Mec2 | Molecular function: protein binding. Biological process is unknown. | 3.26952164334755e-08 | Dmel\Mec2 |
| DS10_00011607 | pug | Molecular function: methylenetetrahydrofolate dehydrogenase (NADP+) activity; formate-tetrahydrofolate ligase activity; methenyltetrahydrofolate cyclohydrolase activity. Biological process: oxidation-reduction process; folic acid-containing compound biosynthetic process. | 3.46720972058845e-08 | Dmel\pug  pugilist ([pug](http://flybase.org/search/pug)) encodes the trifunctional enzyme methylenetetrahydrofolate dehydrogenase involved in the pigmentation of pteridines and ommochromes. [Date last reviewed: 2019-09-12] |
| DS10_00003095 | N/A | Molecular function: sarcosine dehydrogenase activity. Biological process: oxidation-reduction process; glycine catabolic process. | 3.53980265180582e-08 | Dmel\Sardh  Sarcosine dehydrogenase ([1.5.8.3](https://enzyme.expasy.org/EC/1.5.8.3)) |
| DS10_00000232 | msl-2 | Molecular function: chromatin binding; DNA binding. Biological process: dosage compensation. | 3.59331319060997e-08 | Dmel\msl-2  male-specific lethal 2 ([msl-2](http://flybase.org/search/msl-2)) encodes the key male-specific subunit of the male-specific-lethal dosage compensation complex. It induces or stabilizes all other components. Homozygous mutant males die, while [msl-2](http://flybase.org/search/msl-2) ectopic expression kills females. [Date last reviewed: 2019-03-14] |
| DS10_00007093 | N/A | Molecular function: gamma-aminobutyric acid:sodium symporter activity. Biological process: neurotransmitter transport. | 3.59568684069348e-08 | Dmel\Gat  GABA transporter ([Gat](http://flybase.org/search/Gat)) encodes a protein belonging to the SLC family that is expressed primarily in astrocytes, where it is thought to be required for uptake of GABA and other small molecules. It likely plays an important role in setting the excitatory/inhibitory balance of the CNS. [Date last reviewed: 2019-03-07] |
| DS10_00011463 | Npc2g | Molecular function: sterol binding. Biological process: hemolymph coagulation; mesoderm development. | 3.73120526476175e-08 | Dmel\Npc2g  Niemann-Pick type C-2g |
| DS10_00005606 | N/A | Molecular function is unknown. Biological process is unknown. Top Nucleotide BLAST result: PREDICTED: Drosophila suzukii uncharacterized LOC108018145 (LOC108018145), mRNA Top Protein BLAST result: PREDICTED: uncharacterized protein LOC108018145 [Drosophila suzukii] | 3.84024706497924e-08 | Dmel\CG34215 |
| DS10_00010892 | RpL13A | Molecular function: structural constituent of ribosome. Biological process: Notch signaling pathway; chaeta morphogenesis; centrosome duplication. | 4.06582693051471e-08 | Dmel\RpL13A  Ribosomal protein L13A ([RpL13A](http://flybase.org/search/RpL13A)) encodes a component of the large ribosomal subunit, and thus involved in mRNA translation. Mutations in genes encoding ribosomal proteins affect fertility, viability and other Drosophila phenotypes, described as Minute. [Date last reviewed: 2019-03-14] |
| DS10_00002757 | Xbp1 | Molecular function: sequence-specific DNA binding transcription factor activity; protein homodimerization activity. Biological process: chaeta development; endoplasmic reticulum unfolded protein response; positive regulation of transcription from RNA polymerase II promoter involved in unfolded protein response; wing disc development; lateral inhibition; response to endoplasmic reticulum stress. | 4.55050069821446e-08 | Dmel\Xbp1  X box binding protein-1 ([Xbp1](http://flybase.org/search/Xbp1)) encodes a transcription factor that mediates the unfolded protein response. [Xbp1](http://flybase.org/search/Xbp1) mRNA undergoes splicing after being cleaved by the product of [Ire1](http://flybase.org/search/Ire1), inducing the expression of ER quality control transcripts. [Xbp1](http://flybase.org/search/Xbp1) mutants fail to develop beyond the 2nd instar larval stage, indicative of a requirement to resolve inherent ER stress during normal development. [Date last reviewed: 2019-03-21] |
| DS10_00009562 | N/A | Molecular function: procollagen-proline 4-dioxygenase activity. Biological process: peptidyl-proline hydroxylation to 4-hydroxy-L-proline. | 4.53694343995941e-08 | Dmel\CG31016 |
| DS10_00013335 | No results | No results  Top Nucleotide BLAST result: PREDICTED: Drosophila suzukii glutathione S-transferase 1-1 (LOC108007820), partial mRNA Top Protein BLAST result: PREDICTED: glutathione S-transferase 1-1, partial [Drosophila suzukii] | 4.68514589928511e-08 | No results |
| DS10_00012601 | ade2 | Molecular function: phosphoribosylformylglycinamidine synthase activity. Biological process: 'de novo' IMP biosynthetic process. | 4.87637787056182e-08 | Dmel\Pfas  Phosphoribosylformylglycinamidine synthase ([Pfas](http://flybase.org/search/Pfas)) encodes a phosphoribosylformylglycinamidine synthase, which is an essential enzyme in the pathway for de novo synthesis of the purine nucleotide inosine monophosphate (IMP). IMP is the precursor for purine nucleotides required for nucleic acids, energy transfer, cell signaling, and coenzymes. [Date last reviewed: 2019-03-14] |
| DS10_00001840 | N/A | Molecular function: aminomethyltransferase activity. Biological process: glycine catabolic process. | 4.93565704769489e-08 | Dmel\CG6415  Aminomethyltransferase |
| DS10_00004759 | RpS10b | Molecular function: structural constituent of ribosome. Biological process: translation. | 4.96972568710853e-08 | Ribosomal protein S10b |
| DS10_00009965 | Tm2 | Molecular function: actin binding. Biological process: heart development. | 5.13428632611299e-08 | Dmel\Tm2  Tropomyosin 2 |
| DS10_00006154 | No results | No results Top Nucleotide BLAST result: PREDICTED: Drosophila suzukii guanine nucleotide-binding protein subunit beta-like protein (LOC108019065), mRNA Top Protein BLAST result: receptor of activated protein kinase C 1, isoform C [Drosophila melanogaster] | 5.18000622012284e-08 | No results |
| DS10_00002234 | Fkbp13 | Molecular function: FK506 binding; peptidyl-prolyl cis-trans isomerase activity. Biological process: inter-male aggressive behavior; muscle cell homeostasis. | 5.4663152262246e-08 | Dmel\Fkbp14  FK506-binding protein 14 ([Fkbp14](http://flybase.org/search/Fkbp14)) encodes an ER resident protein and a member of the large FKBP family of immunophilins. It plays an essential role in development, including stabilizing the protein encoded by [Psn](http://flybase.org/search/Psn) in the ER. [Fkbp14](http://flybase.org/search/Fkbp14) mutants genetically interact with components of the Notch pathway. [Date last reviewed: 2018-09-13] |
| DS10_00001360 | Ef2b | Molecular function: translation elongation factor activity. Biological process: mitotic spindle elongation; mitotic spindle organization. | 5.59125067218797e-08 | Dmel\eEF2  eukaryotic translation elongation factor 2 |
| DS10_00004705 | N/A | Molecular function is unknown. Biological process is unknown. Top nucleotide BLAST result  PREDICTED: Drosophila suzukii 27 kDa hemolymph protein (LOC108015198), mRNA Top Protein BLAST result: PREDICTED: 27 kDa hemolymph protein [Drosophila suzukii] | 5.83342932483933e-08 | Dmel\CG14629 |
| DS10_00006694 | Odc1 | molecular function: ornithine decarboxylase activity. Biological process: polyamine biosynthetic process. | 5.97273525236301e-08 | Dmel\Odc1  Ornithine decarboxylase 1 |
| DS10_00007909 | scramb2 | Molecular function: phospholipid scramblase activity. Biological process: synaptic transmission. | 6.13893127485516e-08 | Dmel\scramb2  scramblase 2 ([scramb2](http://flybase.org/search/scramb2)) encodes a protein that, together with the product of [scramb1](http://flybase.org/search/scramb1), has a modulatory function in the process of neurotransmission. They have a significant role in scrambling of phospholipids across the plasma membrane during apoptosis. [Date last reviewed: 2019-03-14] |
| DS10_00012279 | N/A | Molecular function is unknown. Biological process is unknown. Top Nucleotide BLAST result: PREDICTED: Drosophila suzukii transmembrane protein 53 (LOC108014739), transcript variant X3, mRNA Top BLAST PRotein sequence: PREDICTED: transmembrane protein 53 isoform X2 [Drosophila suzukii] | 6.17040262618295e-08 | Dmel\CG8245 |
| DS10_00009813 | No results | No results Top Nucleotide BLAST result: PREDICTED: Drosophila suzukii uncharacterized LOC108006065 (LOC108006065), ncRNA Top Protein BLAST result:hypothetical protein [Methylocystis rosea] | 6.21646829547749e-08 | No results |
| DS10_00009743 | CheA87a | Molecular function is unknown. Biological process: sensory perception of chemical stimulus. | 6.67295438624134e-08 | Dmel\CheA87a  Chemosensory protein A 87a |
| DS10_00005037 | RpL7A | Molecular function: structural constituent of ribosome. Biological process: centrosome organization; mitotic spindle elongation; mitotic spindle organization; centrosome duplication. | 6.9872217150541e-08 | Dmel\RpL7A  Ribosomal protein L7A ([RpL7A](http://flybase.org/search/RpL7A)) encodes a ribosomal structural constituent. [Date last reviewed: 2019-07-11] |
| DS10_00006327 | RpS15Aa | Molecular function: structural constituent of ribosome. Biological process: mitotic spindle elongation; mitotic spindle organization. | 6.93231566395894e-08 | TWO ORTHOLOGS: Dmel\RpS15Ab Ribosomal protein S15Aa AND Ribosomal protein S15Ab |
| DS10_00009252 | N/A | Molecular function is unknown. Biological process is unknownTop nucleotide BLAST result: PREDICTED: Drosophila suzukii gamma-interferon-inducible lysosomal thiol reductase (LOC108007466), transcript variant X11, mRNA Top PROTEIN BLAST RESULT: PREDICTED: gamma-interferon-inducible lysosomal thiol reductase isoform X10 [Drosophila suzukii] | 6.96592112815141e-08 | Dmel\CG41378v |
| DS10_00008004 | N/A | Molecular function is unknown. Biological process is unknown. Top nucleotide BLAST result: PREDICTED: Drosophila suzukii uncharacterized LOC108010418 (LOC108010418), mRNA Top PROTEIN BLAST result: PREDICTED: uncharacterized protein LOC108010418 [Drosophila suzukii] | 7.24186692345697e-08 | Dmel\CG6290 |
| DS10_00002910 | Pal1 | Molecular function: peptidylamidoglycolate lyase activity. Biological process: oxidation-reduction process; peptide metabolic process. | 7.42812424838639e-08 | MULTIPLE RESULTS see Tuesday, May 12, 2020 |
| DS10_00009029 | GstD5 | Molecular function: glutathione transferase activity. Biological process: glutathione metabolic process. | 7.53283479294248e-08 | Dmel\GstD5  Glutathione S transferase D5 |
| DS10_00007208 | mp | Molecular function: carbohydrate binding. Biological process: motor neuron axon guidance. | 8.11904186843095e-08 | Dmel\Mp  Multiplexin ([Mp](http://flybase.org/search/Mp)) encodes a Collagen XV/XVIII type protein that functions in motor axon pathfinding. It is required for normal calcium channel abundance, presynaptic calcium influx, and neurotransmitter release. [Date last reviewed: 2019-03-14] |
| DS10_00010557 | N/A | Molecular function: cytokine activity. Biological process: signal transduction. | 8.13547994532172e-08 | Dmel\Gbp3  Growth-blocking peptide 3 |
| DS10_00006986 | N/A | Molecular function: sodium:iodide symporter activity. Biological process: transmembrane transport. | 8.24392296004458e-08 | Dmel\CG9657 |
| DS10_00003485 | lox2 | Molecular function: protein-lysine 6-oxidase activity. Biological process: cell adhesion; cellular protein modification process. | 8.30659805932338e-08 | Dmel\Loxl2  Lysyl oxidase-like 2 |
| DS10_00001529 | Tep2 | Molecular function: peptidase inhibitor activity. Biological process: defense response to Gram-negative bacterium; antibacterial humoral response; phagocytosis, engulfment | 8.70278821486262e-08 | Dmel\Tep2  Thioester-containing protein 2 ([Tep2](http://flybase.org/search/Tep2)) is a member of the Thioester-containing proteins (TEP) family of genes. The product of [Tep2](http://flybase.org/search/Tep2) has endopeptidase inhibitor activity and participates in the cellular immune response to certain Gram-negative bacteria. [Date last reviewed: 2019-03-21] |
| DS10_00005845 | N/A | Molecular function is unknown. Biological process is unknown.Top Nucleotide BLAST result: PREDICTED: Drosophila suzukii prohormone-3 (LOC108017822), mRNA Top Protein BLAST result: PREDICTED: prohormone-3 [Drosophila suzukii] | 8.79742828628868e-08 | Dmel\spab  space blanket |
| DS10_00009995 | Spn88Eb | Molecular function: serine-type endopeptidase inhibitor activity. Biological process: chaeta development. | 8.7758868209027e-08 | Dmel\Spn88Eb  Serpin 88Eb |
| DS10_00007799 | Hn | Molecular function: phenylalanine 4-monooxygenase activity. Biological process: L-phenylalanine catabolic process; eye pigment biosynthetic process; long-term memory; phagocytosis, engulfment. | 9.36632434382285e-08 | Dmel\Hn  Henna ([Hn](http://flybase.org/search/Hn)) encodes a tryptophan phenylalanine hydroxylase. It is a dual function enzyme: it hydroxylates both phenylalanine to generate tyrosine, as well as tryptophan to generate the precursor for peripheral (non-neuronal) serotonin. It is also involved in pteridine synthesis. [Date last reviewed: 2019-03-07] |
| DS10_00005360 | wupA | Molecular function: tropomyosin binding; actin binding. Biological process: heart development; myofibril assembly; skeletal muscle tissue development; nervous system development; muscle organ development; nuclear division; muscle cell homeostasis; sarcomere organization; cardiac muscle tissue development. | 9.41635372375292e-08 | wings up A (CG7178, FBgn0283471) see TUESDAY MAY, 12, 2020 |
| DS10_00000925 | N/A | Molecular function: serine-type endopeptidase activity. Biological process: proteolysis. | 9.72098226616955e-08 | TWO: Dmel\CG31827 AND Dmel\CG18478 |
| DS10_00010577 | N/A | Molecular function: calcium ion binding. Biological process: phagocytosis, engulfment. | 9.90322700250676e-08 | Dmel\CG31345 |
| DS10_00000587 | N/A | Molecular function: zinc ion binding. Biological process is unknown. | 9.95040622181599e-08 | Dmel\CG2991 |
| DS10_00004961 | su(r) | Molecular function: dihydropyrimidine dehydrogenase (NADP+) activity. Biological process: 'de novo' pyrimidine nucleobase biosynthetic process. | 1.00250748826756e-07 | Dmel\su(r)  suppressor of rudimentary ([su(r)](http://flybase.org/search/su%28r%29)) encodes a dihydropyrimidine dehydrogenase, the first step of pyrimidine catabolism. [su(r)](http://flybase.org/search/su%28r%29) depletion does not produce visible phenotypes, but synergistic effects are exhibited by combining mutants with genes affecting pyrimidine biosynthesis and beta-alanine metabolism. [Date last reviewed: 2019-09-26] |
| DS10_00002571 | N/A | Molecular function is unknown. Biological process is unknown. Top Nucleotide BLAST: PREDICTED: Drosophila suzukii uncharacterized LOC108008608 (LOC108008608), transcript variant X1, mRNA Top protein BLAST: PREDICTED: uncharacterized protein LOC108008608 isoform X1 [Drosophila suzukii] | 1.03699306596376e-07 | Dmel\CG10205 |
| DS10_00009199 | RpL13A | Molecular function: structural constituent of ribosome. Biological process: Notch signaling pathway; chaeta morphogenesis; centrosome duplication. | 1.10518085319091e-07 | Dmel\RpL13A  Ribosomal protein L13A ([RpL13A](http://flybase.org/search/RpL13A)) encodes a component of the large ribosomal subunit, and thus involved in mRNA translation. Mutations in genes encoding ribosomal proteins affect fertility, viability and other Drosophila phenotypes, described as Minute. [Date last reviewed: 2019-03-14] |
| DS10_00003119 | N/A | Molecular function is unknown. Biological process is unknown. Top Nucleotide BLAST result:PREDICTED: Drosophila suzukii uncharacterized LOC108008796 (LOC108008796), mRNA top Protein BLAST result: PREDICTED: uncharacterized protein LOC108008796 [Drosophila suzukii] | 1.12200855281211e-07 | Dmel\CG5773 |
| DS10_00000042 | RpLP1 | Molecular function: structural constituent of ribosome. Biological process: translation. | 1.21530707809009e-07 | Dmel\RpLP1  Ribosomal protein LP1 ([RpLP1](http://flybase.org/search/RpLP1)) encodes a member of the cytoplasmic large ribosomal protein family. [Date last reviewed: 2018-09-20] |
| DS10_00000628 | N/A | Molecular function is unknown. Biological process is unknown. Top Nucleotide BLAST: PREDICTED: Drosophila suzukii microfibril-associated glycoprotein 4-like (LOC108009013), mRNA top protein BLAST: PREDICTED: microfibril-associated glycoprotein 4-like [Drosophila suzukii] | 1.22172256636702e-07 | Dmel\CG9500 |
| DS10_00003582 | RpL18A | Molecular function: structural constituent of ribosome. Biological process: mitotic spindle elongation; mitotic spindle organization; centrosome duplication. | 1.25282771124602e-07 | Dmel\RpL18A |
| DS10_00004189 | ImpL3 | Molecular function: L-lactate dehydrogenase activity. Biological process: glycolysis. | 1.34700886570955e-07 | Dmel\Ldh  Lactate dehydrogenase ([Ldh](http://flybase.org/search/Ldh)) encodes a protein involved in myoblast fusion and somatic muscle development. [Date last reviewed: 2019-09-19] |
| DS10_00008502 | RpS8 | Molecular function: structural constituent of ribosome. Biological process: neurogenesis. | 1.43039636792109e-07 | Dmel\RpS8  Ribosomal protein S8 ([RpS8](http://flybase.org/search/RpS8)) encodes a structural constituent of ribosomes that is involved in larval lymph gland hemopoiesis. [Date last reviewed: 2019-07-11] |
| DS10_00003076 | eIF3-S8 | Molecular function: translation initiation factor activity. Biological process: translational initiation | 1.47648710576136e-07 | Dmel\eIF3c  eukaryotic translation initiation factor 3 subunit c |
| DS10_00008873 | prc | Molecular function is unknown. Biological process: heart development. | 1.55980659538039e-07 | Dmel\prc  pericardin ([prc](http://flybase.org/search/prc)) encodes a type IV collagen-like extracellular matrix protein involved in heart development. [Date last reviewed: 2019-03-14 |
| DS10_00001882 | RpL9 | Molecular function: structural constituent of ribosome. Biological process: mitotic spindle elongation; mitotic spindle organization; centrosome duplication. | 1.60939391085344e-07 | RpL9  Ribosomal protein L9 |
| DS10_00007898 | Cht7 | Molecular function: heparin binding; chitinase activity. Biological process: chitin catabolic process; carbohydrate metabolic process. | 1.63318468553879e-07 | Dmel\Cht7  Chitinase 7 ([Cht7](http://flybase.org/search/Cht7)) encodes an enzyme involved in cuticle development, ecdysis and wound healing. [Date last reviewed: 2019-07-11] |
| DS10_00008658 | Ugt86Dd | Molecular function: glucuronosyltransferase activity. Biological process: metabolic process. | 1.78167635994786e-07 | Ugt86Dd  UDP-glycosyltransferase family 35 member C1 |
| DS10_00003610 | RpL19 | Molecular function: structural constituent of ribosome. Biological process: mitotic spindle elongation; centrosome duplication. | 1.81409410315693e-07 | Ribosomal protein L19 (CG2746, FBgn0285950)  Ribosomal protein L19 ([RpL19](http://flybase.org/search/RpL19)) encodes a ribosomal structural constituent  Dmel\RpL19 |
| DS10_00005132 | sta | Molecular function: structural constituent of ribosome; ribosome binding. Biological process: mitotic spindle elongation; mitotic spindle organization. | 1.83328942973701e-07 | Dmel\sta  Stubarista  The gene stubarista is referred to in FlyBase by the symbol Dmel\sta (CG14792, FBgn0003517). It is a protein_coding_gene from Dmel. It has 5 annotated transcripts and 5 polypeptides (1 unique). Gene sequence location is X:1481797..1483413. Its molecular function is described by: ribosome binding; structural constituent of ribosome. It is involved in the biological process described with: translation; ribosomal small subunit assembly; cytoplasmic translation. 35 alleles are reported. The phenotypes of these alleles manifest in: cellular anatomical entity; multi-tissue structure; macrochaeta; I band; embryonic/larval somatic muscle. The phenotypic classes of alleles include: phenotype; wild-type; increased mortality; developmental rate defective. Summary of modENCODE Temporal Expression Profile: Temporal profile ranges from a peak of extremely high expression to a trough of very high expression. Peak expression observed at stages throughout embryogenesis, at stages throughout the larval period, during early pupal stages, in adult female stages. |
| DS10_00009427 | N/A | Molecular function is unknown. Biological process is unknown. Top nucleotide BLAST result: PREDICTED: Drosophila suzukii uncharacterized LOC108016467 (LOC108016467), mRNA Top Protein BLAST result: PREDICTED: uncharacterized protein LOC108016467 [Drosophila suzukii] | 1.8637661851009e-07 | Multiple results, see Tuesday May 5 2020 |
| DS10_00009440 | RpS7 | Molecular function: structural constituent of ribosome. Biological process: translation | 1.89831145204479e-07 | Dmel\RpS7  Ribosomal protein S7 |
| DS10_00011736 | RpS4 | Molecular function: structural constituent of ribosome. Biological process: centrosome organization; mitotic spindle elongation; mitotic spindle organization; centrosome duplication | 1.9179996925411e-07 | Dmel\RpS4  Ribosomal protein S4 |
| DS10_00003297 | N/A | Molecular function: nucleotide binding; fatty-acyl-CoA reductase (alcohol-forming) activity. Biological process is unknown. | 1.92778006995592e-07 | Dmel\CG8303  Predicted to have fatty-acyl-CoA reductase (alcohol-forming) activity. Predicted to be involved in long-chain fatty-acyl-CoA metabolic process and wax biosynthetic process. Predicted to localize to integral component of peroxisomal membrane. Is expressed in adult head; embryonic/larval posterior spiracle; foregut; and presumptive embryonic/larval system. |
| DS10_00005662 | N/A | Molecular function: calcium ion binding; phospholipase A2 activity. Biological process: lipid catabolic process; phospholipid metabolic process. | 1.9424083962714e-07 | Dmel\CG30503  Predicted to have calcium-dependent phospholipase A2 activity. Predicted to be involved in arachidonic acid secretion and phospholipid metabolic process. Orthologous to human PLA2G3 (phospholipase A2 group III). |
| DS10_00007508 | N/A | Molecular function: calcium, potassium:sodium antiporter activity. Biological process: transmembrane transport. | 1.94396917228521e-07 | Multiple results, see Tuesday May 5, 2020 |
| DS10_00006904 | Gtp-bp | Molecular function: signal recognition particle binding. Biological process: regulation of protein secretion; axonogenesis. | 1.99419993212684e-07v | Dmel\SrpRα  Signal recognition particle receptor α  Predicted to have GTPase activity and signal recognition particle binding activity. Involved in axonogenesis and regulation of protein secretion. Localizes to endoplasmic reticulum membrane. Is expressed in several structures, including early extended germ band embryo; embryonic/larval digestive system; embryonic/larval peripheral nervous system; presumptive embryonic/larval system; and proventriculus primordium. Orthologous to human SRPRA (SRP receptor subunit alpha). |
| DS10_00002865 | Pex6 | Molecular function: nucleoside-triphosphatase activity; ATP binding. Biological process: peroxisome organization. | 2.06714927605556e-07 | Dmel\Pex6  Adenosinetriphosphatase  Predicted to have ATP binding activity. Involved in peroxisome organization. Predicted to localize to peroxisome. Human ortholog(s) of this gene implicated in peroxisomal biogenesis disorder and peroxisome biogenesis disorder 4A. Orthologous to human PEX6 (peroxisomal biogenesis factor 6). |
| DS10_00004540 | LysS | Molecular function: lysozyme activity. Biological process: antimicrobial humoral response. | 2.07886455649023e-07 | Dmel\LysS  Predicted to have lysozyme activity. Predicted to be involved in defense response to Gram-negative bacterium. Predicted to localize to extracellular space. Human ortholog(s) of this gene implicated in familial visceral amyloidosis. Is expressed in gastric caecum. |
| DS10_00012109 | alphaTub84D | Molecular function: protein binding. Biological process: cellular process; cytokinesis. | 2.08326777634013e-07 | Dmel\αTub84D  α-Tubulin at 84D ([αTub84D](http://flybase.org/search/%CE%B1Tub84D)) encodes a protein involved in major cellular mechanisms such as formation of spindle apparatus or microtubular threads for transport of proteins or organelles. [Date last reviewed: 2019-03-21] |
| DS10_00002147 | RpL23 | Molecular function: protein binding. Biological process: mitotic spindle elongation; mitotic spindle organization; neurogenesis. | 2.10162510186901e-07 | Dmel\RpL23Exhibits myosin binding activity. Predicted to be involved in cytoplasmic translation. Predicted to localize to cytosolic large ribosomal subunit. Is expressed in adult head and adult heart. |
| DS10_00005992 | N/A | Molecular function is unknown. Biological process is unknown. Top nucleotide BLAST result: PREDICTED: Drosophila suzukii leucine-rich repeat-containing protein 58 (LOC108019101), mRNA Top protein BLAST result: PREDICTED: leucine-rich repeat-containing protein 58 [Drosophila suzukii] | 2.26683807916599e-07 | Dmel\CG32687  Is expressed in embryonic/larval fat body; embryonic/larval midgut; embryonic/larval posterior spiracle; yolk; and yolk nucleus. Orthologous to human LRRC58 (leucine rich repeat containing 58). |
| DS10_00003232 | N/A | Molecular function is unknown. Biological process is unknown. Top nucleotide BLASt function; PREDICTED: Drosophila suzukii uncharacterized LOC108009690 (LOC108009690), mRNA top protein BLAST function: PREDICTED: uncharacterized protein LOC108009690 [Drosophila suzukii] | 2.42821973704851e-07 | Dmel\CG11275 Predicted to have ubiquitin protein ligase binding activity. Predicted to be involved in proteasome-mediated ubiquitin-dependent protein catabolic process and regulation of proteolysis. Predicted to localize to cytoplasm and nucleus. Is expressed in several structures, including atrium; extended germ band embryo; labral sensory complex primordium; presumptive embryonic/larval system; and spermatozoon. |
| DS10_00013269 | RpL17 | Molecular function: structural constituent of ribosome. Biological process: mitotic spindle elongation; mitotic spindle organization; centrosome duplication. | 2.42406013478618e-07 | Dmel\RpL17  Predicted to be a structural constituent of ribosome. Predicted to be involved in cytoplasmic translation. Predicted to localize to cytosolic large ribosomal subunit. Is expressed in adult head; adult heart; and organism |
| DS10_00011610  POTENTIAL DOUBLE GENE SEE PUG, SEE DS10_00011607 | pug | Molecular function: methylenetetrahydrofolate dehydrogenase (NADP+) activity; formate-tetrahydrofolate ligase activity; methenyltetrahydrofolate cyclohydrolase activity. Biological process: oxidation-reduction process; folic acid-containing compound biosynthetic process. | 2.47101136526648e-07 | Dmel\pug  pugilist ([pug](http://flybase.org/search/pug)) encodes the trifunctional enzyme methylenetetrahydrofolate dehydrogenase involved in the pigmentation of pteridines and ommochromes. |
| DS10_00000915 | N/A | Molecular function: metalloendopeptidase activity. Biological process: neurogenesis. | 2.58920522317344e-07 | Dmel\CG15255  Predicted to have metalloendopeptidase activity. Predicted to be involved in proteolysis |
| DS10_00001343 | crc | Molecular function: RNA polymerase II activating transcription factor binding; RNA polymerase II transcription coactivator activity; RNA polymerase II distal enhancer sequence-specific DNA binding. Biological process: metamorphosis; molting cycle, chitin-based cuticle; pupation; positive regulation of transcription from RNA polymerase II promoter; pupariation | 2.70146080404919e-07 | Dmel\crc  cryptocephal ([crc](http://flybase.org/search/crc)) encodes a protein that belongs to a member of the CREB/ATF family of basic-leucine zipper transcription factors. It serves as an coactivator of the product [EcR](http://flybase.org/search/EcR) of to promote expression the molting peptide hormone encoded by [ETH](http://flybase.org/search/ETH). It also acts as an unfolded protein response transcription factor to regulate glycolytic genes in response to ER stress. |
| DS10_00009502 | N/A | Molecular function is unknown. Biological process: wing disc dorsal/ventral pattern formation. | 2.77134447959482e-07 | Dmel\CG8369  Involved in wing disc dorsal/ventral pattern formation. |
| DS10_00013336 | GstD9 | Molecular function: glutathione transferase activity. Biological process: glutathione metabolic process. | 2.82832823150901e-07 | Dmel\GstD9  Exhibits glutathione transferase activity. Involved in glutathione metabolic process. Predicted to localize to cytoplasm. |
| DS10_00003849 | RpS17 | Molecular function: structural constituent of ribosome. Biological process: translation. | 2.87556027549343e-07 | RpS17  Ribosomal protein S17 ([RpS17](http://flybase.org/search/RpS17)) encodes an essential component of the ribosomal 40S subunit. [RpS17](http://flybase.org/search/RpS17) mutants have dominant, haploinsufficient effects on cellular and organismal growth rate, and on bristle size. [Date last reviewed: 2019-03-14] |
| DS10_00011728 | RpL5 | Molecular function: protein binding; structural constituent of ribosome. Biological process: translation. | 2.89372860109445e-07 | Dmel\RpL5  Ribosomal protein L5 ([RpL5](http://flybase.org/search/RpL5)) encodes a component of the large subunit of cytoplasmic ribosomes, which translate mRNAs encoded by the nuclear genome. [RpL5](http://flybase.org/search/RpL5) is haploinsufficient - heterozygous mutants display the 'Minute' phenotype, characterized by a slower developmental rate and small adult bristles. [Date last reviewed: 2019-03-14] |
| DS10_00004135 | N/A | Molecular function: inositol oxygenase activity; iron ion binding. Biological process: oxidation-reduction process; inositol catabolic process. | 2.91972876826383e-07 | Dmel\CG6910  Predicted to have inositol oxygenase activity. Predicted to be involved in inositol catabolic process. Predicted to localize to cytoplasm. Is expressed in cardiogenic mesoderm; embryonic/larval fat body; fat body/gonad primordium; and trunk mesoderm. |
| DS10_00011043 | RpL21 | Molecular function: structural constituent of ribosome. Biological process: mitotic spindle elongation; mitotic spindle organization; centrosome duplication. | 2.91220147366408e-07 | Dmel\RpL21  A structural constituent of ribosome. Predicted to be involved in cytoplasmic translation. Localizes to cytosolic ribosome. Human ortholog(s) of this gene implicated in hypotrichosis 12. Is expressed in adult head; adult heart; and organism |
| DS10_00013539 | RpS14a | Molecular function: structural constituent of ribosome. Biological process: translation. | 2.95600303767231e-07 | RpS14a  A structural constituent of ribosome. Predicted to be involved in maturation of SSU-rRNA from tricistronic rRNA transcript (SSU-rRNA, 5.8S rRNA, LSU-rRNA); ribosomal small subunit assembly; and translation. Localizes to cytosolic ribosome. Is expressed in adult heart and organism. |
| DS10_00005509 | Mtk | Molecular function is unknown. Biological process: defense response to fungus; defense response to Gram-positive bacterium; antifungal humoral response; defense response to Gram-negative bacterium; defense response; antibacterial humoral response. | 2.98561022747511e-07 | Dmel\Mtk Metchnikowin ([Mtk](http://flybase.org/search/Mtk)) encodes an antifungal peptide that is secreted from the fat body during the systemic immune response, and is produced by various epithelia. Its expression is regulated at the transcriptional level by the immune deficiency and/or Toll pathways. |
| DS10_00002112 | RpS16 | Molecular function: structural constituent of ribosome. Biological process: mitotic spindle elongation; mitotic spindle organization. | 3.16123974263718e-07 | Dmel\RpS16  A structural constituent of ribosome. Predicted to be involved in maturation of SSU-rRNA from tricistronic rRNA transcript (SSU-rRNA, 5.8S rRNA, LSU-rRNA). Localizes to cytosolic ribosome. Is expressed in adult head; adult heart; and organism. |
| DS10_00012636 | N/A | Molecular function: calcium ion binding. Biological process: neuron projection morphogenesis. | 3.29774464161505e-07 | Dmel\CG31475 Predicted to have calcium ion binding activity. Predicted to be involved in calcium-ion regulated exocytosis. Predicted to localize to endoplasmic reticulum. Is expressed in embryonic proventriculus and embryonic proventriculus intermediate layer. |
| DS10_00005418 | N/A | Molecular function: phospholipase A2 activity. Biological process: lipid catabolic process; phospholipid metabolic process. | 3.3482187429065e-07 | Dmel\CG3009 Predicted to have calcium-dependent phospholipase A2 activity. Predicted to be involved in arachidonic acid secretion and phospholipid metabolic process. Is expressed in adult head. |
| DS10_00001238 | RpL30 | Molecular function: structural constituent of ribosome. Biological process: growth; mitotic spindle elongation; mitotic spindle organization; centrosome duplication. | 3.66776519728975e-07 | Dmel\RpL30  A structural constituent of ribosome. Predicted to be involved in cytoplasmic translation. Localizes to cytosolic ribosome. Human ortholog(s) of this gene implicated in herpes simplex. Is expressed in adult head; adult heart; medulla; optic lobe forming neuroblast; and organism. |
| DS10_00010990 | N/A | Molecular function: rRNA (adenine-N6,N6-)-dimethyltransferase activity. Biological process: neurogenesis. | 3.69900886250359e-07 | Dmel\CG11837 Predicted to have rRNA (adenine-N6,N6-)-dimethyltransferase activity. Predicted to be involved in rRNA methylation. Predicted to localize to mitochondrial matrix. Is expressed in several structures, including embryonic/larval midgut; embryonic/larval muscle system; and extended germ band embryo. |
| DS10_00011137 | l(3)72Dp | Molecular function: omega peptidase activity. Biological process: glutamine metabolic process. | 4.07989119237657e-07 | Dmel\l(3)72Dp lethal (3) 72Dp Folate gamma-glutamyl hydrolase Predicted to have gamma-glutamyl-peptidase activity. Predicted to be involved in tetrahydrofolylpolyglutamate metabolic process. Predicted to localize to vacuole. Is expressed in adult head |
| DS10_00007560 | N/A | Molecular function: phosphotransferase activity, alcohol group as acceptor. Biological process: carbohydrate metabolic process. | 4.30555391743739e-07 | Dmel\CG11594 D-ribulokinase Predicted to have D-ribulokinase activity. Involved in response to endoplasmic reticulum stress. Orthologous to human FGGY (FGGY carbohydrate kinase domain containing). |
| DS10_00011867 | N/A | Molecular function: protein binding. Biological process is unknown | 4.46218934500469e-07 | Dmel\CG6834 Exhibits myosin binding activity. Is expressed in adult head and spermatozoon. |
| DS10_00002297 | RpL12 | Molecular function: structural constituent of ribosome. Biological process: mitotic spindle elongation. | 4.54218969867673e-07 | RpL12 Ribosomal protein L12 ([RpL12](http://flybase.org/search/RpL12)) encodes a protein of the large subunit of the ribosome. It regulates transcription of ribosomal proteins and ribosome biogenesis genes as well as interacting with the Enhancer of Trithorax and Polycomb protein encoded by [corto](http://flybase.org/search/corto). |
| DS10_00010875 | N/A | Molecular function: adenosine kinase activity. Biological process: purine ribonucleoside salvage. | 4.54238830254035e-07 | Dmel\Adk2 Predicted to have adenosine kinase activity. Predicted to be involved in AMP biosynthetic process. Localizes to cytosol and nucleoplasm. Human ortholog(s) of this gene implicated in hypermethioninemia due to adenosine kinase deficiency. Is expressed in several structures, including adult head; embryonic/larval midgut; embryonic/larval midgut primordium; and embryonic/larval muscle system |
| DS10_00000528 | N/A | Molecular function is unknown. Biological process is unknown. Top nucleotide BLASt result:PREDICTED: Drosophila suzukii uncharacterized LOC108010421 (LOC108010421), mRNA top Protein BLAST result: PREDICTED: uncharacterized protein LOC108010421 [Drosophila suzukii] | 4.64111510129087e-07 | TWO ORTHOLOGS: Dmel\CG42694 Predicted to be involved in proteolysis AND Dmel\CG30187 Predicted to have serine-type endopeptidase activity. Predicted to be involved in proteolysis |
| DS10_00002569 | N/A | Molecular function is unknown. Biological process is unknown. | 4.69296212254466e-07 | Dmel\hui hase und igel (German for rabbit and hedgehog) Involved in wing disc development. Is expressed in anterior-posterior compartment boundary of the wing disc; embryonic foregut; embryonic proventriculus; and embryonic rectum. |
| DS10_00003379 | N/A | Molecular function: receptor binding. Biological process: negative regulation of transforming growth factor beta receptor signaling pathway. | 4.81082976714614e-07 | Dmel\CG10459 |
| DS10_00007492 | N/A | Molecular function is unknown. Biological process is unknown. Top NCBI nucletotide result: REDICTED: Drosophila suzukii venom allergen 3 (LOC108006535), mRNA Top PROTEIN BLAST: PREDICTED: venom allergen 3 [Drosophila suzukii] | 4.83749617114185e-07 | Dmel\CG9400 Predicted to be involved in multicellular organism reproduction. Predicted to localize to extracellular space. Is expressed in embryonic hypopharynx. |
| DS10_00001279 | N/A | Molecular function is unknown. Biological process is unknown Top nucleotide BLAST result: PREDICTED: Drosophila suzukii keratin, type I cytoskeletal 9 (LOC108005130), transcript variant X2, mRNA top PRotein BLASt result: PREDICTED: keratin, type I cytoskeletal 9 isoform X1 [Drosophila suzukii] | 4.89111208443507e-07 | Dmel\CG17549 Is expressed in several structures, including early extended germ band embryo; foregut; labral sensory complex; presumptive embryonic/larval system; and sensory nervous system primordium. |
| DS10_00007389 | RpL6 | Molecular function: structural constituent of ribosome. Biological process: centrosome organization; mitotic spindle elongation; mitotic spindle organization; centrosome duplication. | 5.02766558064806e-07 | RpL6 A structural constituent of ribosome. Predicted to be involved in cytoplasmic translation and ribosomal large subunit assembly. Localizes to cytosolic ribosome. Is expressed in adult head; adult heart; and organism. |
| DS10_00003581 | N/A | Molecular function is unknown. Biological process: cilium assembly | 5.07003384050724e-07 | Dmel\Cc2d2a  Coiled-coil and C2 domain containing 2A Predicted to be involved in cilium assembly. Localizes to ciliary transition zone. |
| DS10_00010843 | RpL10 | Molecular function: structural constituent of ribosome. Biological process: centrosome organization; mitotic spindle elongation; mitotic spindle organization; centrosome duplication. | 5.11892913108555e-07 | Dmel\RpL10 A structural constituent of ribosome. Predicted to be involved in ribosomal large subunit assembly. Localizes to cytoplasm and neuronal cell body. |
| DS10_00009056 | N/A | Molecular function: UDP-galactose transmembrane transporter activity. Biological process: transmembrane transport. | 5.65440973280237e-07 | Dmel\meigo medial glomeruli ([meigo](http://flybase.org/search/meigo)) encodes an evolutionarily conserved, multi-membrane pass protein that is mainly localized at the endoplasmic reticulum. [meigo](http://flybase.org/search/meigo) genetically interacts with [Ephrin](http://flybase.org/search/Ephrin) in dendrite targeting of olfactory projection neurons |
| DS10_00013318 | No results (really) | No results Top nucleotide BLAST PREDICTED: Drosophila suzukii uncharacterized LOC108015675 (LOC108015675), ncRNA top protein BLAST: No significant similarity found [it’s likely too short-TWS] | 5.72229995659084e-07 | No results |
| DS10_00011374 | RpS27 | Molecular function: structural constituent of ribosome. Biological process: translation. | 5.76078617850532e-07 | Dmel\RpS27 A structural constituent of ribosome. Predicted to be involved in ribosomal small subunit assembly. Localizes to cytosolic ribosome. |
| DS10_00005566 | St4 | Molecular function: sulfotransferase activity. Biological process: sulfation; xenobiotic metabolic process. | 5.78754926488784e-07 | Dmel\St4 Sulfotransferase 4 ([St4](http://flybase.org/search/St4)) encodes one out of 4 sulfotransferases that catalyze the sulfonation of many proteins, lipids and glycosaminoglycans. |
| DS10_00007873 | No results | No results Top nucleotide BLAST result: PREDICTED: Drosophila suzukii trypsin delta/gamma (LOC108007084), partial mRNA top protein blast: PREDICTED: uncharacterized protein LOC108040370 [Drosophila rhopaloa] | 5.82295905733582e-07 | No results |
| DS10_00010782 | N/A | Molecular function is unknown. Biological process is unknown. | 5.88341437039775e-07 | Dmel\CG42390 Predicted to contribute to GTPase activator activity. Predicted to localize to cytoplasm and nuclear pore. Is expressed in germline cell and spermatozoon. |
| DS10_00009565 | Gfat1 | Molecular function: glutamine-fructose-6-phosphate transaminase (isomerizing) activity. Biological process: carbohydrate biosynthetic process | 5.96393285317952e-07 | Multiple results, SEE wednesday may 13 2020 |
| DS10_00004931 | RpS28b | Molecular function: structural constituent of ribosome. Biological process: translation. | 6.00075884715535e-07 | Dmel\RpS28b Predicted to be a structural constituent of ribosome. Predicted to be involved in maturation of SSU-rRNA and ribosomal small subunit assembly. Predicted to localize to cytosolic small ribosomal subunit. |
| DS10_00007221 | Dbi | Molecular function: diazepam binding; enzyme inhibitor activity; fatty-acyl-CoA binding. Biological process: cellular acyl-CoA homeostasis. | 6.0895845773873e-07 | Dmel\Acbp2 Acyl-CoA binding protein 2 Predicted to have fatty-acyl-CoA binding activity. Predicted to be involved in cellular acyl-CoA homeostasis. Is expressed in several structures, including adult heart; amnioserosa; embryonic/larval fat body; gut section; and yolk nucleus. |
| DS10_00002351 | N/A | Molecular function: cysteine-type endopeptidase activity. Biological process: proteolysis. | 6.25488326935338e-07 | Dmel\CG13423 Predicted to have cysteine-type peptidase activity. Predicted to be involved in homocysteine catabolic process and response to toxic substance. Predicted to localize to cytoplasm |
| DS10_00002488 | Tango7 | Molecular function: catalytic activity. Biological process: neuron projection morphogenesis; Golgi organization; protein secretion. | 6.37210934120385e-07 | Dmel\eIF3m eukaryotic translation initiation factor 3 subunit m ([eIF3m](http://flybase.org/search/eIF3m)) encodes a non core component of the EIF3 translation initiation factor. The product of [eIF3m](http://flybase.org/search/eIF3m) functions during caspase mediated remodeling of the syncytial testis, where it interacts with the apoptosome. |
| DS10_00005661 | sPLA2 | Molecular function: calcium-dependent phospholipase A2 activity. Biological process: lipid catabolic process; phospholipid metabolic process. | 6.41337421214105e-07 | Dmel\sPLA2 Exhibits calcium-dependent phospholipase A2 activity. Predicted to be involved in arachidonic acid secretion and phospholipid metabolic process. Is expressed in organism. |
| DS10_00011478 | N/A | Molecular function is unknown. Biological process: transmembrane transport; intracellular signal transduction | 6.91993580238465e-07 | Dmel\anchor Predicted to be involved in intracellular signal transduction and transmembrane transport. Predicted to localize to integral component of membrane. |
| DS10_00001984 | N/A | Molecular function: glycosylphosphatidylinositol diacylglycerol-lyase activity; phosphoric diester hydrolase activity. Biological process: glycerophospholipid metabolic process. | 7.10531120250361e-07 | Dmel\PLCXD Phosphatidylinositol-specific phospholipase C X domain containing Predicted to have glycosylphosphatidylinositol diacylglycerol-lyase activity and phosphoric diester hydrolase activity. Predicted to be involved in glycerophospholipid metabolic process. |
| DS10_00008298 | Dscam3 | Molecular function: identical protein binding. Biological process: cell adhesion. | 7.08648308261022e-07 | Dscam3 Down syndrome cell adhesion molecule 3 ([Dscam3](http://flybase.org/search/Dscam3)) encodes a large transmembrane protein that functions in homophilic cell adhesion. It may also function as an axon guidance receptor. |
| DS10_00011382 | N/A | Molecular function: transferase activity, transferring phosphorus-containing groups. Biological process is unknown. | 7.08940029199719e-07 | Dmel\CG6908 nvolved in copper ion homeostasis. |
| DS10_00012838 | N/A | Molecular function: ATP binding. Biological process is unknown. | 7.24381836879089e-07 | Dmel\CG4511 Is expressed in several structures, including dorsal head epidermis; embryonic/larval digestive system; embryonic/larval muscle system; and germ layer derivative |
| DS10_00005180 | Actn | Molecular function: calcium ion binding; actin filament binding; actin binding. Biological process: flight behavior; sarcomere organization; actin cytoskeleton reorganization. | 7.39218352831171e-07 | Dmel\Actn α actinin ([Actn](http://flybase.org/search/Actn)) encodes an actin cross-linking protein with muscle and non-muscle specific isoforms, which are produced by alternative spicing. The muscle isoform has a role in muscle development while the non-muscle isoform localizes to ovarian ring canals and has a role in cytoskeletal remodeling of follicle cells. |
| DS10_00008511 | N/A | Molecular function: structural constituent of cuticle. Biological process is unknown. | 7.40189843416319e-07 | Dmel\CG15515 Predicted to be a structural constituent of cuticle. |
| DS10_00012557 | N/A | Molecular function is unknown. Biological process is unknown. | 7.62522181585933e-07 | Dmel\CG13999 Predicted to be involved in cilium assembly. Predicted to localize to cilium. |
| DS10_00008214 | RpS19a | Molecular function: structural constituent of ribosome. Biological process: translation. | 7.78407726775492e-07 | Dmel\RpS19a Ribosomal protein S19a ([RpS19a](http://flybase.org/search/RpS19a)) encodes a structural constituent of ribosomes |
| DS10_00007316 | N/A | Molecular function: cation channel activity. Biological process: monovalent inorganic cation transport. | 8.47624099150282e-07 | Dmel\CG4239 Predicted to have cation channel activity. Predicted to be involved in monovalent inorganic cation transport. Predicted to localize to membrane. |
| DS10_00002748 | N/A | Molecular function: snoRNA binding; rRNA pseudouridylation guide activity. Biological process: neurogenesis. | 9.06605239301835e-07 | Dmel\CG4038 Required for ribosome biogenesis. Part of a complex which catalyzes pseudouridylation of rRNA. This involves the isomerization of uridine such that the ribose is subsequently attached to C5, instead of the normal N1. Pseudouridine ('psi') residues may serve to stabilize the conformation of rRNAs (By similarity). |
| DS10_00003754 | RpL14 | Molecular function: structural constituent of ribosome. Biological process: centrosome organization; mitotic spindle elongation; negative regulation of neuron apoptotic process; mitotic spindle organization; centrosome duplication | 9.07452917040275e-07 | Dmel\RpL14 Ribosomal protein L14 ([RpL14](http://flybase.org/search/RpL14)) encodes a component of the large subunit of cytoplasmic ribosomes, which translate mRNAs encoded by the nuclear genome. [RpL14](http://flybase.org/search/RpL14) is haploinsufficient - heterozygous mutants display the 'Minute' phenotype, characterized by a slower developmental rate and small adult bristles |
| DS10_00008505 | RpS8 | Molecular function: structural constituent of ribosome. Biological process: neurogenesis. | 9.28085635864589e-07 | Dmel\RpS8 Ribosomal protein S8 ([RpS8](http://flybase.org/search/RpS8)) encodes a structural constituent of ribosomes that is involved in larval lymph gland hemopoiesis. |
| DS10_00011980 | N/A | Molecular function: protein binding. Biological process is unknown. Top nucleotide BLAst:PREDICTED: Drosophila suzukii uncharacterized LOC108014730 (LOC108014730), mRNA Top protein BLAST: PREDICTED: uncharacterized protein LOC108014730 [Drosophila suzukii] | 9.44213772261942e-07 | Dmel\CG6834 Exhibits myosin binding activity. Is expressed in adult head and spermatozoon. |
| DS10_00000125 | N/A | Molecular function: carbohydrate binding. Biological process is unknown. | 9.58174957847843e-07 | Dmel\CG15818 Predicted to have carbohydrate binding activity. Is expressed in embryonic proventriculus and embryonic/larval midgut. C-type lectins are Ca^2+^-dependent carbohydrate-binding proteins. They possess a C-type lectin fold, that is also present in proteins that do not bind carbohydrates |
| DS10_00002119 | RpS24 | Molecular function: structural constituent of ribosome. Biological process: translation. | 9.79274467295466e-07 | Dmel\RpS24 Ribosomal protein S24 ([RpS24](http://flybase.org/search/RpS24)) encodes an essential component of the small ribosomal subunit that functions in protein synthesis. It is classified as a 'Minute' gene as heterozygous mutants exhibit a slower developmental rate and small adult bristles. |
| DS10_00011609  POTENTIAL TRIPLE GENE, see  DS10_00011610  DS10_00011607 | pug | Molecular function: methylenetetrahydrofolate dehydrogenase (NADP+) activity; formate-tetrahydrofolate ligase activity; methenyltetrahydrofolate cyclohydrolase activity. Biological process: oxidation-reduction process; folic acid-containing compound biosynthetic process. | 1.02104685186648e-06 | Dmel\pug pugilist ([pug](http://flybase.org/search/pug)) encodes the trifunctional enzyme methylenetetrahydrofolate dehydrogenase involved in the pigmentation of pteridines and ommochromes |
| DS10_00013445 | No results | No results top nucleotide result: PREDICTED: Drosophila suzukii uncharacterized LOC108005040 (LOC108005040), mRNA top protein blast result: PREDICTED: uncharacterized protein LOC108005040 [Drosophila suzukii] | 1.10334561811796e-06 | No results |
| DS10_00007173 | kin17 | Molecular function: nucleic acid binding. Biological process: mRNA splicing, via spliceosome | 1.11427841655158e-06 | Dmel\kin17 Predicted to have double-stranded DNA binding activity. Predicted to be involved in DNA replication and cellular response to DNA damage stimulus. Localizes to precatalytic spliceosome. |
| DS10_00008201 | N/A | Molecular function: glycine hydroxymethyltransferase activity. Biological process: behavioral response to ethanol. | 1.1316354474121e-06 | Dmel\Shmt Serine hydroxymethyl transferase ([Shmt](http://flybase.org/search/Shmt)) encodes a conserved pyridoxal phosphate-containing enzyme that converts serine into glycine and N5,N10-methylentetrahydrofolate. This reaction represents a major source for activated C1 units and tetrahydrofolate-mediated C1 metabolism |
| DS10_00004696 | N/A | Molecular function: oxidoreductase activity, acting on CH-OH group of donors. Biological process: metabolic process. | 1.15294353599179e-06 | Dmel\CG3699 Predicted to have 2,4-dienoyl-CoA reductase (NADPH) activity; estradiol 17-beta-dehydrogenase activity; and testosterone 17-beta-dehydrogenase (NADP+) activity. |
| DS10_00009619 | N/A | Molecular function is unknown. Biological process is unknown.Top nucleotide blast: PREDICTED: Drosophila suzukii GILT-like protein F37H8.5 (LOC108017466), transcript variant X2, mRNA top protein blast: PREDICTED: GILT-like protein F37H8.5 isoform X2 [Drosophila suzukii] | 1.15582761812641e-06 | Dmel\CG9427 |
| DS10_00012015 | N/A | Molecular function is unknown. Biological process is unknown top nucleotide BLAST: PREDICTED: Drosophila suzukii serine-rich adhesin for platelets (LOC108018265), mRNA top protein BLAST:PREDICTED: LOW QUALITY PROTEIN: serine-rich adhesin for platelets [Drosophila suzukii | 1.15058095979024e-06 | Dmel\CG15544 Is expressed in Bolwig organ; dorsal head epidermis; embryonic antennal sense organ; embryonic head epidermis; and embryonic head sensory system |
| DS10_00000443 | RpL7 | Molecular function: structural constituent of ribosome. Biological process: mitotic spindle elongation; mitotic spindle organization; centrosome duplication; pupariation. | 1.17767956896077e-06 | Dmel\RpL7A structural constituent of ribosome. Involved in pupariation. Localizes to cytosolic ribosome. |
| DS10_00005621 | Tsp42Ed | Molecular function is unknown. Biological process is unknown top nucleotide result: PREDICTED: Drosophila suzukii CD63 antigen (LOC108017755), mRNA top protein BLAst result:PREDICTED: CD63 antigen [Drosophila suzukii] | 1.17893631870133e-06 | Dmel\Tsp42Ed Predicted to localize to integral component of plasma membrane. Is expressed in adult CNS glial cell; adult head |
| DS10_00007574 | Gad1 | Molecular function: glutamate decarboxylase activity. Biological process: response to mechanical stimulus; olfactory learning; synapse assembly; gamma-aminobutyric acid biosynthetic process; neuromuscular junction development; neurotransmitter receptor metabolic process; glutamate catabolic process; larval locomotory behavior. | 1.17859689029e-06 | Gad1 Glutamic acid decarboxylase 1 ([Gad1](http://flybase.org/search/Gad1)) encodes an essential, nervous system-specific glutamic acid decarboxylase, which is the synthetic enzyme for the major inhibitory neurotransmitter gamma-Aminobutyric acid (GABA). It is required for a multitude of physiological functions and adult behaviors dependent on GABA, including sleep, memory, circadian rhythms and egg hatching |
| DS10_00005368 | BoYb | Molecular function: ATP binding; ATP-dependent helicase activity; nucleic acid binding. Biological process: negative regulation of transposition, RNA-mediated. | 1.19875817341291e-06 | Dmel\BoYb Brother of Yb ([BoYb](http://flybase.org/search/BoYb)) encodes a putative RNA helicase in the TDRD12 family and is predicted to have a role in piRNA biogenesis, specifically in ovarian germline cells |
| DS10_00003528 | N/A | Molecular function is unknown. Biological process is unknown.Top nucleotide BLAST PREDICTED: Drosophila suzukii uncharacterized protein KIAA0930 homolog (LOC108009321), transcript variant X1, mRNA top protein BLAST: PREDICTED: uncharacterized protein LOC108009321 isoform X1 [Drosophila suzukii] | 1.20622448140624e-06 | Dmel\CG9646 |
| DS10_00012537 | N/A | Molecular function is unknown. Biological process is unknown top Nucleotide BLAST: PREDICTED: Drosophila suzukii uncharacterized LOC108015974 (LOC108015974), transcript variant X2, mRNA top protein BLAST: PREDICTED: uncharacterized protein LOC108015974 isoform X1 [Drosophila suzukii] | 1.22579542245928e-06 | Dmel\CG7695 Is expressed in adult fat body; antennal disc; eye disc; ventral thoracic disc; and wing disc. |
| DS10_00011571 | N/A | Molecular function is unknown. Biological process is unknown. Top nucleotide BLAST: PREDICTED: Drosophila suzukii uncharacterized F-box/LRR-repeat protein C02F5.7-like (LOC108010737), mRNA top protein BLAST: PREDICTED: uncharacterized F-box/LRR-repeat protein C02F5.7-like [Drosophila suzukii] | 1.24924999517429e-06 | Dmel\FipoQ Predicted to contribute to ubiquitin-protein transferase activity. Involved in positive regulation of protein ubiquitination. Predicted to localize to SCF ubiquitin ligase complex. F box and Leucine-rich repeat (FBXL) proteins are F box proteins which contain leucine-rich repeats. F box proteins are the variable substrate adapters for Skp, Cullin, F-box (SCF) E3 ubiquitin ligase complexes. |
| DS10_00002664 | Cpr47Ec | Molecular function: structural constituent of chitin-based cuticle. Biological process is unknown | 1.27404591611723e-06 | Dmel\Cpr47Ec Predicted to be a structural constituent of chitin-based larval cuticle. Predicted to be involved in chitin-based cuticle development. Predicted to localize to chitin-based extracellular matrix. |
| DS10_00000423 | RpL13 | Molecular function: structural constituent of ribosome. Biological process: mitotic spindle elongation; mitotic spindle organization; centrosome duplication. | 1.2902099869988e-06 | Dmel\RpL13 A structural constituent of ribosome. Predicted to be involved in cytoplasmic translation. Localizes to cytosolic ribosome. Is expressed in adult head and adult heart. |
| DS10_00005528 | N/A | Molecular function: chitin binding. Biological process: chitin metabolic process. | 1.28952717421211e-06 | Dmel\CG8192 Predicted to have chitin binding activity. Predicted to be involved in chitin metabolic process. Predicted to localize to extracellular region. |
| DS10_00005371 | N/A | Molecular function: Mo-molybdopterin cofactor sulfurase activity. Biological process: Mo-molybdopterin cofactor biosynthetic process. | 1.30308889583144e-06 | Dmel\Uba5 Ubiquitin-like activating enzyme 5 ([Uba5](http://flybase.org/search/Uba5)) encodes a member of the E1-like ubiquitin-activating enzyme family. It activates the protein encoded by [Ufm1](http://flybase.org/search/Ufm1), and acts as a key factor in the ufmylation process. It is involved in ATP binding, catalytic activity, cofactor binding, metal ion binding, nucleotide binding and oxidoreductase activity |
| DS10_00007021 | N/A | Molecular function: acetyl-CoA C-acetyltransferase activity. Biological process: mitotic spindle organization | 1.32541185196577e-06 | Dmel\CG10932 Predicted to have acetyl-CoA C-acetyltransferase activity. Predicted to be involved in fatty acid beta-oxidation; fatty acid biosynthetic process; and pyruvate metabolic process. Localizes to mitochondrion. |
| DS10_00003288 | N/A | Molecular function: chitin binding. Biological process: cuticle chitin catabolic process. | 1.36900366895291e-06 | Dmel\Idgf6 Imaginal disc growth factor 6 ([Idgf6](http://flybase.org/search/Idgf6)) is one of six genes in the IDGF family, which evolved from chitinases that lost their enzymatic activity. [Idgf6](http://flybase.org/search/Idgf6) encodes a secreted protein that is highly expressed in larval fat body and adult head. [Idgf6](http://flybase.org/search/Idgf6) product is involved in molting and egg chamber tube morphogenesis. |
| DS10_00006652 | nemy | Molecular function: carbon-monoxide oxygenase activity. Biological process: locomotory behavior; memory; imaginal disc-derived wing morphogenesis. | 1.47883374443273e-06 | Dmel\nemy no extended memory ([nemy](http://flybase.org/search/nemy)) likely encodes a human cytochrome b561 homolog and is involved in middle-term olfactory associative memory and courtship conditioning memory. |
| DS10_00003541 | N/A | Molecular function: cytokine activity. Biological process: signal transduction. | 1.52419672080553e-06 | Dmel\Gbp2 Growth-blocking peptide 2 Predicted to have N-acetyltransferase activity and cytokine activity. Predicted to be involved in signal transduction. Localizes to extracellular region. |
| DS10_00000227 | N/A | Molecular function is unknown. Biological process is unknown. Top nucleotide BLAST result: PREDICTED: Drosophila suzukii calphotin (LOC108008964), mRNA top protein BLAST: PREDICTED: calphotin [Drosophila suzukii] | 1.53227413442095e-06 | Dmel\CG15414 |
| DS10_00001038 | N/A | Molecular function: serine-type endopeptidase activity. Biological process: proteolysis | 1.53312669208625e-06 | Dmel\CG4259 Predicted to be involved in proteolysis. |
| DS10_00006787 | N/A | Molecular function: serine-type endopeptidase activity. Biological process: proteolysis. | 1.57352836133689e-06 | Dmel\CG8738 Predicted to have serine-type endopeptidase activity. Predicted to be involved in proteolysis. Predicted to localize to extracellular space. Serine protease homologs of the S1A family are similar in amino acid sequence to S1A serine proteases but are enzymatically inactive due to the lack of catalytic residue(s). Their functions are generally poorly understood, though at least some of them have known regulatory functions |
| DS10_00003864 | N/a | Molecular function is unknown. Biological process is unknown. Top nucleotide BLAST: PREDICTED: Drosophila suzukii uncharacterized LOC108012974 (LOC108012974), transcript variant X2, mRNA To Protein BLAST: PREDICTED: uncharacterized protein LOC108012974 [Drosophila suzukii] | 1.58715212398295e-06 | Dmel\CG32037 Is expressed in dorsal epidermis primordium; embryonic dorsal apodeme; embryonic dorsal epidermis; and embryonic head sensory system. |
| DS10_00009353 | N/A | Molecular function is unknown. Biological process is unknown Top nucleotide BLAST: PREDICTED: Drosophila suzukii E3 UFM1-protein ligase 1 homolog (LOC108017127), mRNA Top Protein BLAST: PREDICTED: E3 UFM1-protein ligase 1 homolog [Drosophila suzukii] | 1.59799920594029e-06 | Dmel\Ufl1 Predicted to have UFM1 ligase activity. Predicted to be involved in protein K69-linked ufmylation; regulation of proteasomal ubiquitin-dependent protein catabolic process; and response to endoplasmic reticulum stress. |
| DS10_00005487 | kcc | Molecular function: potassium:chloride symporter activity. Biological process: response to mechanical stimulus; mechanosensory behavior. | 1.71830639311035e-06 | Dmel\kcc kazachoc ([kcc](http://flybase.org/search/kcc)) encodes a potassium:chloride symporter that contributes to seizure susceptibility |
| DS10_00004153 | No results | No results top nucleotide BLAST: PREDICTED: Drosophila suzukii brachyurin-like (LOC108012409), mRNA top protien BLAST: hypothetical protein FF38_07427 [Lucilia cuprina]; lots of serine-9 results, including this 5th result: PREDICTED: transmembrane protease serine 9-like [Drosophila suzukii] | 1.75360121385628e-06 | No results |
| DS10_00002388 | Cyp6a22 | Molecular function: electron carrier activity. Biological process: oxidation-reduction process | 1.79238952187713e-06 | Dmel\Cyp6a22 Predicted to have heme binding activity; iron ion binding activity; and oxidoreductase activity, acting on paired donors, with incorporation or reduction of molecular oxygen. Predicted to be involved in oxidation-reduction process |
| DS10_00007732 | knk | Molecular function is unknown. Biological process: single-organism developmental process; biological regulation; open tracheal system development; system development; organonitrogen compound metabolic process; amino sugar biosynthetic process; regulation of tube architecture, open tracheal system; regulation of anatomical structure size; amino sugar metabolic process; chitin-based cuticle development; anterior/posterior axis specification. | 1.81167736111653e-06 | Dmel\knk knickkopf ([knk](http://flybase.org/search/knk)) encodes a GPI-anchorded protein, needed for chitin organisation in the cuticle and the tracheal system (tracheal cuticle, tube diameter regulation & gas filling) |
| DS10_00012899 | RpL32 | Molecular function: structural constituent of ribosome. Biological process: mitotic spindle elongation; mitotic spindle organization. | 1.81254718880061e-06 | Dmel\RpL32 Predicted to be a structural constituent of ribosome. Predicted to be involved in cytoplasmic translation. Localizes to cytoplasm and nucleolus. Colocalizes with nuclear chromosome. |
| DS10_00004628 | Kaz1-ORFB | Molecular function: serine-type endopeptidase inhibitor activity. Biological process: regulation of proteolysis. | 1.83501435746973e-06 | Dmel\Kaz1-ORFB Localizes to extracellular region and mitochondrion. Is expressed in several structures, including embryonic/larval midgut; embryonic/larval midgut primordium; and embryonic/larval muscle system |
| DS10_00007624 | eIF3-S10 | Molecular function: translation initiation factor activity. Biological process: mitotic spindle elongation; mitotic spindle organization. | 1.83987207929551e-06 | Dmel\eIF3a eukaryotic translation initiation factor 3 subunit a Predicted to contribute to translation initiation factor activity. Predicted to be involved in formation of cytoplasmic translation initiation complex; regulation of translational initiation; and translation reinitiation. Localizes to cytosol. |
| DS10_00002043 | N/A | Molecular function is unknown. Biological process is unknown Top nucleotide Blast: PREDICTED: Drosophila suzukii uncharacterized LOC108008462 (LOC108008462), mRNA to protien BLAST: PREDICTED: uncharacterized protein LOC108008462 [Drosophila suzukii] | 1.90048772858763e-06 | Dmel\CG16926 |
| DS10_00006475 | N/A | Molecular function is unknown. Biological process: cell adhesion. Top nucleotide BLAST: PREDICTED: Drosophila suzukii fibrinogen-like protein A (LOC108005499), mRNAto Protein BLAST: PREDICTED: fibrinogen-like protein A [Drosophila suzukii] | 1.908941321736e-06 | Dmel\CG6788 |
| DS10_00007379 | N/A | Molecular function: translation regulator activity. Biological process: wing disc development. | 1.92566689272211e-06 | Dmel\CG11334 Predicted to have S-methyl-5-thioribose-1-phosphate isomerase activity. Predicted to be involved in L-methionine salvage from methylthioadenosine |
| DS10_00004637 | Tudor-SN | Molecular function: transcription coactivator activity. Biological process: gene silencing by RNA | 1.9944080222498e-06 | Dmel\Tudor-SN Tudor staphylococcal nuclease Exhibits nuclease activity. Involved in negative regulation of transposition and spermatogenesis. Localizes to RISC complex; cytoplasm; and nucleus. |
| DS10_00005567 | Aats-phe | Molecular function: phenylalanine-tRNA ligase activity. Biological process: phenylalanyl-tRNA aminoacylation. | 1.99236331898443e-06 | Phenylalanyl-tRNA synthetase, mitochondrial (CG13348, FBgn0275436) |
| DS10_00013563 | RpS25 | Molecular function: structural constituent of ribosome. Biological process: translation. | 1.98435776218718e-06 | Dmel\RpS25 Ribosomal protein S25 A structural constituent of ribosome. Predicted to be involved in cytoplasmic translation. Localizes to cytosolic ribosome |
| DS10_00001856 | Nos | Molecular function: nitric-oxide synthase activity. Biological process: nervous system development. | 2.00290478084e-06 | Dmel\Nos Exhibits nitric-oxide synthase activity. Involved in nervous system development and regulation of heart rate. Predicted to localize to several cellular components, including cytosol; peroxisome; and vesicle membrane. |
| DS10_00011817 | wtrw | Molecular function: calcium channel activity. Biological process: sensory perception of sound; response to humidity. | 2.00542124566301e-06 | Dmel\wtrw water witch ([wtrw](http://flybase.org/search/wtrw)) encodes a protein involved in response to humidity and sound perception |
| DS10_00001118 | N/A | Molecular function: serine-type endopeptidase activity. Biological process: proteolysis | 2.01969190939462e-06 | Dmel\CG8738 Predicted to have serine-type endopeptidase activity. Predicted to be involved in proteolysis. Predicted to localize to extracellular space |
| DS10_00000329 | N/A | Molecular function: phospholipase activity. Biological process: lipid metabolic process. | 2.03315727398355e-06 | Dmel\CG4267 Predicted to have lipase activity. Predicted to be involved in lipid catabolic process. Predicted to localize to extracellular space |
| DS10_00001037 | GlyP | Molecular function: glycogen phosphorylase activity; pyridoxal phosphate binding; protein homodimerization activity. Biological process: glycogen catabolic process; flight. | 2.04260324874361e-06 | Dmel\GlyP Exhibits glycogen phosphorylase activity; protein homodimerization activity; and pyridoxal phosphate binding activity. Involved in several processes, including glycogen catabolic process; insulin receptor signaling pathway; and positive regulation of glycogen catabolic process. |
| DS10_00005493 | Hex-C | Molecular function: hexokinase activity. Biological process: lateral inhibition | 2.05973373121396e-06 | Dmel\Hex-C Hexokinase C ([Hex-C](http://flybase.org/search/Hex-C)) encodes a hexokinase involved in glucose homeostasis |
| DS10_00006245 | RpL37a | Molecular function: structural constituent of ribosome. Biological process: translation | 2.08090362511227e-06 | Dmel\RpL37a Insufficient genetic data for FlyBase to solicit a summary |
| DS10_00006786 | N/A | Molecular function: serine-type endopeptidase activity. Biological process: proteolysis | 2.11562101769747e-06 | Dmel\CG8586 Predicted to have serine-type endopeptidase activity. Predicted to be involved in proteolysis. Localizes to extracellular region |
| DS10_00007229 | RpL18 | Molecular function: structural constituent of ribosome. Biological process: mitotic spindle elongation; mitotic spindle organization. | 2.1794534946545e-06 | Dmel\RpL18  A structural constituent of ribosome. Predicted to be involved in cytoplasmic translation. Localizes to cytosolic ribosome. Human ortholog(s) of this gene implicated in Diamond-Blackfan anemia. Is expressed in adult head; adult heart; and organism. Orthologous to human RPL18 (ribosomal protein L18). |
| DS10_00011483 | N/A | Molecular function: pyrroline-5-carboxylate reductase activity. Biological process: oxidation-reduction process; proline biosynthetic process | 2.19350820721725e-06 | Dmel\P5cr-2 Predicted to have pyrroline-5-carboxylate reductase activity. Predicted to be involved in L-proline biosynthetic process. Human ortholog(s) of this gene implicated in autosomal recessive cutis laxa type IIB; autosomal recessive cutis laxa type IIIB; and hypomyelinating leukodystrophy 10. |
| DS10_00000793 | Got2 | Molecular function: L-aspartate:2-oxoglutarate aminotransferase activity. Biological process: synapse assembly; glutamate biosynthetic process; neurotransmitter receptor metabolic process. | 2.26668696745837e-06 | Dmel\Got2 Glutamate oxaloacetate transaminase 2 ([Got2](http://flybase.org/search/Got2)) encodes an L- aspartate:2-oxoglutarate aminotransferase involved in glutamate biosynthesis, which in turn regulates postsynaptic receptor field size. [Date last reviewed: 2019-09-12] |
| DS10_00010806 | N/A | Molecular function: catalytic activity. Biological process: metabolic process | 2.27789964704167e-06 | Dmel\CG5793 Predicted to have acetylpyruvate hydrolase activity. Predicted to localize to mitochondrion. Is expressed in adult head and adult heart |
| DS10_00009975 | Su(var)3-9 | Molecular function: histone methyltransferase activity (H3-K9 specific); histone methyltransferase activity. Biological process: cellular component organization or biogenesis; biological regulation; organelle organization; macromolecule modification; negative regulation of cellular biosynthetic process; gene silencing; chromosome organization; multicellular organism reproduction; gamete generation; macromolecule methylation. | 2.28710221713637e-06 | **TWO GENES**: Dmel\Su(var)3-9 Suppressor of variegation 3-9, Exhibits chromatin binding activity and histone methyltransferase activity (H3-K9 specific) **AND** Dmel\eIF2γ eukaryotic translation initiation factor 2 subunit gamma Predicted to contribute to tRNA binding activity. Predicted to be involved in formation of translation preinitiation complex and positive regulation of translational fidelity. eIF-2 functions in the early steps of protein synthesis by forming a ternary complex with GTP and initiator tRNA. This complex binds to a 40S ribosomal subunit, followed by mRNA binding to form a 43S pre-initiation complex. |
| DS10_00000292 | lectin-21Cb | olecular function: carbohydrate binding; mannose binding. Biological process is unknown. | 2.40799937814425e-06 | Dmel\lectin-21Cb Predicted to have carbohydrate binding activity. |
| DS10_00002353 | N/A | Molecular function is unknown. Biological process is unknown. Top Nuclotide BLAST: PREDICTED: Drosophila suzukii gram-negative bacteria-binding protein 3 (LOC108008798), mRN Top protein BLAST: PREDICTED: gram-negative bacteria-binding protein 3 [Drosophila suzukii] | 2.40372913717146e-06 | Dmel\CG30148 Predicted to have carbohydrate binding activity. |
| DS10_00013346 | N/A | Molecular function: N-acetylgalactosamine-4-sulfatase activity. Biological process: defense response to bacterium. | 2.55291098037562e-06 | Dmel\CG7408 Predicted to have sulfuric ester hydrolase activity. Involved in defense response to bacterium |
| DS10_00000798 | N/A | Molecular function: ubiquitin-protein ligase activity. Biological process is unknown | 2.55931722130236e-06 | Dmel\CG4238 Predicted to have ubiquitin protein ligase activity. Predicted to be involved in cellular protein metabolic process; negative regulation of apoptotic process; and positive regulation of protein catabolic process. |
| DS10_00009477 | N/A | Molecular function: O-phospho-L-serine:2-oxoglutarate aminotransferase activity. Biological process: pyridoxine biosynthetic process; L-serine biosynthetic process. | 2.63409030107104e-06 | Dmel\CG11899 Phosphoserine transaminase Predicted to have O-phospho-L-serine:2-oxoglutarate aminotransferase activity. Predicted to be involved in L-serine biosynthetic process. Predicted to localize to cytoplasm. |
| DS10_00003449 | Rpi | Molecular function: ribose-5-phosphate isomerase activity. Biological process: pentose-phosphate shunt, non-oxidative branch. | 2.86376632701494e-06 | Dmel\Rpi Predicted to have ribose-5-phosphate isomerase activity. Involved in age-dependent response to reactive oxygen species. Predicted to localize to intracellular membrane-bounded organelle. |
| DS10_00011820 | Pdi | Molecular function: protein disulfide isomerase activity. Biological process: protein folding. | 2.87325466996277e-06 | MULTIPLE RESULTS SEE Sunday May 24, 2020 [FBgn0014002](http://flybase.org/reports/FBgn0014002.html) |
| DS10_00008006 | No results | No results Top nucleotide result: PREDICTED: Drosophila takahashii uncharacterized LOC108061005 (LOC108061005), mRNA top protein BLAST result: PREDICTED: uncharacterized protein LOC101890539 [Musca domestica] | 2.95146795991164e-06 | No results |
| DS10_00012948 | Bap55 | Molecular function: transcription coactivator activity. Biological process: positive regulation of gene silencing by miRNA; dendrite morphogenesis; mitosis; cytokinesis; muscle organ development; positive regulation of transcription, DNA-dependent; dendrite guidance | 2.99926009454867e-06 | Dmel\Bap55 Brahma associated protein 55kD (Bap55) encodes a member of two chromatin remodeling complexes. As part of the Brahma complex, it is needed for cell growth and survival in the wing imaginal disc; as a member of the TIP60 complex, it is thought to regulate dendrite wiring specificity in olfactory projection neurons. |
| DS10_00008932 | N/A | Molecular function: 4-nitrophenylphosphatase activity. Biological process: metabolic process. | 3.06621847526967e-06 | Dmel\CG5567 Alkaline phosphatase ([3.1.3.1](https://enzyme.expasy.org/EC/3.1.3.1))  Protein-serine/threonine phosphatase Predicted to have alkaline phosphatase activity and phosphoprotein phosphatase activity. Predicted to be involved in protein dephosphorylation |
| DS10_00008816 | RpL26 | Molecular function: structural constituent of ribosome. Biological process: mitotic spindle elongation; mitotic spindle organization. | 3.08118885172099e-06 | Dmel\RpL26 Ribosomal protein L26 ([RpL26](http://flybase.org/search/RpL26)) encodes a protein component of large ribosomal subunits. |
| DS10_00008791 | glob1 | Molecular function: oxygen transporter activity. Biological process: oxygen transport. | 3.13161009283604e-06 | Dmel\glob1 globin 1 ([glob1](http://flybase.org/search/glob1)) encodes a peroxidase involved in the response to oxidative stress and the maintenance of F-actin based cytoskeleton |
| DS10_00005715 | Rgk2 | Molecular function: GTPase activity; GTP binding. Biological process: GTP catabolic process; small GTPase mediated signal transduction. | 3.1583398842607e-06 | Dmel\Rgk2 Rad, Gem/Kir family member 2 ([Rgk2](http://flybase.org/search/Rgk2)) encodes a protein that regulates voltage-gated calcium channel activity. |
| DS10_00002220 | RpL29 | Molecular function: chromatin binding. Biological process: translation | 3.19298544152132e-06 | Dmel\RpL29 Exhibits chromatin binding activity. Predicted to be involved in cytoplasmic translation. Predicted to localize to cytosolic large ribosomal subunit. |
| DS10_00005291 | N/A | Molecular function: ribokinase activity. Biological process: D-ribose metabolic process. | 3.21762342853303e-06 | Dmel\CG13369 Predicted to have ribokinase activity. Predicted to be involved in D-ribose metabolic process. Predicted to localize to cytoplasm and nucleus. |
| DS10_00006856 | up | Molecular function: calcium ion binding. Biological process: cellular calcium ion homeostasis; myofibril assembly; muscle organ morphogenesis; muscle cell homeostasis; sarcomere organization; mitochondrion organization; skeletal muscle thin filament assembly; mesoderm development. | 3.22120105025331e-06 | Dmel\up upheld ([up](http://flybase.org/search/up)) encodes the striated muscle protein Troponin T. Troponins T, C and I form a regulatory complex with Tropomyosin that is found at regular intervals along the thin (F-actin) filaments of the muscle sarcomere |
| DS10_00011740 | N/A | Molecular function is unknown. Biological process is unknown. Top Nucleotide BLAST result: PREDICTED: Drosophila suzukii extensin (LOC108011268), mRNA Top protein BLAST result: PREDICTED: extensin [Drosophila suzukii] | 3.21646353622798e-06 | Dmel\CG14598 s expressed in embryonic dorsal epidermis and embryonic/larval posterior spiracle |
| DS10_00012710 | N/A | Molecular function is unknown. Biological process: neurogenesis. | 3.23708450775607e-06 | Dmel\CG7394 Insufficient genetic data for FlyBase to solicit a summary |
| DS10_00005730 | No results | No results Top nucleotide blast:PREDICTED: Drosophila suzukii glutathione S-transferase 1 (LOC108018222), mRNA top protein BLAST:  PREDICTED: uncharacterized protein LOC108086295 [Drosophila ficusphila] | 3.26695479659757e-06 | No results |
| DS10_00007119 | Syt7 | Molecular function: calcium-dependent phospholipid binding. Biological process: synaptic vesicle exocytosis | 3.45722614374398e-06 | Dmel\Syt7 Synaptotagmin 7 Predicted to have several functions, including SNARE binding activity; calcium ion binding activity; and phospholipid binding activity. |
| DS10_00005837 | RpS15 | Molecular function: structural constituent of ribosome. Biological process: mitotic spindle elongation; mitotic spindle organization. | 3.49805158557861e-06 | Dmel\RpS15 Ribosomal protein S15 ([RpS15](http://flybase.org/search/RpS15)) encodes a protein that plays a role in assembly, structure and function of the ribosome and functions in protein synthesis. It is phosphorylated by the kinase encoded by [Lrrk](http://flybase.org/search/Lrrk), causing protein synthesis upregulation |
| DS10_00002987 | N/A | Molecular function: 5-formyltetrahydrofolate cyclo-ligase activity. Biological process is unknown. | 3.53983351681275e-06 | Dmel\CG9875 Temporal profile ranges from a peak of high expression to a trough of no expression detected. Peak expression observed in adult male stages. |
| DS10_00007248 | sec63 | Molecular function: signal recognition particle binding. Biological process: posttranslational protein targeting to membrane, translocation. | 3.58545080228468e-06 | Dmel\Sec63 Secretory 63 Predicted to have RNA binding activity and protein transmembrane transporter activity. Predicted to be involved in SRP-dependent cotranslational protein targeting to membrane and posttranslational protein targeting to endoplasmic reticulum membrane. Localizes to fusome |
| DS10_00011793 | N/A | Molecular function is unknown. Biological process is unknown Top nucleotide BLAST PREDICTED: Drosophila suzukii uncharacterized LOC108015873 (LOC108015873), mRNA to protein BLAST: PREDICTED: uncharacterized protein LOC108015873 [Drosophila suzukii] | 3.65266632087107e-06 | Dmel\CG14567 |
| DS10_00005801 | Jheh1 | Molecular function: juvenile hormone epoxide hydrolase activity. Biological process: juvenile hormone catabolic process. | 3.70616931895955e-06 | Dmel\Jheh1 Juvenile hormone epoxide hydrolase 1 ([Jheh1](http://flybase.org/search/Jheh1)) expression changes are associated with increased tolerance to oxidative stress. |
| DS10_00004840 | Cbs | Molecular function: cystathionine beta-synthase activity. Biological process: determination of adult lifespan. | 3.73264217015727e-06 | Dmel\Cbs Cystathionine β-synthase ([Cbs](http://flybase.org/search/Cbs)) encodes an enzyme involved in endoplasmic reticulum stress response. |
| DS10_00006192 | Sep4 | Molecular function: GTPase activity. Biological process: cytokinesis | 3.7880842391046e-06 | Dmel\Sep4 Predicted to have GTPase activity and molecular adaptor activity. Predicted to be involved in cellular protein localization and cytoskeleton-dependent cytokinesis. Predicted to localize to cell division site and cytoskeleton. |
| DS10_00009134 | Obp56h | Molecular function: odorant binding. Biological process: sensory perception of smell. | 3.82383318511854e-06 | Dmel\Obp56h Predicted to have odorant binding activity. Involved in mating behavior; mating pheromone secretion; and sensory perception of smell. Predicted to localize to extracellular region. Is expressed in Bolwig organ; embryonic antennal sense organ; and labral sensory complex. |
| DS10_00008178 | N/A | Molecular function: structural molecule activity. Biological process is unknown. | 3.83451302073199e-06 | Dmel\CG15772 Predicted to have structural molecule activity. Is expressed in adult head and organism. |
| DS10_00012475 | RpS3 | Molecular function: oxidized purine nucleobase lesion DNA N-glycosylase activity; structural constituent of ribosome; DNA-(apurinic or apyrimidinic site) lyase activity. Biological process: negative regulation of neuron apoptotic process; response to DNA damage stimulus; DNA repair; mitosis. | 3.85042150404123e-06 | Dmel\RpS3 Ribosomal protein S3 ([RpS3](http://flybase.org/search/RpS3)) encodes a component of the small subunit of cytoplasmic ribosomes, which translate mRNAs encoded by the nuclear genome. [RpS3](http://flybase.org/search/RpS3) is haploinsufficient - heterozygous mutants display the 'Minute' phenotype, characterized by a slower developmental rate and small adult bristles. The product of [RpS3](http://flybase.org/search/RpS3) also contains a DNA lyase activity and functions in DNA repair. |
| DS10_00013162 | RpS3 | Molecular function: oxidized purine nucleobase lesion DNA N-glycosylase activity; structural constituent of ribosome; DNA-(apurinic or apyrimidinic site) lyase activity. Biological process: negative regulation of neuron apoptotic process; response to DNA damage stimulus; DNA repair; mitosis. | 4.01048140794448e-06 | Dmel\RpS3 Ribosomal protein S3 ([RpS3](http://flybase.org/search/RpS3)) encodes a component of the small subunit of cytoplasmic ribosomes, which translate mRNAs encoded by the nuclear genome. [RpS3](http://flybase.org/search/RpS3) is haploinsufficient - heterozygous mutants display the 'Minute' phenotype, characterized by a slower developmental rate and small adult bristles. The product of [RpS3](http://flybase.org/search/RpS3) also contains a DNA lyase activity and functions in DNA repair |
| DS10_00003370 | RpL31 | Molecular function: structural constituent of ribosome. Biological process: mitotic spindle elongation; mitotic spindle organization. | 4.05748419192454e-06 | Dmel\RpL31A structural constituent of ribosome. Predicted to be involved in cytoplasmic translation. Localizes to cytosolic ribosome. Is expressed in adult head and adult heart. |
| DS10_00003829 | UGP | Molecular function: UTP:glucose-1-phosphate uridylyltransferase activity. Biological process: metabolic process. | 4.04508471494567e-06 | Dmel\UGP UGP ([UGP](http://flybase.org/search/UGP)) encodes an enzyme involved in hyperoxia response. UDP-glucose pyrophosphorylase |
| DS10_00008288 | N/A | Molecular function is unknown. Biological process is unknown. Top nucleotide BLAST: PREDICTED: Drosophila suzukii uncharacterized LOC108020831 (LOC108020831), mRNA top protein BLAST: GM15230 [Drosophila sechellia] | 4.05071287895135e-06 | Dmel\CG42821 It is a protein_coding_gene from Dmel. It has 2 annotated transcripts and 2 polypeptides (1 unique). Gene sequence location is 3R:17413077..17413639. Its molecular function is unknown. |
| DS10_00011697 | N/A | Molecular function: transferase activity, transferring acyl groups. Biological process is unknown | 4.06260407766347e-06 | Dmel\CG33337 Predicted to have transferase activity, transferring acyl groups other than amino-acyl groups. Other acyl groups other than amino-acyl group transferases is a collection of transferases that do not fit into any of the other major acyl groups other than amino-acyl group transferases. inferred from electronic annotation with [InterPro:IPR002656](http://www.ebi.ac.uk/interpro/entry/IPR002656) |
| DS10_00006684 | N/A | Molecular function: metallocarboxypeptidase activity. Biological process: proteolysis. | 4.12122297707961e-06 | Dmel\CG2915 Predicted to have metallocarboxypeptidase activity. Predicted to be involved in proteolysis. Localizes to vesicle [metallocarboxypeptidase activity](http://flybase.org/reports/GO:0004181) [zinc ion binding](http://flybase.org/reports/GO:0008270) |
| DS10_00005944 | Trxr-1 | Molecular function: glutathione-disulfide reductase activity; thioredoxin-disulfide reductase activity; antioxidant activity; protein homodimerization activity. Biological process: response to hypoxia; determination of adult lifespan; response to DNA damage stimulus; neurogenesis. | 4.21312985773591e-06 | Trxr-1 Thioredoxin reductase-1 Exhibits glutathione-disulfide reductase activity; protein homodimerization activity; and thioredoxin-disulfide reductase activity. Involved in cell redox homeostasis; determination of adult lifespan; and response to hypoxia. Localizes to mitochondrion. 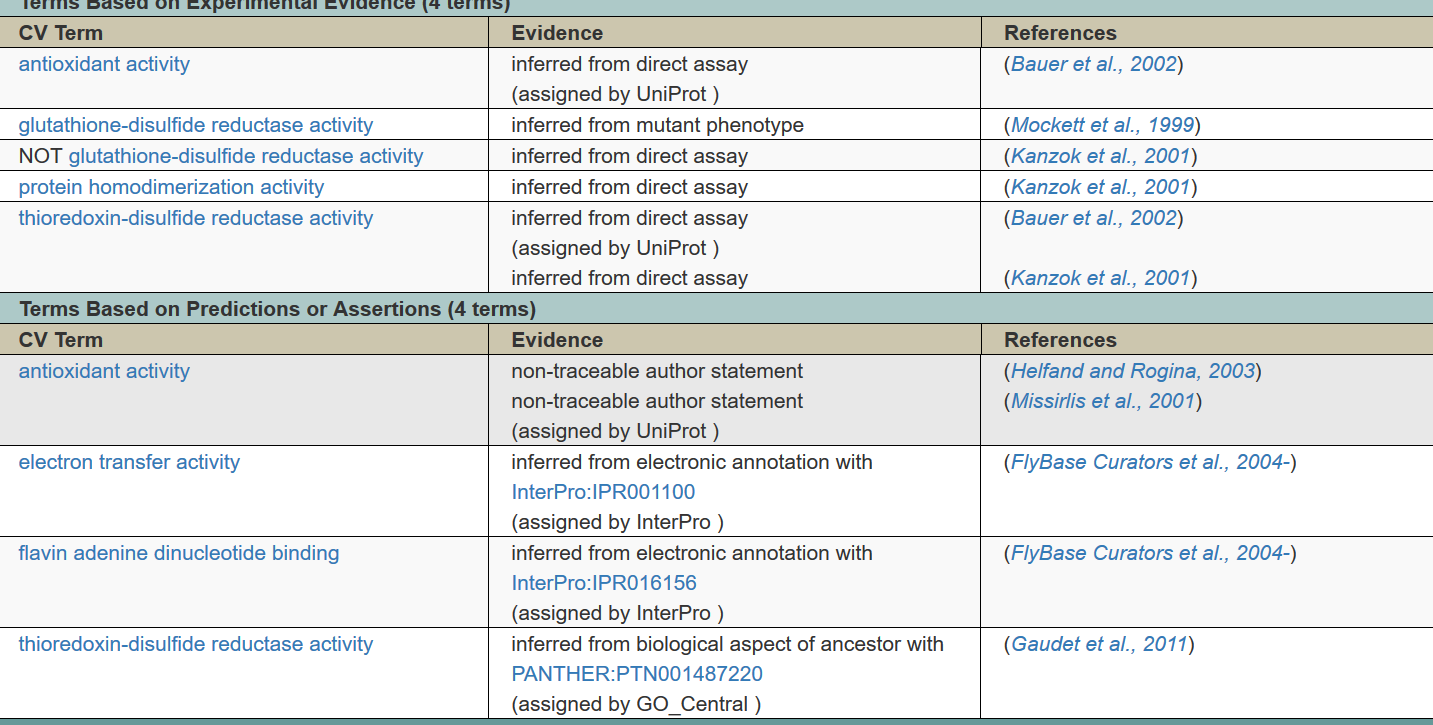 |
| DS10_00005738 | N/A | Molecular function is unknown. Biological process: neurogenesis | 4.42191040299082e-06 | Dmel\BomBc2 Bomanin Bicipital 2 Is expressed in embryonic midgut chamber. The phenotypes of these alleles manifest in: larval neuroblast; ganglion mother cell; mesothoracic tergum; trichogen cell. The phenotypic classes of alleles include: viable; lethal; partially lethal - majority die; visible; neuroanatomy defective.Bomanins are a family of small, secreted immune-induced peptides that are induced by Toll signalling. These proteins may confer resistance to bacterial infection |
| DS10_00011954 | No results | No results Top nucleotide result: PREDICTED: Drosophila suzukii venom protease-like (LOC108011900), mRNA Top protein result: PREDICTED: venom protease-like [Drosophila suzukii] | 4.56644520164589e-06 | No results |
| DS10_00011228 | N/A | Molecular function: phosphoribosylaminoimidazolecarboxamide formyltransferase activity; IMP cyclohydrolase activity. Biological process: wound healing. | 4.62858308858017e-06 | Dmel\CG11089 Phosphoribosylaminoimidazolecarboxamide formyltransferase Predicted to have IMP cyclohydrolase activity and phosphoribosylaminoimidazolecarboxamide formyltransferase activity. Involved in wound healing. Predicted to localize to cytosol |
| DS10_00009638 | N/A | Molecular function: stearoyl-CoA 9-desaturase activity. Biological process: oxidation-reduction process; lipid metabolic process. | 4.72378872986455e-06 | Dmel\CG8630 Stearoyl-CoA 9-desaturase Predicted to have stearoyl-CoA 9-desaturase activity. Predicted to be involved in lipid metabolic process and oxidation-reduction process |
| DS10_00000103 | tutl | Molecular function is unknown. Biological process: synaptic target recognition; regulation of dendrite morphogenesis; dendrite self-avoidance; larval behavior; axon guidance; axonal defasciculation; lateral inhibition; adult locomotory behavior; flight behavior; axon midline choice point recognition | 4.99728951425287e-06 | Dmel\tutl turtle ([tutl](http://flybase.org/search/tutl)) encodes an Ig-superfamily transmembrane protein. Its roles include axonal tiling, dendrite self-avoidance, axonal pathfinding and coordinated motor control |
| DS10_00008399 | Rtc1 | Molecular function: RNA-3'-phosphate cyclase activity. Biological process: RNA processing; ribosome biogenesis | 4.98825567216854e-06 | Dmel\Rtc1 Predicted to have endoribonuclease activity. Predicted to be involved in endonucleolytic cleavage of tricistronic rRNA transcript (SSU-rRNA, 5.8S rRNA, LSU-rRNA). Predicted to localize to nucleolus. Is expressed in embryonic/larval midgut and organism From UniProt: Does not have cyclase activity. Plays a role in 40S-ribosomal-subunit biogenesis in the early pre-rRNA processing steps at sites A0, A1 and A2 that are required for proper maturation of the 18S RNA (By similarity). |
| DS10_00007721 | RpL36A | Molecular function: structural constituent of ribosome. Biological process: mitotic spindle elongation; mitotic spindle organization. | 5.11089171706962e-06 | RpL36A Ribosomal protein L36A Predicted to be a structural constituent of ribosome. Predicted to be involved in cytoplasmic translation. Predicted to localize to cytosolic large ribosomal subunit. Is expressed in adult head. |
| DS10_00001175 | RpS26 | Molecular function: structural constituent of ribosome. Biological process: translation | 5.14698797137078e-06 | Dmel\RpS26 Predicted to have mRNA binding activity. Predicted to be involved in cytoplasmic translation. Localizes to cytosolic small ribosomal subunit. |
| DS10_00000937 | No results | No results Top protein BLAST: PREDICTED: uncharacterized protein LOC108011293 [Drosophila suzukii] top nucleotide BLAST: PREDICTED: Drosophila suzukii uncharacterized LOC108011293 (LOC108011293), mRNA | 5.30414592685154e-06 | No results |
| DS10_00002071 | Swim | Molecular function: Wnt-protein binding. Biological process: positive regulation of Wnt receptor signaling pathway; positive regulation of Wnt receptor signaling pathway by establishment of Wnt protein localization to extracellular region. | 5.30385153146409e-06 | Dmel\Swim Secreted Wg-interacting molecule ([Swim](http://flybase.org/search/Swim)) encodes a secreted protein that binds to the product of [wg](http://flybase.org/search/wg) and may function in its spreading. Exhibits Wnt-protein binding activity. Involved in positive regulation of Wnt signaling pathway by establishment of Wnt protein localization to extracellular region and positive regulation of canonical Wnt signaling pathway. Localizes to chorion and extracellular space. |
| DS10_00006988 | N/A | Molecular function is unknown. Biological process is unknown. Top nucleotide BLAST: PREDICTED: Drosophila suzukii uncharacterized LOC108004626 (LOC108004626), mRNA top protein blast: PREDICTED: uncharacterized protein LOC108004626 [Drosophila suzukii] | 5.28111026159253e-06 | Dmel\CG32726 This gene is referred to in FlyBase by the symbol Dmel\CG32726 (FBgn0052726). It is a protein_coding_gene from Dmel. It has 3 annotated transcripts and 3 polypeptides (1 unique). |
| DS10_00002763 | N/A | Molecular function: lysozyme activity. Biological process: autophagic cell death; salivary gland cell autophagic cell death. | 5.33072301862695e-06 | Dmel\CG11159 Predicted to have lysozyme activity. Predicted to be involved in defense response to Gram-negative bacterium. Predicted to localize to extracellular space. |
| DS10_00003209 | N/A | Molecular function is unknown. Biological process is unknown. Top nucleotide result: PREDICTED: Drosophila suzukii uncharacterized LOC108010178 (LOC108010178), mRNA top protein result: PREDICTED: uncharacterized protein LOC108010178 [Drosophila suzukii] | 5.33542013520474e-06 | Dmel\CG4409 Is expressed in adult head |
| DS10_00005480 | N/A | Molecular function: ATPase activity, coupled to transmembrane movement of substances; transporter activity. Biological process: transport | 5.46339765610904e-06 | Dmel\CG8908 Predicted to have ATPase-coupled transmembrane transporter activity and lipid transporter activity. Predicted to be involved in lipid transport. Predicted to localize to integral component of membrane and intracellular membrane-bounded organelle. |
| DS10_00003400 | N/A | Molecular function: pyruvate carboxylase activity. Biological process: pyruvate metabolic process | 5.67883848730859e-06 | Dmel\PCB Pyruvate carboxylase ([PCB](http://flybase.org/search/PCB)) encodes an enzyme that regulates D-serine levels Exhibits pyruvate carboxylase activity. Predicted to be involved in gluconeogenesis and pyruvate metabolic process. Localizes to mitochondrion |
| DS10_00010856 | Int6 | Molecular function: translation initiation factor activity. Biological process: neuron projection morphogenesis; phagocytosis, engulfment. | 5.74248939370221e-06 | Dmel\eIF3e eukaryotic translation initiation factor 3 subunit e ([eIF3e](http://flybase.org/search/eIF3e)) encodes a component of the translation initiation complex and is a positive regulator of cullin neddylation, which is required for Cullin-ring ubiquitin ligase activity. It also regulates spindle assembly, microtubule dynamics and mitotic progression. |
| DS10_00002078 | N/A | Molecular function is unknown. Biological process is unknown Top nucleotide BLAST:PREDICTED: Drosophila suzukii insulin-like growth factor-binding protein complex acid labile subunit (LOC108010244), mRNA top protein blast: PREDICTED: insulin-like growth factor-binding protein complex acid labile subunit [Drosophila suzukii] | 5.82482064254431e-06 | Dmel\CG5819 Is expressed in several structures, including Malpighian tubule primordium; embryonic Malpighian tubule; embryonic/larval midgut; presumptive embryonic/larval digestive system; and proventriculus primordium. |
| DS10_00012945 | N/A | Molecular function: oxidoreductase activity. Biological process: oxidation-reduction process. | 5.84369107232697e-06 | Dmel\CG6034 Predicted to have polyamine oxidase activity. Predicted to be involved in oxidation-reduction process. Orthologous to human SMOX (spermine oxidase). The Other oxidoreductases, is a collection of oxidoreductases that do not fit into any of the other major oxidoreductases. |
| DS10_00006393 | RpS5a | Molecular function: structural constituent of ribosome. Biological process: mitotic spindle elongation; mitotic spindle organization. | 6.0089990986121e-06 | Dmel\RpS5a Ribosomal protein S5a ([RpS5a](http://flybase.org/search/RpS5a)) encodes a structural constituent of ribosomes. A structural constituent of ribosome. Predicted to be involved in ribosomal small subunit assembly and translation. Localizes to cytoplasm and nucleolus. |
| DS10_00000455 | chico | Molecular function: insulin-like growth factor receptor binding; insulin receptor binding. Biological process: biological regulation; single-organism developmental process; cellular component organization or biogenesis; growth; multicellular organism reproduction; developmental growth; response to stress; regulation of multicellular organismal process; gamete generation; male germ-line stem cell division; associative learning; response to nutrient levels; response to decreased oxygen levels. | 6.10481725159124e-06 | Dmel\chico chico ([chico](http://flybase.org/search/chico)) encodes a substrate of the product of [InR](http://flybase.org/search/InR). As a key component of the Insulin pathway, the [chico](http://flybase.org/search/chico) product plays an essential role in the control of cell size and growth. |
| DS10_00004713 | N/A | Molecular function is unknown. Biological process is unknown Top nucleotide BLAST; PREDICTED: Drosophila suzukii 27 kDa hemolymph protein (LOC108015160), mRNA to protein BLAST: PREDICTED: 27 kDa hemolymph protein [Drosophila suzukii] | 6.13638538477476e-06 | Dmel\CG11378 Is expressed in adult head; anterior midgut primordium; embryonic/larval midgut; and posterior midgut primordium |
| DS10_00006193 | N/A | Molecular function is unknown. Biological process is unknown. To nucleotide BLAST; PREDICTED: Drosophila suzukii uncharacterized LOC108005461 (LOC108005461), mRNA top protein: PREDICTED: uncharacterized protein LOC108005461 [Drosophila suzukii] | 6.17076233677007e-06 | Molecular function is unknown. Biological process is unknown. |
| DS10_00003137 | N/A | Molecular function is unknown. Biological process is unknown. Top nucleotide BLAST; PREDICTED: Drosophila suzukii uncharacterized LOC108009657 (LOC108009657), mRNA top protein: hypothetical protein AWZ03_002443 [Drosophila navojoa] Second result (59% accuracy vs previous 99%): PREDICTED: uncharacterized protein LOC108009657 [Drosophila suzukii] | 6.19883818069463e-06 | Dmel\CG15701 Predicted to contribute to ATP-dependent microtubule motor activity, plus-end-directed. Predicted to be involved in positive regulation of ATP-dependent microtubule motor activity, plus-end-directed and transport along microtubule. |
| DS10_00012993 | N/A | Molecular function: zinc ion binding. Biological process is unknown | 6.23718319686678e-06 | Dmel\Smyd5 SET and MYND domain containing, class 5 Predicted to have S-adenosylmethionine-dependent methyltransferase activity. Predicted to be involved in histone lysine methylation. Predicted to localize to nuclear chromatin The SET and MYND (SMYD) proteins are lysine methyltransferases that catalyse the addition of one or more methyl groups, using S-adenosyl-l-methionine (SAM) as a cofactor, to the amino group of a lysine residue in the substrate resulting in a mono, di, or trimethylated lysine and the cofactor by-product S-adenosyl-l-homocysteine (SAH) |
| DS10_00011232 | N/A | Molecular function is unknown. Biological process is unknown. Top nucleotide BLAST: PREDICTED: Drosophila suzukii basic proline-rich protein (LOC108017318), transcript variant X1, mRNA top protein BLAST: PREDICTED: basic proline-rich protein isoform X1 [Drosophila suzukii] | 6.26048278910265e-06 | Dmel\CG7016 Is expressed in embryonic anal pad; embryonic large intestine; hindgut proper primordium; and large intestine primordium. |
| DS10_00004545 | N/A | Molecular function: hydrolase activity, acting on ester bonds; zinc ion binding. Biological process is unknown | 6.30471972325244e-06 | Dmel\CG9119 Predicted to have hydrolase activity, acting on ester bonds and zinc ion binding activity. Predicted to localize to nucleus. Is expressed in several structures, including cortical zone of lymph gland primary lobe; embryonic Malpighian tubule; embryonic/larval midgut; embryonic/larval midgut primordium; and yolk nucleus |
| DS10_00012210 | N/A | Molecular function: aminopeptidase activity. Biological process: proteolysis. | 6.35478993121234e-06 | Dmel\CG31343 Predicted to have metalloaminopeptidase activity; peptide binding activity; and zinc ion binding activity. Predicted to be involved in peptide catabolic process and proteolysis. Predicted to localize to cytoplasm |
| DS10_00011113 | No results | No results; no top results for nucleotide blast, top protein blast: hypothetical protein TCAL_15025 [Tigriopus californicus] | 6.56871090436712e-06 | No results |
| DS10_00002697 | N/A | Molecular function is unknown. Biological process is unknown Top nucleotide result: PREDICTED: Drosophila suzukii integumentary mucin C.1-like (LOC108008468), mRNA to protein result: PREDICTED: integumentary mucin C.1-like [Drosophila suzukii] | 6.63790087617184e-06 | Dmel\CG30026 |
| DS10_00001045 | Mtp | Molecular function: phosphatidylcholine transporter activity. Biological process: synaptic target recognition; lipoprotein metabolic process. | 6.76571074678629e-06 | Dmel\Mtp Microsomal triacylglycerol transfer protein ([Mtp](http://flybase.org/search/Mtp)) encodes a phosphatidylcholine transporter involved in lipoprotein metabolism, tracheal system lumen formation and neuron development. |
| DS10_00010411 | N/A | Molecular function: sodium:iodide symporter activity. Biological process: transmembrane transport | 6.88364490208114e-06 | Dmel\CG6723 Predicted to have transmembrane transporter activity. Predicted to be involved in transmembrane transport. Predicted to localize to membrane. |
| DS10_00004989 | N/A | Molecular function: transferase activity, transferring phosphorus-containing groups. Biological process is unknown. | 6.94054227098288e-06 | Dmel\CG2004 Is expressed in adult head. |
| DS10_00004892 | Cht6 | Molecular function: chitinase activity. Biological process: chitin catabolic process; carbohydrate metabolic process. | 6.97150698095661e-06 | Dmel\Cht6 Chitinase 6 ([Cht6](http://flybase.org/search/Cht6)) encodes an enzyme involved in ecdysis. |
| DS10_00012588 | N/A | Molecular function: nucleotide binding; oxidoreductase activity. Biological process: oxidation-reduction process. | 6.97023597613765e-06 | Dmel\Sccpdh2 Saccheropin dehydrogenase 2 Predicted to have oxidoreductase activity. Predicted to be involved in glycolipid biosynthetic process. Predicted to localize to lipid droplet; mitochondrion; and plasma membrane |
| DS10_00000203 | N/A | Molecular function: G-protein coupled receptor activity. Biological process is unknown. | 7.02566344583044e-06 | Is expressed in adult head. Dmel\CG4168 |
| DS10_00012658 | N/A | Molecular function is unknown. Biological process is unknown. | 7.05192279595501e-06 | Dmel\CG8642 Predicted to have G protein-coupled receptor binding activity and chitin binding activity. Predicted to be involved in G protein-coupled receptor signaling pathway |
| DS10_00004155 | N/A | Molecular function: secondary active organic cation transmembrane transporter activity. Biological process: transmembrane transport. | 7.08235271850928e-06 | Dmel\CG42269 Predicted to have transmembrane transporter activity. Predicted to be involved in transmembrane transport. Predicted to localize to integral component of membrane. |
| DS10_00010768 | nAcRbeta-96A | Molecular function: acetylcholine-activated cation-selective channel activity. Biological process: ion transport. | 7.09964032675432e-06 | Dmel\nAChRβ2 nicotinic Acetylcholine Receptor β2 ([nAChRβ2](http://flybase.org/search/nAChR%CE%B22)) encodes a subunit of the nicotinic acetylcholine receptor, which is part of the superfamily of ligand-gated ion channels. These channels open to facilitate the passage of cations across synaptic membranes in response to the binding of their native ligand, acetylcholine. Mutations in [nAChRβ2](http://flybase.org/search/nAChR%CE%B22) have been associated with resistance phenotypes to several neonicotinoid insecticides |
| DS10_00000677 | RpL27A | Molecular function: structural constituent of ribosome. Biological process: mitotic spindle elongation; mitotic spindle organization; centrosome duplication. | 7.15298516118583e-06 | Dmel\RpL27A Ribosomal protein L27A ([RpL27A](http://flybase.org/search/RpL27A)) encodes a ribosomal structural constituent |
| DS10_00001675 | RpL24 | Molecular function: structural constituent of ribosome. Biological process: mitotic spindle elongation; mitotic spindle organization; centrosome duplication. | 7.26679160840267e-06 | Dmel\RpL24 A structural constituent of ribosome. Predicted to be involved in assembly of large subunit precursor of preribosome; ribosomal large subunit assembly; and translation. |
| DS10_00002169 | N/A | Molecular function: glucose transmembrane transporter activity. Biological process: transmembrane transport. | 7.33047491065988e-06 | Dmel\CG4797 Predicted to have carbohydrate:proton symporter activity. Predicted to be involved in glucose import. Predicted to localize to integral component of plasma membrane. Is expressed in adult fat body and adult head. |
| DS10_00003785 | dally | Molecular function: protein binding. Biological process: single-organism developmental process; biological regulation; post-embryonic organ morphogenesis; sensory organ development; cellular component organization or biogenesis; regulation of developmental process; regulation of multicellular organismal process; system development; cell cycle; regulation of BMP signaling pathway; neuron differentiation. | 7.41082073383254e-06 | Dmel\dally division abnormally delayed ([dally](http://flybase.org/search/dally)) encodes a core protein of heparan sulfate proteoglycans of the glypican family. It acts as a co-receptor for growth factors and morphogens, such as the products of [dpp](http://flybase.org/search/dpp), [wg](http://flybase.org/search/wg), [hh](http://flybase.org/search/hh), [upd1](http://flybase.org/search/upd1) and FGFs, affecting signaling and distribution of these ligands. The roles of the product of [dally](http://flybase.org/search/dally) include wing development and germline stem cell maintenance. |
| DS10_00012481 | RpL34b | Molecular function: structural constituent of ribosome. Biological process: translation. | 7.43103727305825e-06 | Dmel\RpL34b Ribosomal protein L34b ([RpL34b](http://flybase.org/search/RpL34b)) encodes a ribosomal structural constituent |
| DS10_00006397 | N/A | Molecular function: calcium ion binding; phospholipase A2 activity. Biological process: lipid catabolic process; phospholipid metabolic process. | 7.50551544382776e-06 | Dmel\CG42237 Predicted to have calcium-dependent phospholipase A2 activity. Predicted to be involved in arachidonic acid secretion and phospholipid metabolic process. |
| DS10_00008154 | Pngl | Molecular function: carbohydrate binding. Biological process: glycoprotein catabolic process. | 7.5036741517853e-06 | Dmel\Pngl Exhibits carbohydrate binding activity and peptide-N4-(N-acetyl-beta-glucosaminyl)asparagine amidase activity. Involved in positive regulation of BMP signaling pathway; protein deglycosylation; and visceral mesoderm-endoderm interaction involved in midgut development. Localizes to cytosol. |
| DS10_00010732 | N/A | Molecular function is unknown. Biological process is unknown. Top nucelotide:PREDICTED: Drosophila suzukii uncharacterized LOC108016408 (LOC108016408), mRNA top protein:PREDICTED: uncharacterized protein LOC108016408 [Drosophila suzukii] | 7.50189713620129e-06 | Dmel\CG43179 |
| DS10_00005842 | RpLP2 | Molecular function: structural constituent of ribosome. Biological process: translation | 7.56043985938494e-06 | Dmel\RpLP2 A structural constituent of ribosome. Predicted to be involved in cytoplasmic translation. Localizes to cytosolic ribosome. Is expressed in adult head and adult heart |
| DS10_00000003 | Sam-S | Molecular function: methionine adenosyltransferase activity. Biological process: chaeta development; wing disc development; S-adenosylmethionine biosynthetic process. | 7.64929089802124e-06 | Dmel\Sam-S Predicted to have methionine adenosyltransferase activity. Involved in S-adenosylmethionine biosynthetic process and determination of adult lifespan. Predicted to localize to cytosol. Human ortholog(s) of this gene implicated in hypermethioninemia. |
| DS10_00002723 | Bap55 | Molecular function: transcription coactivator activity. Biological process: positive regulation of gene silencing by miRNA; dendrite morphogenesis; mitosis; cytokinesis; muscle organ development; positive regulation of transcription, DNA-dependent; dendrite guidance. | 7.66316318090589e-06 | Dmel\Bap55 Brahma associated protein 55kD ([Bap55](http://flybase.org/search/Bap55)) encodes a member of two chromatin remodeling complexes. As part of the Brahma complex, it is needed for cell growth and survival in the wing imaginal disc; as a member of the TIP60 complex, it is thought to regulate dendrite wiring specificity in olfactory projection neurons The Drosophila olfactory system exhibits very precise and stereotyped wiring that is specified predominantly by genetic programming. Dendrites of olfactory projection neurons (PNs) pattern the developing antennal lobe before olfactory receptor neuron axon arrival, indicating an intrinsic wiring mechanism for PN dendrites. These wiring decisions are likely determined through a transcriptional program.We find that loss of Brahma associated protein 55 kD (Bap55) results in a highly specific PN mistargeting phenotype. In Bap55 mutants, PNs that normally target to the DL1 glomerulus mistarget to the DA4l glomerulus with 100% penetrance. Loss of Bap55 also causes derepression of a GAL4 whose expression is normally restricted to a small subset of PNs. Bap55 is a member of both the Brahma (BRM) and the Tat interactive protein 60 kD (TIP60) ATP-dependent chromatin remodeling complexes. The Bap55 mutant phenotype is partially recapitulated by Domino and Enhancer of Polycomb mutants, members of the TIP60 complex. However, distinct phenotypes are seen in Brahma and Snf5-related 1 mutants, members of the BRM complex. The Bap55 mutant phenotype can be rescued by postmitotic expression of Bap55, or its human homologs BAF53a and BAF53b.Our results suggest that Bap55 functions through the TIP60 chromatin remodeling complex to regulate dendrite wiring specificity in PNs. The specificity of the mutant phenotypes suggests a position for the TIP60 complex at the top of a regulatory hierarchy that orchestrates dendrite targeting decisions. |
| DS10_00011151 | Pgm | Molecular function: phosphoglycerate mutase activity; phosphoglucomutase activity. Biological process: lateral inhibition; glycogen biosynthetic process; flight. | 7.75249619907871e-06 | Dmel\Pgm1 Exhibits phosphoglucomutase activity. Involved in glycogen biosynthetic process. Predicted to localize to cytosol. |
| DS10_00008942 | N/A | Molecular function is unknown. Biological process is unknown. Top nucleotide BLAST: PREDICTED: Drosophila suzukii spermine oxidase (LOC108010501), mRNA to protein BLAST: PREDICTED: uncharacterized protein LOC108010496 [Drosophila suzukii] | 7.99927217184354e-06 | Dmel\CG32181 |
| DS10_00010677 | No results | No results | 8.16498475252e-06 | No results |
| DS10_00012656 | Obp83g | Molecular function: odorant binding. Biological process: sensory perception of chemical stimulus | 8.15613381973254e-06 | Dmel\Obp83g Predicted to have odorant binding activity. Predicted to be involved in sensory perception of smell. Predicted to localize to extracellular region. Odorant Binding Proteins (OBP) are small (10 to 30 kDa) proteins secreted by auxiliary cells surrounding the olfactory receptor neurons. OBPs are characterized by a specific protein domain that comprises six α-helices joined by three disulfide bonds. Although the full function of the OBPs is not well established, it is believed that they may act as molecular carriers that transport odorants and deliver them to the olfactory receptors located on the sensory neurons. |
| DS10_00004268 | N/A | Molecular function: oxidoreductase activity. Biological process: oxidation-reduction process. | 8.43270916239108e-06 | Dmel\CG10512 |
| DS10_00000591 | N/A | Molecular function: glycoprotein-N-acetylgalactosamine 3-beta-galactosyltransferase activity. Biological process: O-glycan processing. | 8.55854176257697e-06 | Dmel\CG18558 N-acetylgalactosaminide beta-1,3-galactosyltransferase Predicted to have glycoprotein-N-acetylgalactosamine 3-beta-galactosyltransferase activity. Predicted to be involved in O-glycan processing, core 1. Predicted to localize to membrane |
| DS10_00008529 | N/A | Molecular function: 4-hydroxybutyrate CoA-transferase activity. Biological process: neurogenesis | 8.61459868269792e-06 | Dmel\CG7920 Predicted to have catalytic activity. Predicted to be involved in acetyl-CoA metabolic process. Localizes to cytosol and mitochondrion. |
| DS10_00012000 | Srp72 | Molecular function: 7S RNA binding. Biological process: dsRNA transport. | 9.13317999644763e-06 | Dmel\Srp72 Signal recognition particle 72 Predicted to contribute to ribosome binding activity. Involved in dsRNA transport. Predicted to localize to signal recognition particle, endoplasmic reticulum targeting. Is |
| DS10_00012464 | FK506-bp1 | Molecular function: protein binding; juvenile hormone response element binding; FK506 binding. Biological process: negative regulation of macroautophagy; juvenile hormone mediated signaling pathway; negative regulation of autophagy | 9.30053108983493e-06 | Dmel\Fkbp39 FK506-binding protein 39kD ([Fkbp39](http://flybase.org/search/Fkbp39)) encodes a protein thay acts as an inhibitor of autophagy in larval fat body. |
| DS10_00006178 | N/A | Molecular function: catalytic activity. Biological process is unknown. | 9.40152610787729e-06 | Dmel\CG34015 Predicted to have catalytic activity. Is expressed in adult head. |
| DS10_00008724 | Tm2 | Molecular function: actin binding. Biological process: heart development. | 9.42311772852991e-06 | Dmel\Tm2 Tropomyosin 2 Predicted to have actin filament binding activity. Involved in heart development and positive regulation of sarcomere organization. Predicted to localize to actin filament. |
| DS10_00007954 | Scp2 | Molecular function: GTPase activity. Biological process is unknown. | 9.59154592386409e-06 | Dmel\Scp2 Sarcoplasmic calcium-binding protein 2 Exhibits GTPase activity. Predicted to localize to endoplasmic reticulum. |
| DS10_00006650 | N/A | Molecular function: glutaminase activity. Biological process: imaginal disc-derived wing morphogenesis. | 9.81253149242154e-06 | Dmel\GLS Glutaminase Predicted to have glutaminase activity. Predicted to be involved in glutamate biosynthetic process and glutamine catabolic process. Predicted to localize to mitochondrion. |
| DS10_00008623 | N/A | Molecular function: sodium:iodide symporter activity. Biological process: transmembrane transport. | 1.01068796692218e-05 | Dmel\CG6723 Predicted to have transmembrane transporter activity. Predicted to be involved in transmembrane transport. Predicted to localize to membrane. |
| DS10_00009594 | N/A | Molecular function: glycerol-3-phosphate O-acyltransferase activity. Biological process: plasma membrane organization; phospholipid biosynthetic process. | 1.01386337335367e-05 | Dmel\mino minotaur ([mino](http://flybase.org/search/mino)) encodes a member of the conserved glycerol-3-phosphate O-acyltransferase family, which converts glycerol-3-phosphate into lysophosphatidic acid. It functions in phosphatidic acid biosynthesis. It is also essential for piRNA biogenesis in gonads, with [mino](http://flybase.org/search/mino) mutants showing transposon de-repression and sterility phenotypes. |
| DS10_00011587 | RpL13 | Molecular function: structural constituent of ribosome. Biological process: mitotic spindle elongation; mitotic spindle organization; centrosome duplication. | 1.01304869598691e-05 | Dmel\RpL13 Ribosomal protein L13 ([RpL13](http://flybase.org/search/RpL13)) encodes a ribosomal structural constituent |
| DS10_00003865 | path | Molecular function: amino acid transmembrane transporter activity. Biological process: growth | 1.03596678167654e-05 | Dmel\path pathetic Exhibits amino acid transmembrane transporter activity. Involved in several processes, including neuron projection morphogenesis; regulation of cell projection size; and regulation of imaginal disc-derived wing size. Localizes to several cellular components, including dendrite; endolysosome; and neuronal cell body. |
| DS10_00012334 | GNBP1 | Molecular function: protein binding; peptidoglycan binding. Biological process: immune response; peptidoglycan catabolic process; defense response to Gram-positive bacterium. | 1.06125863726177e-05 | Dmel\GNBP1 Gram-negative bacteria binding protein 1 ([GNBP1](http://flybase.org/search/GNBP1)) encodes a hemolymphatic protein that participates together with the product of [PGRP-SA](http://flybase.org/search/PGRP-SA) in the activation of the Toll pathway by peptidoglycans, a cell-wall component of bacteria. |
| DS10_00000262 | qtc | Molecular function is unknown. Biological process: lateral inhibition; mating behavior, sex discrimination; male courtship behavior. | 1.06927010840478e-05 | Dmel\qtc quick-to-court ([qtc](http://flybase.org/search/qtc)) encodes an alpha-helical coiled-coiled protein whose mutants exhibit altered male courtship behavior |
| DS10_00008390 | N/A | Molecular function is unknown. Biological process is unknown. | 1.08202814699661e-05 | Dmel\Nna1 Nna1 carboxypeptidase ([Nna1](http://flybase.org/search/Nna1)) encodes a highly conserved cytoplasmic carboxypeptidase. [Nna1](http://flybase.org/search/Nna1) loss leads to neurodegeneration by causing mitochondrial dysfunction. It has been proposed to function as protein-deglutamylases, possibly interacting with and modifying tubulin. [ |
| DS10_00009456 | N/A | Molecular function: selenium binding. Biological process is unknown. | 1.10737163754102e-05 | Dmel\CG7966 redicted to have selenium binding activity. Is expressed in adult head |
| DS10_00012845 | N/A | Molecular function: N-acetylgalactosamine-4-sulfatase activity. Biological process: metabolic process. | 1.12784712083218e-05 | Dmel\CG7402 Predicted to have sulfuric ester hydrolase activity. |
| DS10_00006565 | Vmat | Molecular function: synaptic vesicle amine transmembrane transporter activity; monoamine transmembrane transporter activity. Biological process: dopamine transport; monoamine transport; histamine transport; synaptic vesicle amine transport. | 1.1652328134727e-05 | Dmel\Vmat Vesicular monoamine transporter ([Vmat](http://flybase.org/search/Vmat)) encodes a protein responsible for packaging the neurotransmitters dopamine, serotonin and octopamine into secretory vesicles |
| DS10_00000213 | UK114 | Molecular function is unknown. Biological process: protein folding. | 1.16977433963149e-05 | Dmel\UK114 Predicted to have deaminase activity. Involved in protein folding. Predicted to localize to cytosol and mitochondrion. Molecular chaperone. Seems to fulfill an ATP-independent, HSP70-like function in protein folding. May protect essential factors of cell proliferation during heat shock. No role in calpain activation. |
| DS10_00012408 | twr | Molecular function: peptidase activity. Biological process: signal peptide processing | 1.17368480292573e-05 | Dmel/twr twisted bristles roughened eye Predicted to have peptidase activity. Predicted to be involved in signal peptide processing. Localizes to endomembrane system. Most alleles lethal; few escapers display twisted bristles and rough eyes; survivors female sterile. |
| DS10_00000602 | colt | Molecular function: transporter activity; carnitine:acyl carnitine antiporter activity. Biological process: phagocytosis, engulfment. | 1.180027142603e-05 | Dmel\colt Predicted to have carnitine:acyl carnitine antiporter activity. Involved in epithelial cell morphogenesis and liquid clearance, open tracheal system. Predicted to localize to integral component of membrane and mitochondrial inner membrane. |
| DS10_00000353 | Akap200 | Molecular function: protein kinase A binding. Biological process: regulation of establishment of planar polarity; negative regulation of Ras protein signal transduction; autophagic cell death; behavioral response to ethanol; salivary gland cell autophagic cell death. | 1.22860054153872e-05 | Dmel\Akap200 A kinase anchor protein 200 ([Akap200](http://flybase.org/search/Akap200)) encodes a scaffolding protein that contributes to the spatial and temporal regulation of the Protein Kinase A holoenzyme. It regulates the Notch signaling and the organization of the actin cytoskeleton |
| DS10_00008675 | N/A | Molecular function is unknown. Biological process is unknown. | 1.2290952908339e-05 | Dmel\Sec20 Predicted to have SNAP receptor activity. Predicted to be involved in endoplasmic reticulum membrane fusion. Predicted to localize to SNARE complex and endoplasmic reticulum. Q-SNAREs are members of the SNARE (soluble N-ethylmaleimide-sensitive factor-attachment protein (SNAP) receptor) family that mediate vesicle-fusion. SNAREs are cytoplasmic, membrane-anchored proteins that contain a 60 amino acid SNARE domain. Three Q-SNAREs domains from the target membrane and one R-SNARE domain from the vesicle interact to form a four four-helix bundle that pulls the two membranes in close proximity and promotes fusion. After fusion, the complexes are dissembled by NSF (N-ethylmaleimide-sensitive factor) and SNAPs (Soluble NSF Attachment Proteins). Q-SNAREs are named from their contribution of a conserved glutamine (Q) residue to the helix bundle. Q SNARE domains are designated a, b or c, depending on their position within the helix bundle. |
| DS10_00009442 | CecA2 | Molecular function is unknown. Biological process: defense response to Gram-negative bacterium; humoral immune response; defense response to Gram-positive bacterium; antibacterial humoral response. | 1.24486592363293e-05 | Dmel\CecA2 Cecropin A2 ([CecA2](http://flybase.org/search/CecA2)) encodes an antibacterial peptide with activity against Gram-negative bacteria. It is expressed in the fat body during the systemic immune response and in various epithelia, and it is regulated at the transcriptional level by the immune deficiency pathway. |
| DS10_00010256 | AP-2mu | Molecular function: protein transporter activity. Biological process: dsRNA transport; endocytosis. | 1.24427013585155e-05 | Dmel\AP-2μ Adaptor Protein complex 2, μ subunit ([AP-2μ](http://flybase.org/search/AP-2%CE%BC)) encodes a component of the AP-2 adaptor complex, which recruits certain transmembrane proteins into clathrin-coated pits for endocytic internalization |
| DS10_00013518 | RpL27 | Molecular function: structural constituent of ribosome. Biological process: centrosome organization; mitotic spindle elongation; mitotic spindle organization; centrosome duplication. | 1.26766126279168e-05 | Dmel\RpL27 Predicted to be a structural constituent of ribosome. Predicted to be involved in cytoplasmic translation. Predicted to localize to cytosolic large ribosomal subunit. |
| DS10_00011805 | Indy | Molecular function: citrate transmembrane transporter activity; succinate transmembrane transporter activity. Biological process: regulation of sequestering of triglyceride; determination of adult lifespan. | 1.28679519886463e-05 | Dmel\Indy I'm not dead yet ([Indy](http://flybase.org/search/Indy)) encodes a plasma membrane transporter of Krebs cycle intermediates (citrate, succinate, fumarate, alpha-ketoglutarate) of the SLC13A family that is important in organismal intermediary metabolism. Mutations in [Indy](http://flybase.org/search/Indy) create a favorable metabolic state, similar to calorie restriction, and extend life span. |
| DS10_00000253 | TpnC25D | Molecular function: calcium ion binding. Biological process is unknown. | 1.30609679331421e-05 | Dmel\TpnC25D Predicted to have calcium ion binding activity and enzyme regulator activity. Predicted to be involved in calcium-mediated signaling and microtubule cytoskeleton organization. |
| DS10_00003802 | Galk | Molecular function: galactokinase activity. Biological process: galactose metabolic process; carbohydrate phosphorylation. | 1.32570528964179e-05 | Dmel\Galk Galactokinase ([Galk](http://flybase.org/search/Galk)) encodes a protein involved in galactose metabolism that regulates calcineurin-induced cardiac enlargement. |
| DS10_00003167 | N/A | Molecular function is unknown. Biological process is unknown. | 1.37163989259345e-05 | Dmel\CG8399 Ferric-chelate reductase (NADH) Exhibits ferric-chelate reductase activity. Involved in oxidation-reduction process. Localizes to integral component of membrane. Metal ion oxidoreductases with NAD or NADP as acceptor, include dehydrogenases that oxidize metal ions and contribute to the increase in their oxidation state with the reduction of NAD+ or NADP+. Putative ferric-chelate reductases reduce Fe(3+) to Fe(2+) before its transport from the endosome to the cytoplasm. |
| DS10_00003602 | N/A | Molecular function is unknown. Biological process is unknown. Top protein BLAST: PREDICTED: protein CDV3 homolog isoform X1 [Drosophila suzukii]top nucleotide BLAST: PREDICTED: Drosophila suzukii protein CDV3 homolog (LOC108009961), transcript variant X1, mRNA | 1.3850780550858e-05 | Dmel\CG3760 |
| DS10_00001965 | N/A | Molecular function: serine-type endopeptidase inhibitor activity. Biological process: multicellular organism reproduction | 1.39369373715593e-05 | Dmel\CG44008 Predicted to have serine-type endopeptidase inhibitor activity. Involved in multicellular organism reproduction. |
| DS10_00009088 | N/A | Molecular function: tRNA binding; translation initiation factor activity; ribosome binding. Biological process: regulation of translation; ribosome assembly. | 1.40821347984795e-05 | Dmel\CG7414 Predicted to have ribosome binding activity; tRNA binding activity; and translation initiation factor activity. Predicted to be involved in regulation of translation Functions in the early steps of protein synthesis of a small number of specific mRNAs. Acts by directing the binding of methionyl-tRNAi to 40S ribosomal subunits. In contrast to the eIF-2 complex, it binds methionyl-tRNAi to 40S subunits in a codon-dependent manner, whereas the eIF-2 complex binds methionyl-tRNAi to 40S subunits in a GTP-dependent manner. |
| DS10_00007040 | sws | Molecular function: protein binding; lysophospholipase activity. Biological process: membrane lipid metabolic process; phosphatidylcholine metabolic process; cellular membrane organization; sensory perception of smell; neuron apoptotic process. | 1.41703053894767e-05 | DMel/sws  swiss cheese ([sws](http://flybase.org/search/sws)) encodes a transmembrane protein that hydrolyzes phosphatidylcholine and binds to and inhibits the C3 catalytic subunit of protein kinase A. Its loss leads to neuronal and glial degeneration and apoptotic cell death. |
| DS10_00006586 | AttC | Molecular function is unknown. Biological process: humoral immune response; defense response to Gram-positive bacterium; defense response; antibacterial humoral response. | 1.45555511404416e-05 | Dmel\AttC Attacin-C ([AttC](http://flybase.org/search/AttC)) encodes an immune inducible peptide that shows homology to antibacterial peptides having activity against Gram-negative bacteria. |
| DS10_00006990 | No results | No results Top nucleotide BLAST: PREDICTED: Drosophila suzukii basic proline-rich protein (LOC108004612), mRNA top protein BLAST: PREDICTED: basic proline-rich protein [Drosophila suzukii] | 1.45503045489702e-05 | No results |
| DS10_00012811 | N/A | Molecular function: 2 iron, 2 sulfur cluster binding; iron ion binding; electron carrier activity; oxidoreductase activity, acting on CH-OH group of donors; flavin adenine dinucleotide binding. Biological process: oxidation-reduction process. | 1.45566911591617e-05 | Dmel\AOX2 Aldehyde oxidase 2 ([AOX2](http://flybase.org/search/AOX2)) encodes a pyridoxal oxidase involved in pyridoxal metabolism |
| DS10_00011698 | N/A | Molecular function: threonine aldolase activity. Biological process: cellular amino acid metabolic process | 1.45968494565102e-05 | Dmel\CG10184 Predicted to have L-allo-threonine aldolase activity. Predicted to be involved in glycine biosynthetic process and threonine catabolic process. |
| DS10_00000518 | N/A | Molecular function: metalloendopeptidase activity. Biological process: proteolysis | 1.47440655893312e-05 | Dmel\Nepl9 Predicted to have metalloendopeptidase activity. Predicted to be involved in proteolysis.The Neprilysin-like metallopeptidases belong to the M13 family of metalloproteases. They show significant similarity to vertebrate and Drosophila Neprilysins but lack one or more of the conserved motifs important for catalytic activity, indicating they are likely inactive peptidases |
| DS10_00008139 | vn | Molecular function: heparin binding; epidermal growth factor receptor binding. Biological process: single-organism developmental process; system development; post-embryonic organ morphogenesis; appendage development; biological regulation; positive regulation of cell proliferation; cellular component organization or biogenesis; localization; cellular process; cell projection assembly; cellular component movement; cell fate commitment; digestive tract development; notum development. | 1.50467635487973e-05 | Dmel\vn vein ([vn](http://flybase.org/search/vn)) encodes a secreted neuregulin-like EGFR ligand. It has weaker intrinsic activity, and a lower receptor affinity, than the TGFalpha-like ligand encoded by [spi](http://flybase.org/search/spi). It has roles in growth and patterning of tissues including muscle, midgut, ovary, trachea, glia, eye, leg, and wing. |
| DS10_00006754 | Cyp4e2 | Molecular function: electron carrier activity. Biological process: oxidation-reduction process. | 1.51917542428836e-05 | Dmel\Cyp4e2 Predicted to have heme binding activity; iron ion binding activity; and oxidoreductase activity, acting on paired donors, with incorporation or reduction of molecular oxygen. Predicted to be involved in oxidation-reduction process |
| DS10_00008673 | N/a | Molecular function: glucose transmembrane transporter activity. Biological process: transmembrane transport. | 1.63292433286074e-05 | Dmel\CG1213 Predicted to have carbohydrate:proton symporter activity. Predicted to be involved in glucose import. Predicted to localize to integral component of plasma membrane. |
| DS10_00002242 | Egfr | Molecular function: protein binding. Biological process: single-organism developmental process; biological regulation; sensory organ development; system development; post-embryonic organ morphogenesis; regulation of developmental process; digestive tract development; cellular component organization or biogenesis; cell fate commitment; open tracheal system development. | 1.64379968339838e-05 | Dmel\Egfr Epidermal growth factor receptor ([Egfr](http://flybase.org/search/Egfr)) encodes the transmembrane tyrosine kinase receptor for signaling ligands (encoded by [grk](http://flybase.org/search/grk), [spi](http://flybase.org/search/spi), [vn](http://flybase.org/search/vn), and [Krn](http://flybase.org/search/Krn)) in the TGFα family, which utilises the intracellular MAP kinase pathway. The product of [Egfr](http://flybase.org/search/Egfr) contributes to growth regulation, cell survival and developmental patterning. |
| DS10_00007582 | N/A | Molecular function: beta-mannosidase activity. Biological process: carbohydrate metabolic process. | 1.65185035893411e-05 | Dmel\β-Man Predicted to have beta-mannosidase activity. Predicted to be involved in glycoprotein catabolic process. Predicted to localize to lysosome. |
| DS10_00011124 | dnd | Molecular function: GTPase activity; GTP binding. Biological process: branch fusion, open tracheal system. | 1.68766809363669e-05 | Dmel\dnd dead end ([dnd](http://flybase.org/search/dnd)) encodes an Arf-like3 GTPase that controls the targeting of exocytosis machinery to specific apical domains in fusion cells during the tracheal branch fusion process. |
| DS10_00003515 | N/A | Molecular function is unknown. Biological process is unknown. Top nucleotide BLAST: PREDICTED: Drosophila suzukii tetratricopeptide repeat protein 25 (LOC108008747), transcript variant X1, mRNA top protein BLAST: PREDICTED: tetratricopeptide repeat protein 25 isoform X1 [Drosophila suzukii] | 1.69750131608369e-05 | Dmel\CG13502 Predicted to localize to cytoplasm. Human ortholog(s) of this gene implicated in primary ciliary dyskinesia 35. Orthologous to human TTC25 (tetratricopeptide repeat domain 25). |
| DS10_00004692 | N/A | Molecular function: ubiquitin thiolesterase activity; zinc ion binding. Biological process: ubiquitin-dependent protein catabolic process. | 1.71849661740878e-05 | Dmel\Usp16-45 Ubiquitin specific protease 16/45 Predicted to have cysteine-type endopeptidase activity and thiol-dependent ubiquitin-specific protease activity. Predicted to be involved in protein deubiquitination. |
| DS10_00004162 | loj | Molecular function is unknown. Biological process: oviposition; regulation of post-mating oviposition; reproduction | 1.72854227905767e-05 | Dmel\loj logjam ([loj](http://flybase.org/search/loj)) encodes a p24-transporter-type protein involved in oviposition |
| DS10_00003228 | N/A | Molecular function: very-long-chain-acyl-CoA dehydrogenase activity. Biological process: oxidation-reduction process. | 1.73296865609088e-05 | Dmel\CG7461 Predicted to have fatty-acyl-CoA binding activity and very-long-chain-acyl-CoA dehydrogenase activity. Predicted to be involved in oxidation-reduction process. Predicted to localize to mitochondrion. Acyl-CoA dehydrogenases are oxidoreductases that oxidize acyl-CoA with the reduction of electron-transfer flavoprotein. They catalyze the first reaction of mitochondrial fatty acid beta-oxidation. |
| DS10_00002582 | N/A | Molecular function is unknown. Biological process is unknown. Top nucleotuide BLAST:PREDICTED: Drosophila suzukii probable cytochrome P450 6t3 (LOC108009230), mRNA top protein BLAST: uncharacterized protein LOC6546887 [Drosophila erecta] | 1.85954471857518e-05 | Dmel\CG33964 Orthologous to human METTL25 (methyltransferase like 25). |
| DS10_00006960 | RpL36 | Molecular function: structural constituent of ribosome. Biological process: translation. | 1.86639073827045e-05 | Dmel\RpL36 A structural constituent of ribosome. Predicted to be involved in cytoplasmic translation. Localizes to cytoplasm and nucleolus. Colocalizes with nuclear chromosome. |
| DS10_00007141 | No results | No results Top nucleotide BLAST: PREDICTED: Drosophila suzukii protein pangolin, isoforms A/H/I/S (LOC108020361), transcript variant X2, mRNtop protein BLAST: PREDICTED: protein pangolin isoform X1 [Drosophila suzukii] | 1.8632641802285e-05 | No results |
| DS10_00003823 | Slc45-1 | Molecular function: sucrose transmembrane transporter activity. Biological process: sucrose transport. | 1.88126578529065e-05 | Dmel\Slc45-1 Solute carrier family 45 member 1 ([Slc45-1](http://flybase.org/search/Slc45-1)) encodes a proton/sucrose symporter occuring in apical membranes of the late embryonic hindgut as well as in vesicular membranes of ovarian follicle cells. It may either serve as an osmolyte transporter or be responsible for the resorption of sucrose. |
| DS10_00008651 | N/A | Molecular function: carboxylesterase activity. Biological process is unknown | 1.88651029062427e-05 | Dmel\CG4757 Carboxylesterase Predicted to have carboxylic ester hydrolase activity. Predicted to localize to extracellular space. Is expressed in adult head. inferred from biological aspect of ancestor with [PANTHER:PTN000168392](http://www.pantree.org/node/annotationNode.jsp?id=PTN000168392)  (assigned by GO_Central ) |
| DS10_00007868 | N/A | Molecular function: translation initiation factor activity. Biological process: cellular response to hypoxia. | 1.90616239830996e-05 | Dmel\eIF1 eukaryotic translation initiation factor 1 Predicted to have ribosomal small subunit binding activity and translation initiation factor activity. Involved in cellular response to hypoxia. Predicted to localize to eukaryotic 43S preinitiation complex and multi-eIF complex. Is expressed in organism |
| DS10_00001226 | N/A | Molecular function: selenocysteine methyltransferase activity. Biological process is unknown | 1.91837807100809e-05 | Dmel\CG10621 Homocysteine S-methyltransferase Predicted to have S-adenosylmethionine-homocysteine S-methyltransferase activity. Predicted to be involved in S-methylmethionine cycle and methionine biosynthetic process |
| DS10_00012158 | N/A | Molecular function is unknown. Biological process is unknown. Top protein BLAST: PREDICTED: uncharacterized protein LOC108014111 [Drosophila suzukii] top nucleotide BLAST: PREDICTED: Drosophila suzukii uncharacterized LOC108014111 (LOC108014111), mRNA | 1.98525067953227e-05 | Dmel\CG1979 Is expressed in adult head and spermatozoon. |
| DS10_00009937 | N/A | Molecular function: serine-type endopeptidase activity. Biological process: proteolysis | 1.99328857445761e-05 | Dmel\CG40160 Predicted to have serine-type endopeptidase activity. Predicted to be involved in proteolysis |
| DS10_00001349 | nrv3 | Molecular function: sodium:potassium-exchanging ATPase activity. Biological process: potassium ion transport; sodium ion transport. | 1.99689298071255e-05 | Dmel\nrv3 nervana 3 ([nrv3](http://flybase.org/search/nrv3)) encodes one of three beta subunits of the sodium-potassium pump (ATPalpha). [nrv3](http://flybase.org/search/nrv3) is expressed in the nervous system, including subsets of the CNS and Johnston's organ neurons, and is required for hearing |
| DS10_00007886 | N/A | Molecular function: tRNA methyltransferase activity. Biological process: tRNA modification; methionine metabolic process | 2.01275256033367e-05 | Dmel\CG32281 Predicted to have tRNA (guanine-N1-)-methyltransferase activity. Predicted to be involved in mitochondrial tRNA methylation and tRNA N1-guanine methylation. Predicted to localize to mitochondrial matrix. |
| DS10_00012284 | GNBP1 | Molecular function: protein binding; peptidoglycan binding. Biological process: immune response; peptidoglycan catabolic process; defense response to Gram-positive bacterium. | 2.03649260766631e-05 | Dmel\GNBP1 Gram-negative bacteria binding protein 1 ([GNBP1](http://flybase.org/search/GNBP1)) encodes a hemolymphatic protein that participates together with the product of [PGRP-SA](http://flybase.org/search/PGRP-SA) in the activation of the Toll pathway by peptidoglycans, a cell-wall component of bacteria |
| DS10_00010723 | No results | NO results Top protein BL:AST: PREDICTED: uncharacterized protein LOC108014610 isoform X2 [Drosophila suzukii] Top NucletodieBLASTL PREDICTED: Drosophila suzukii uncharacterized LOC108014610 (LOC108014610), transcript variant X2, mRNA | 2.04785687500779e-05 | No results |
| DS10_00000868 | N/A | Molecular function is unknown. Biological process is unknown. Top nucleotide BLAST: PREDICTED: Drosophila suzukii uncharacterized LOC108021544 (LOC108021544), mRNA top protein BLAST: PREDICTED: uncharacterized protein LOC108021543 [Drosophila suzukii] | 2.07850328685785e-05 | Dmel\CG15293 Localizes to extracellular region. Is expressed in adult head; adult heart; and embryonic/larval hemolymph |
| DS10_00007590 | Gel | Molecular function: actin binding. Biological process: phagocytosis, engulfment. | 2.0796700501742e-05 | Dmel\Gel Gelsolin Predicted to have actin filament binding activity. Predicted to be involved in actin filament polymerization. Localizes to actin filament; cytosol; and extracellular region. Calcium-regulated, actin-modulating protein that binds to the plus (or barbed) ends of actin monomers or filaments, preventing monomer exchange (end-blocking or capping). It can promote the assembly of monomers into filaments (nucleation) as well as sever filaments already formed. |
| DS10_00003349 | N/a | Molecular function: GTP binding. Biological process is unknown. | 2.09672602955795e-05 | Dmel\Non1 Novel nucleolar protein 1Predicted to have GTP binding activity. Localizes to nucleolus. Involved in the biogenesis of the 60S ribosomal subunit (By similarity). Required for normal assembly of the mitotic spindle ([PubMed:24255106](http://www.ncbi.nlm.nih.gov/pubmed/24255106/?report=Abstract&tool=FlyBase)). May be involved in both centrosome-dependent and centrosome-independent spindle assembly program |
| DS10_00010177 | DopEcR | Molecular function: G-protein coupled amine receptor activity. Biological process: response to starvation; response to sucrose stimulus; response to ecdysone; cellular response to starvation; cellular response to sucrose stimulus. | 2.11888372746236e-05 | Dmel\DopEcR Dopamine/Ecdysteroid receptor ([DopEcR](http://flybase.org/search/DopEcR)) encodes a GPCR that shows ligand-biased activation. It can be activated by dopamine to increase cyclic AMP levels and by the insect steroid ecdysone to activate the MAPKinase pathway. It is widely expressed in the nervous system and can modulate a wide variety of complex behaviors including male courtship, locomotion, the response to stressful social interactions and the regulation of appetite. |
| DS10_00004539 | LysS | Molecular function: lysozyme activity. Biological process: antimicrobial humoral response | 2.19778740710231e-05 | Dmel\LysS Predicted to have lysozyme activity. Predicted to be involved in defense response to Gram-negative bacterium. Predicted to localize to extracellular space. |
| DS10_00001216 | N/A | Molecular function: acetyl-CoA C-acyltransferase activity. Biological process: metabolic process | 2.28665749838483e-05 | Dmel\CG17597 Predicted to have transferase activity, transferring acyl groups other than amino-acyl groups. Predicted to be involved in positive regulation of intracellular cholesterol transport. Predicted to localize to peroxisome |
| DS10_00006495 | N/A | Molecular function is unknown. Biological process is unknown. | 2.3779286127679e-05 | Dmel\CG15717 Predicted to have oxidoreductase activity. Predicted to be involved in oxidation-reduction process. Is expressed in several structures, including embryonic/larval fat body; embryonic/larval muscle system; extended germ band embryo; foregut primordium; and yolk nucleus. The Other oxidoreductases, is a collection of oxidoreductases that do not fit into any of the other major oxidoreductases. Molecular function: [oxidoreductase activity](http://flybase.org/reports/GO:0016491) Biological process: [oxidation-reduction process](http://flybase.org/reports/GO:0055114) |
| DS10_00004194 | N/A | Molecular function: zinc ion binding; nucleic acid binding. Biological process is unknown. | 2.42785686396505e-05 | Dmel\CG10147 Predicted to have DNA-binding transcription factor activity and transcription regulatory region sequence-specific DNA binding activity. Predicted to be involved in regulation of transcription, DNA-templated. Predicted to localize to nucleus. |
| DS10_00005322 | N/A | Molecular function is unknown. Biological process is unknown. Top nucleotide BLAST: PREDICTED: Drosophila suzukii uncharacterized LOC108016663 (LOC108016663), transcript variant X4, mRNA top protein BLAST: PREDICTED: uncharacterized protein LOC108016663 [Drosophila suzukii] | 2.44666868959998e-05 | Dmel\CG32816 |
| DS10_00003577 | N/A | Molecular function: cysteine-type endopeptidase activity; cysteine-type peptidase activity. Biological process: multicellular organism reproduction. | 2.46896425703968e-05 | Dmel\CG4847 Predicted to have cysteine-type endopeptidase activity. Involved in multicellular organism reproduction. Localizes to extracellular space. |
| DS10_00009090  Potential DOUBLE GENE see DS10_00009088 | N/A | Molecular function: tRNA binding; translation initiation factor activity; ribosome binding. Biological process: regulation of translation; ribosome assembly. | 2.56147272661621e-05 | Dmel\CG7414 Predicted to have ribosome binding activity; tRNA binding activity; and translation initiation factor activity. Predicted to be involved in regulation of translation. Predicted to localize to cytosolic small ribosomal subunit. |
| DS10_00011402 | N/A | Molecular function: catalytic activity. Biological process is unknown. | 2.55881493862124e-05 | Dmel\CG9801 Predicted to have catalytic activity. |
| DS10_00003838 | Hsp67Bb | Molecular function is unknown. Biological process: response to heat. | 2.57630785996642e-05 | Dmel\CG4456 **Hsp22 - Hsp-G3**  There are seven closely related heat-shock genes at 67B (Ayme and Tissieres, 1985; Pauli, Arrigo, Vasquez, Tonka, and Tissieres, 1989, Genome 31: 671-76). In addition to the four small heat-shock genes previously identified (Hsp22, Hsp23, Hsp26, and Hsp27), three more genes (Hsp-G1, Hsp-G2, and Hsp-G3, formerly called Gene1, Gene2, and Gene3) have been found clustered within 15 kb of DNA at the same 67B cytological location. All seven genes are heat-shock inducible in almost all cells at the stages tested (Ayme and Tissieres, 1985). The genes are also transcribed during certain developmental stages in the absence of heat shock (Sirotkin and Davidson, 1982, Dev. Biol. 89: 196-210). Pauli et al (1989) report that the maximum accumulation of developmental rRNA in a majority of these small heat-shock genes occurs in the white pupae stage; in Hsp-G2, however, a small transcipt is found in embryos, first and second instar larvae, and young pupae; and a larger transcript in the pupal and adult stages of males (Pauli and Tonka, 1987, J. Mol. Biol. 198: 235-40; Pauli, Tonka, and Ayme-Southgate, 1988, J. Mol. Biol. 200: 47-53). In absence of stress, the expression of Hsp26 has been observed in spermatocytes, nurse cells, epithelium, imaginal discs, proventriculus, and neurocytes (Glaser, Wolfner, and Lis, 1986, EMBO 5: 747-54). Transcripts of Hsp26 and Hsp27 accumulate in adult ovaries, apparently originating in nurse cells (Zimmerman, Petri, and Meselson, 1983, Cell 32: 1161-70). |
| DS10_00006184 | n/a | Molecular function: metalloendopeptidase activity. Biological process: proteolysis. | 2.61691021110257e-05 | Dmel\goe gone early ([goe](http://flybase.org/search/goe)) encodes a non-peptidase homolog of Neprilysin (M13) family metalloendopeptidases. It is expressed in various EGF (epidermal growth factor) ligand-producing cells and can attenuate EGFR signaling. The roles of the procut of [goe](http://flybase.org/search/goe) include regulation of primordial germ cell differentiation and germline stem cell formation. |
| DS10_00012380 | fax | Molecular function is unknown. Biological process: axonogenesis; neurogenesis. | 2.63840290536785e-05 | Fax failed axon connections Involved in axonogenesis and female germ-line stem cell population maintenance. Localizes to cytoplasm and plasma membrane. Is expressed in several structures, including adult brain; adult heart; egg chamber; germ layer; and presumptive embryonic/larval peripheral nervous system. |
| DS10_00001607 | N/A | Molecular function is unknown. Biological process: neurogenesis. | 2.68480127461169e-05 | Dmel\CG4364 Predicted to be involved in maturation of LSU-rRNA from tricistronic rRNA transcript (SSU-rRNA, 5.8S rRNA, LSU-rRNA). UniProt: Required for maturation of ribosomal RNAs and formation of the large ribosomal subunit. |
| DS10_00000987 | N/A | Molecular function is unknown. Biological process is unknown. Top nucleotide BLAST: PREDICTED: Drosophila suzukii extensin (LOC108004988), mRNA top protein BLAST: PREDICTED: extensin [Drosophila suzukii] | 2.76615624127241e-05 | Dmel\CG10680 |
| DS10_00005153 | deltaCOP | Molecular function is unknown. Biological process: larval salivary gland morphogenesis; regulation of tube diameter, open tracheal system; regulation of lipid storage; protein secretion; phagocytosis, engulfment. | 2.81981650864544e-05 | Dmel\δCOP Coat Protein (coatomer) δ ([δCOP](http://flybase.org/search/%CE%B4COP)) mutants show narrow tracheal tubes due to defective luminal extracellular matrix secretion. |
| DS10_00010788 | unc79 | Molecular function is unknown. Biological process: response to anesthetic | 2.8155006563372e-05 | Dmel\unc79 uncoordinated 79 ([unc79](http://flybase.org/search/unc79)) encodes a protein involved in circadian locomotor rhythms. |
| DS10_00012353 | side | Molecular function is unknown. Biological process: motor neuron axon guidance. | 2.81770033318566e-05 | Dmel\side sidestep ([side](http://flybase.org/search/side)) encodes a transmembrane protein of the immunoglobulin superfamily that is expressed in a defined spatiotemporal pattern in glial cells, sensory neurons and muscles. It functions as an attractive guidance cue to guide motor axons to their target regions. |
| DS10_00000864 | N/A | Molecular function is unknown. Biological process is unknown. Top Nucleotide BLAST: PREDICTED: Drosophila suzukii insulin-like growth factor-binding protein complex acid labile subunit (LOC108021482), mRNA top protein BLAST: PREDICTED: insulin-like growth factor-binding protein complex acid labile subunit [Drosophila suzukii] | 2.84819946065835e-05 | Dmel\CG18095 Predicted to localize to extracellular space. |
| DS10_00010736 | N/A | Molecular function is unknown. Biological process is unknown Top nucleotide BLAST:PREDICTED: Drosophila suzukii uncharacterized LOC108016410 (LOC108016410), transcript variant X2, mRNA top protein BLAST: PREDICTED: uncharacterized protein LOC108016410 isoform X1 [Drosophila suzukii] | 2.85510005541483e-05 | Dmel\CG9616 |
| DS10_00002798 | N/A | Molecular function is unknown. Biological process is unknown. Top nucleotide BLAST: PREDICTED: Drosophila suzukii TATA-binding protein-associated factor 2N (LOC108008642), transcript variant X1, mRNA top protein BLAST: PREDICTED: TATA-binding protein-associated factor 2N isoform X1 [Drosophila suzukii] | 2.88250087143556e-05 | Dmel\CG12911 Is expressed in several structures, including gnathal segment; labral sensory complex; presumptive embryonic/larval system; sensory nervous system primordium; and ventral sensory complex primordium. |
| DS10_00007396 | N/A | Molecular function is unknown. Biological process is unknown. Top nucleotide BLAST: PREDICTED: Drosophila suzukii alpha-tocopherol transfer protein-like (LOC108010830), transcript variant X1, mRNA top protein BLAST: PREDICTED: alpha-tocopherol transfer protein-like isoform X1 [Drosophila suzukii] | 2.9017687113435e-05 | Dmel\CG11550 Predicted to have phosphatidylinositol bisphosphate binding activity. |
| DS10_00007687 | Vha68-1 | Molecular function: ATP binding; proton-transporting ATPase activity, rotational mechanism. Biological process: cellular response to hypoxia. | 2.90713260611402e-05 | Dmel\Vha68-1 Vacuolar H^+^ ATPase 68kD subunit 1 Predicted to have proton-transporting ATPase activity, rotational mechanism. Involved in cellular response to hypoxia. |
| DS10_00003759 | Zasp66 | Molecular function: protein phosphatase 1 binding. Biological process: mesoderm development. | 2.91704496969583e-05 | Dmel\Zasp66 Z band alternatively spliced PDZ-motif protein 66 ([Zasp66](http://flybase.org/search/Zasp66)) encodes a scaffold protein that binds the product of [Actn](http://flybase.org/search/Actn) and localizes to Z-discs in muscle cells. It plays a role in muscle development, especially in myofibril assembly. |
| DS10_00003248 | Cbp53E | Molecular function: calcium ion binding. Biological process: cellular calcium ion homeostasis. | 2.95322542999227e-05 | Dmel\Cbp53E Calbindin 53E ([Cbp53E](http://flybase.org/search/Cbp53E)) encodes a 6 EF-hand domain-containing protein that regulates neuronal development. |
| DS10_00012654 | Obp83cd | Obp83cd Molecular function: odorant binding. Biological process: sensory perception of chemical stimulus | 2.9609723996339e-05 | Dmel\Obp83cd Predicted to have odorant binding activity. Predicted to be involved in sensory perception of smell. Predicted to localize to extracellular region |
| DS10_00000107 | ft | Molecular function: cell adhesion molecule binding. Biological process: biological regulation; single-organism developmental process; establishment of planar polarity; cellular component organization or biogenesis; sensory organ development; post-embryonic organ morphogenesis; negative regulation of biological process; cell proliferation; macromolecule modification; growth; regulation of growth. | 3.01284010226142e-05 | Dmel\ft fat ([ft](http://flybase.org/search/ft)) is a tumor suppressor gene that encodes a large cadherin family transmembrane protein. It functions in the Hippo signaling pathway (as a receptor) and the Dachsous-Fat planar cell polarity pathway (as a transmembrane component). |
| DS10_00008284 | CREG | Molecular function: transcription factor binding. Biological process: negative regulation of transcription from RNA polymerase II promoter. | 3.01523865576702e-05 | Dmel\CREG Cellular Repressor of E1A-stimulated Genes ([CREG](http://flybase.org/search/CREG)) encodes an essential protein located in lysosomes and related subcellular compartments. |
| DS10_00008232 | N/A | Molecular function: oxidoreductase activity, acting on CH-OH group of donors. Biological process: metabolic process. | 3.11338748760506e-05 | Dmel\CG7322 Predicted to have L-xylulose reductase (NADP+) activity; carbonyl reductase (NADPH) activity; and oxidoreductase activity, acting on NAD(P)H, quinone or similar compound as acceptor. Predicted to be involved in glucose metabolic process and xylulose metabolic process |
| DS10_00005300 | N/A | Molecular function: arginase activity. Biological process is unknown. | 3.13136835943345e-05 | Dmel\CG4293 Predicted to be involved in endoplasmic reticulum to Golgi vesicle-mediated transport and retrograde vesicle-mediated transport, Golgi to endoplasmic reticulum. Predicted to localize to COPII-coated ER to Golgi transport vesicle; endoplasmic reticulum; and integral component of organelle membrane. |
| DS10_00012087 | N/A | Molecular function: inositol monophosphate 1-phosphatase activity. Biological process: dephosphorylation | 3.16447612525039e-05 | Dmel\CG17027 Inositol-phosphate phosphatase Predicted to have inositol monophosphate 1-phosphatase activity. Predicted to be involved in inositol metabolic process; inositol phosphate dephosphorylation; and signal transduction. |
| DS10_00007869 | RpL28 | Molecular function: structural constituent of ribosome. Biological process: mitotic spindle elongation; mitotic spindle organization; centrosome duplication; neurogenesis. | 3.21446798454741e-05 | Dmel\RpL28 Predicted to be a structural constituent of ribosome. Predicted to be involved in cytoplasmic translation. Predicted to localize to cytosolic large ribosomal subunit. |
| DS10_00004427 | N/A | Molecular function: 3-oxoacid CoA-transferase activity. Biological process: ketone body catabolic process. | 3.25994262684772e-05 | Dmel\SCOT Succinyl-CoA:3-ketoacid CoA transferase Exhibits 3-oxoacid CoA-transferase activity. Predicted to be involved in ketone body catabolic process. Predicted to localize to mitochondrial matrix. |
| DS10_00008662 | N/A | Molecular function: cysteine-type peptidase activity. Biological process: proteolysis. | 3.30620667207482e-05 | Dmel\CG11459 Predicted to have cysteine-type endopeptidase activity. Predicted to be involved in proteolysis involved in cellular protein catabolic process. Predicted to localize to extracellular space and lysosome. |
| DS10_00008402 | CtsB1 | Molecular function: cysteine-type endopeptidase activity. Biological process: autophagic cell death; salivary gland cell autophagic cell death. | 3.33627429043638e-05 | CtsB1 Cathepsin B1 ([CtsB1](http://flybase.org/search/CtsB1)) encodes a papain-like cysteine proteinase involved in lysosomal protein degradation. |
| DS10_00006845 | Sptr | Molecular function: sepiapterin reductase activity. Biological process: tetrahydrobiopterin biosynthetic process; oxidation-reduction process | 3.36324283391018e-05 | DMel/sptr Sepiapterin reductase ([Sptr](http://flybase.org/search/Sptr)) encodes a sepiapterin reductase involved in the biosynthesis of tetrahydrobiopterin |
| DS10_00005733 | Idgf5 | Molecular function: imaginal disc growth factor receptor binding. Biological process: imaginal disc development. | 3.45030786396496e-05 | Dmel\Idgf5 Exhibits imaginal disc growth factor receptor binding activity. Involved in chitin-based cuticle development; imaginal disc development; and wound healing The Imaginal Disc Growth Factors (IDGF) belong to the 18-glycosylhydrolase family. IDGFs possess a single glyco18 domain which is catalytically inactive and may act as a chitin binding module. |
| DS10_00001874 | No results | No results Top nucleotide BLAST: PREDICTED: Drosophila suzukii annexin A7 (LOC108021554), mRNA top protien BLAST: PREDICTED: annexin A7 [Drosophila suzukii] | 3.47881387889787e-05 | No results |
| DS10_00006179 | eIF-2alpha | Molecular function: translation initiation factor activity. Biological process: mitotic spindle elongation; mitotic spindle organization. | 3.4974754889891e-05 | Dmel\eIF2α eukaryotic translation initiation factor 2 subunit alpha ([eIF2α](http://flybase.org/search/eIF2%CE%B1)) encodes one of three subunits of the eIF-2 complex required for the initiation of translation. [eIF2α](http://flybase.org/search/eIF2%CE%B1) is classified as a 'Minute' gene as heterozygous mutants exhibit a slower developmental rate and small adult bristles |
| DS10_00000571 | N/A | Molecular function is unknown. Biological process is unknown. | 3.59334019696273e-05 | Dmel\CG9643 Predicted to have protein-lysine N-methyltransferase activity. Predicted to be involved in peptidyl-lysine dimethylation and peptidyl-lysine monomethylation. Predicted to localize to cytoplasm The 'Other protein-lysine N-methyltransferases' is a collection of protein-lysine N-methyltransferases that do not fit into any of the other major groups. |
| DS10_00002296 | No results | No results [tiny gene] Top nucleotide result: Drosophila melanogaster strain rover (forR) chromosome 2R top protein BLAST: hypothetical protein [Asgard group archaeon] | 3.58838134893249e-05 | No results |
| DS10_00001275 | No results | No results Top nucleotide BLAST: PREDICTED: Drosophila suzukii uncharacterized LOC108021579 (LOC108021579), transcript variant X3, mRNA top protein BLAST: PREDICTED: uncharacterized protein LOC108021579 [Drosophila suzukii] | 3.64315848623318e-05 | No results |
| DS10_00002465 | Drip | Molecular function: water channel activity. Biological process: renal system process; transmembrane transport; regulation of oviposition; water homeostasis; water transport. | 3.64482719381282e-05 | Dmel\Drip Drip ([Drip](http://flybase.org/search/Drip)) encodes a protein that belongs to the MIP/aquaporin family involved in the transport of water across the cell membrane. In addition to its cellular roles, it has been implicated in regulating aspects of reproduction and aging |
| DS10_00010409 | N/A | Molecular function: sodium:iodide symporter activity. Biological process: transmembrane transport. | 3.70927334313771e-05 | Dmel\CG6723 Predicted to have transmembrane transporter activity. Predicted to be involved in transmembrane transport. Predicted to localize to membrane Solute carrier family 5 (SLC5) members are sodium-dependent glucose transporters. |
| DS10_00000145 | N/A | Molecular function: serine-type endopeptidase inhibitor activity. Biological process is unknown. | 3.7430096601419e-05 | Dmel\CG31777 Predicted to have serine-type endopeptidase inhibitor activity. |
| DS10_00005329 | N/A | Molecular function: 3-hydroxybutyrate dehydrogenase activity. Biological process: inter-male aggressive behavior; olfactory behavior; mushroom body development. | 3.78446349263536e-05 | Dmel\CG13377 Is expressed in embryonic/larval muscle system; gut section; and ventral nerve cord. The phenotypes of these alleles manifest in: mushroom body alpha-lobe; adult mushroom body alpha'-lobe. The phenotypic classes of alleles include: smell perception defective; stress response defective; behavior defective; viable |
| DS10_00004959 | N/A | Molecular function: cysteine-type endopeptidase inhibitor activity. Biological process is unknown. | 3.87462232029892e-05 | Dmel\CG15369 Predicted to have cysteine-type endopeptidase inhibitor activity. |
| DS10_00012333 | GNBP2 | Molecular function: pattern recognition receptor activity. Biological process: defense response. | 3.87242691359114e-05 | Dmel\GNBP2 Exhibits (1->3)-beta-D-glucan binding activity and lipopolysaccharide binding activity. Predicted to be involved in pattern recognition receptor signaling pathway. Predicted to localize to extracellular region. Is expressed in embryonic/larval fat body and organism. Gram-negative bacteria binding protein 2 Involved in the recognition of invading micro-organisms. Binds specifically to beta-1,3-glucan and activates the phenoloxidase cascade (By similarity). |
| DS10_00001496 | No results | No results Top Nucleotide BLAST: PREDICTED: Drosophila suzukii uncharacterized LOC108015863 (LOC108015863), mRNA top protein BLAST: PREDICTED: uncharacterized protein LOC108015863 [Drosophila suzukii] | 3.90799022044341e-05 | No results |
| DS10_00009005 | No results | No results Top nucleotide result: PREDICTED: Drosophila suzukii serine/threonine-protein kinase S6KL (LOC108004842), transcript variant X4, mRNA Top protein BLAST: PREDICTED: serine/threonine-protein kinase S6KL isoform X2 [Drosophila suzukii] | 3.9117921524617e-05 | No results |
| DS10_00005189 | N/A | Molecular function: 2 iron, 2 sulfur cluster binding; oxidoreductase activity; flavin adenine dinucleotide binding. Biological process: oxidation-reduction process; cell redox homeostasis. | 4.07442906746034e-05 | Dmel\CG4199 Predicted to have 2 iron, 2 sulfur cluster binding activity; flavin adenine dinucleotide binding activity; and oxidoreductase activity. Predicted to be involved in oxidation-reduction process. |
| DS10_00009121 | N/A | Molecular function: signal transducer activity. Biological process: signal transduction; filopodium assembly. | 4.1298281626968e-05 | Dmel\IRSp53 Insulin receptor substrate 53 kDa ([IRSp53](http://flybase.org/search/IRSp53)) encodes a protein required for filopodia formation during the adhesion and fusion of myoblasts |
| DS10_00001674 | N/A | Molecular function is unknown. Biological process is unknown. | 4.27777454208644e-05 | Dmel\CG7099 Predicted to be involved in 5S class rRNA transcription by RNA polymerase III and transcription initiation from RNA polymerase III promoter. Predicted to localize to transcription factor TFIIIC complex. |
| DS10_00004905 | N/A | Molecular function is unknown. Biological process is unknown. Top nucleotide BLAST: PREDICTED: Drosophila suzukii transcription initiation factor TFIID subunit 11 (LOC108015040), mRNA top protein BLAST: PREDICTED: transcription initiation factor TFIID subunit 11 [Drosophila suzukii] | 4.28101224440254e-05 | Dmel\CG1468 |
| DS10_00001280 | N/A | Molecular function: pristanoyl-CoA oxidase activity. Biological process: fatty acid beta-oxidation | 4.31039614853036e-05 | Dmel\CG17544 Predicted to have fatty acid binding activity; flavin adenine dinucleotide binding activity; and pristanoyl-CoA oxidase activity. Predicted to be involved in fatty acid beta-oxidation using acyl-CoA oxidase and lipid homeostasis. Localizes to peroxisome. |
| DS10_00010575 | N/A | Molecular function: 2-hydroxyacylsphingosine 1-beta-galactosyltransferase activity. Biological process: metabolic process. | 4.30920402606339e-05 | Dmel\Ugt303B2 UDP-glycosyltransferase family 303 member B2 Predicted to have UDP-glycosyltransferase activity. Predicted to localize to intracellular membrane-bounded organelle. |
| DS10_00011368 | Glt | Molecular function: calcium ion binding. Biological process is unknown | 4.31734123764413e-05 | Dmel\Glt Glutactin ([Glt](http://flybase.org/search/Glt)) encodes a secreted protein that belongs to the family of cholinesterase/carboxylesterase-like proteins but lacks the catalytically active residues. It forms part of the basement membrane and regulates axonal outgrowth. |
| DS10_00005214 | trol | Molecular function is unknown. Biological process: response to anesthetic; maintenance of epithelial cell apical/basal polarity. | 4.35932924763148e-05 | Trol terribly reduced optic lobes ([trol](http://flybase.org/search/trol)) encodes the extracellular matrix component Perlecan, a secreted heparan sulfate proteoglycan. It regulates cell-signaling by multiple growth factors including those in the hedgehog, wingless/WNT, FGF, TGFbeta, EGF, and VEGF families, thus playing a role in many developmental patterning decisions. [Date last reviewed: 2019-06-27] |
| DS10_00012499 | N/A | Molecular function is unknown. Biological process is unknown. Top nucleotide BLAST: PREDICTED: Drosophila suzukii uncharacterized LOC108019379 (LOC108019379), mRNA Top protein BLAST: PREDICTED: uncharacterized protein LOC108019379 [Drosophila suzukii] | 4.45919511286621e-05 | Dmel\CG13071 |
| DS10_00011344 | No results | No results Top nucelotide BLAST: PREDICTED: Drosophila suzukii glucosylceramidase (LOC108007856), transcript variant X1, mRNA top protein BLAST: PREDICTED: glucosylceramidase isoform X1 [Drosophila suzukii] | 4.54639116816996e-05 | No results |
| DS10_00010519 | N/A | Molecular function is unknown. Biological process: lateral inhibition. | 4.70955108834338e-05 | Dmel\CG7668 |
| DS10_00006594 | N/A | Molecular function: methylenetetrahydrofolate dehydrogenase [NAD(P)+] activity. Biological process is unknown. | 4.81221445039102e-05 | Dmel\CG4716 Methylenetetrahydrofolate dehydrogenase (NAD(+)) ([1.5.1.15](https://enzyme.expasy.org/EC/1.5.1.15))  Methylenetetrahydrofolate dehydrogenase (NADP(+)) |
| DS10_00002860 | N/A | Molecular function is unknown. Biological process is unknown. | 4.95106227169317e-05 | Dmel\LTV1 LTV1 ribosome biogenesis factor ([LTV1](http://flybase.org/search/LTV1)) encodes a protein involved in pre-rRNA processing that is required for the proper biogenesis of the 40S ribosome. [LTV1](http://flybase.org/search/LTV1) product is a direct transcriptional target of [Myc](http://flybase.org/search/Myc), thus it is critical for cell growth and survival. |
| DS10_00002240 | N/A | Molecular function: serine-type peptidase activity. Biological process: proteolysis | 5.09440069662916e-05 | Dmel\CG30091 Predicted to have serine-type endopeptidase activity. Predicted to be involved in proteolysis. |
| DS10_00002986 | N/A | Molecular function: 5-formyltetrahydrofolate cyclo-ligase activity. Biological process: folic acid-containing compound biosynthetic process | 5.09304775282284e-05 | Dmel\Mthfs Predicted to have 5-formyltetrahydrofolate cyclo-ligase activity. Predicted to be involved in folic acid-containing compound biosynthetic process and tetrahydrofolate interconversion. |
| DS10_00003152 | N/A | Molecular function: hydrogen-exporting ATPase activity, phosphorylative mechanism. Biological process: proton transport. | 5.13509604504689e-05 | Dmel\CG10731 Predicted to be involved in proton transmembrane transport. Predicted to localize to mitochondrial proton-transporting ATP synthase complex, coupling factor F(o). Is expressed in organism. |
| DS10_00005737 | IM1 | Molecular function is unknown. Biological process: defense response | 5.13443189145809e-05 | Dmel\BomS1 Bomanin Short 1 ([BomS1](http://flybase.org/search/BomS1)) encodes a protein that belongs to a class of small secreted peptides released in the hemolymph upon Toll pathway activation during microbial infection. |
| DS10_00010897 | N/A | Molecular function: oxidoreductase activity. Biological process: metabolic process. | 5.1855551453161e-05 | Dmel\CG31548 17-beta-estradiol 17-dehydrogenase ([1.1.1.62](https://enzyme.expasy.org/EC/1.1.1.62))  Testosterone 17-beta-dehydrogenase (NADP(+) Predicted to have estradiol 17-beta-dehydrogenase activity and testosterone 17-beta-dehydrogenase (NADP+) activity. Is expressed in adult head and adult heart. |
| DS10_00000374 | RpS13 | Molecular function: structural constituent of ribosome. Biological process: mitotic spindle elongation; mitotic spindle organization. | 5.30610366368207e-05 | RpS13 Ribosomal protein S13 ([RpS13](http://flybase.org/search/RpS13)) encodes a structural constituent of ribosomes. |
| DS10_00006638 | Nacalpha | Molecular function: protein binding. Biological process: regulation of pole plasm oskar mRNA localization; oogenesis; neurogenesis. | 5.32083812968509e-05 | Dmel\Nacα Predicted to localize to nascent polypeptide-associated complex. Is expressed in several structures, including embryonic/larval midgut; embryonic/larval muscle system; extended germ band embryo; presumptive embryonic/larval system; and visceral muscle primordium. |
| DS10_00002052 | betaTub56D | Molecular function: structural constituent of cytoskeleton; GTP binding. Biological process: centrosome duplication. | 5.36125174369544e-05 | Dmel\βTub56D A structural constituent of cytoskeleton. Predicted to be involved in microtubule cytoskeleton organization and mitotic cell cycle. Localizes to microtubule Tubulins are cytoskeletal proteins. α- and β-tubulin heterodimers polymerise to form microtubules, the roles of which include mechanical strength, intracellular trafficking and chromosome segregation. |
| DS10_00002505 | conv | Molecular function is unknown. Biological process: motor neuron axon guidance; regulation of tube size, open tracheal system; open tracheal system development; regulation of tube length, open tracheal system. | 5.38381862369653e-05 | Dmel\conv convoluted ([conv](http://flybase.org/search/conv)) encodes a leucine-rich repeat protein involved in axon guidance and the development of the tracheal system. |
| DS10_00001526 | Myo28B1 | Molecular function: ATPase activity, coupled; motor activity; actin binding. Biological process is unknown. | 5.4507350008442e-05 | Dmel\Myo28B1 Myosin 28B1 ([Myo28B1](http://flybase.org/search/Myo28B1)) encodes a high duty ratio motor protein belonging to the MyTH4-FERM subgroup of actin-based molecular motors. |
| DS10_00006102 | N/A | Molecular function: sodium channel activity. Biological process: sodium ion transport. | 5.55422633843466e-05 | Dmel\ppk8 Predicted to have sodium channel activity. Predicted to be involved in sodium ion transport. Predicted to localize to integral component of membrane. Is expressed in adult fat body [PICKPOCKET GENES](http://flybase.org/reports/FBgg0000201.html) -  The degenerin (DEG)/epithial Na^+^ channel (ENaC) gene family, known as the pickpocket genes in Drosophila, encodes subunits of non-voltage gated, amiloride-sensitive cation channels. Channels may be formed by homo- or heteromeric arrangements of subunits. Each subunit has two transmembrane domains and a large cysteine-rich extracellular loop domain. They are functionally diverse, with roles in fluid and salt absorbance, mechanosensation and chemosensation. |
| DS10_00001156 | N/A | Molecular function is unknown. Biological process: synaptic target recognition. | 5.56445502871276e-05 | Dmel\CG5758 [CG5758](http://flybase.org/search/CG5758) encodes a predicted secreted protein with beta-Ig-H3/fasciclin domains. [CG5758](http://flybase.org/search/CG5758) overexpression causes lethality and wing adhesion defects. |
| DS10_00012335 | Cyp12c1 | Molecular function: electron carrier activity. Biological process: oxidation-reduction process. | 5.57258179198536e-05 | Dmel\Cyp12c1 Predicted to have heme binding activity; iron ion binding activity; and oxidoreductase activity, acting on paired donors, with incorporation or reduction of molecular oxygen. Predicted to be involved in oxidation-reduction process |
| DS10_00003353 | wun2 | Molecular function: lipid transporter activity; lipid phosphatase activity. Biological process: pole cell development; pole cell migration; germ cell migration; germ cell development; germ cell repulsion. | 5.69798537432019e-05 | Dmel\wun2 wunen-2 ([wun2](http://flybase.org/search/wun2)) encodes a lipid phosphate phosphatase, an enzyme with an active site predicted to face extracellularly. It is required for germ cell migration and survival, acting partially redundantly with its paralog [wun](http://flybase.org/search/wun). |
| DS10_00003969 | N/A | Molecular function is unknown. Biological process: GPI anchor biosynthetic process. | 5.73894668694777e-05 | Dmel\CG6409 Is expressed in adult hemolymph; embryonic anal pad; embryonic large intestine; and embryonic ventral epidermis. |
| DS10_00002298 | eIF-5A | olecular function: translation regulator activity. Biological process: wing disc development; autophagic cell death; multicellular organism reproduction; salivary gland cell autophagic cell death. | 5.76029053862158e-05 | Multiple results, see Tuesday, June 16, 2020 Dmel\eEF5 Exhibits translation elongation factor activity. Involved in multicellular organism reproduction and positive regulation of translational elongation. Predicted to localize to cytoplasm. Is expressed in adult head; adult heart; and organism. Orthologous to several human genes including EIF5A (eukaryotic translation initiation factor 5A) and EIF5A2 (eukaryotic translation initiation factor 5A2). |
| DS10_00010592 | Cyp6d4 | Molecular function: electron carrier activity. Biological process: chaeta development; wing disc development | 5.80472179277824e-05 | Dmel\Cyp6d4 Predicted to have heme binding activity; iron ion binding activity; and oxidoreductase activity, acting on paired donors, with incorporation or reduction of molecular oxygen. Predicted to be involved in oxidation-reduction process |
| DS10_00002643 | Cyp12d1-d | Molecular function: electron carrier activity. Biological process: response to insecticide; response to DDT. | 5.82277974452415e-05 | TWO GENES: Dmel\Cyp12d1-d Cyp12d1-d ([Cyp12d1-d](http://flybase.org/search/Cyp12d1-d)) encodes a protein involved in the response to the insecticide dichlorodiphenyltrichloroethane (DDT). AND Dmel\Cyp12d1-p Cyp12d1-p ([Cyp12d1-p](http://flybase.org/search/Cyp12d1-p)) encodes a protein involved in the response to the insecticide dichlorodiphenyltrichloroethane (DDT) |
| DS10_00010694 | Cha | Molecular function: choline O-acetyltransferase activity. Biological process: neuromuscular synaptic transmission | 5.94819426854662e-05 | Dmel\ChAT Choline acetyltransferase ([ChAT](http://flybase.org/search/ChAT)) encodes a protein that catalyzes the biosynthesis of the neurotransmitter acetylcholine. It is considered to be a specific marker for cholinergic neurons. |
| DS10_00007464 | N/A | Molecular function: protein binding. Biological process: IMP biosynthetic process. | 5.96389611585121e-05 | Dmel\AMPdeam Predicted to have AMP deaminase activity. Predicted to be involved in AMP metabolic process and IMP biosynthetic process. Localizes to cytosol. Human ortholog(s) of this gene implicated in hereditary spastic paraplegia 63 and pontocerebellar hypoplasia type 9. Is expressed in several structures, including dorsal closure embryo; embryonic epidermis; embryonic/larval garland cell; gut section; and hindgut proper primordium. Orthologous to several human genes including AMPD2 (adenosine monophosphate deaminase 2). |
| DS10_00000381 | mtsh | Molecular function is unknown. Biological process: astral spindle assembly involved in male meiosis; male meiosis cytokinesis; mitochondrion organization; regulation of mitochondrial fusion; spermatogenesis. | 6.00569368459223e-05 | Dmel\mtsh mitoshell ([mtsh](http://flybase.org/search/mtsh)) encodes a protein with a bromodomain-related region. It is required during spermatogenesis for proper temporal coordination of meiotic divisions with the morphogenetic events that usually follow meiosis. [Date last reviewed: 2019-05-1 |
| DS10_00007835 | Lmpt | Molecular function: sequence-specific DNA binding transcription factor activity. Biological process is unknown | 6.04497948080569e-05 | Dmel\Lmpt Limpet Predicted to have zinc ion binding activity. Involved in defense response to fungus. Localizes to I band. Is expressed in several structures, including embryonic/larval muscle system; epithelium; presumptive embryonic/larval system; spermatozoon; and visceral muscle primordium. |
| DS10_00004385 | N/A | Molecular function: purine-nucleoside phosphorylase activity. Biological process: nucleoside metabolic process. | 6.06892678675446e-05 | Dmel\CG16758 Predicted to have purine-nucleoside phosphorylase activity. Predicted to be involved in nucleoside metabolic process. Predicted to localize to cytoplasm |
| DS10_00003283 | N/A | Molecular function is unknown. Biological process is unknown. | 6.07946882402146e-05 | Dmel\CG15617 Predicted to have actin binding activity and muscle alpha-actinin binding activity. Predicted to be involved in actin cytoskeleton organization and muscle structure development. Predicted to localize to Z disc; actin cytoskeleton; and adherens junction. Is expressed in adult head. |
| DS10_00005879 | shi | Molecular function: actin binding; microtubule binding. Biological process: biological regulation; localization; cellular component organization or biogenesis; learning or memory; single-organism developmental process; synaptic transmission; open tracheal system development; associative learning; cellular component movement; regulation of actin filament-based process; cognition. | 6.31010155562931e-05 | Dmel\shi shibire Exhibits actin binding activity and microtubule binding activity. Involved in several processes, including endocytosis; germ cell development; and learning or memory. Localizes to several cellular components, including mitotic spindle; sperm individualization complex; and synapse. Used to study collagen disease. The shibire locus is characterized by its temperature-sensitive alleles, which are reversibly paralyzed by exposure to 29, but are essentially normal at 22 (Grigliatti et al.). Exposure of developing animals to the restrictive temperature for pulses of one to several hours leads to a plethora of developmental defects, which are specific for the stage treated (Poodry, Hall, and Suzuki, 1973, Dev. Biol. 66: 442-56) (see following table). Short exposures to restrictive temperatures at the time of delamination of the neuroblasts from the neurogenic ectoderm leads to excess neurogenesis at the expense of epidermogenesis, as seen in the neurogenic mutants (Poodry, 1990, Dev. Biol., in press) |
| DS10_00012420 | Pep | Molecular function: single-stranded RNA binding; single-stranded DNA binding; DNA binding. Biological process: regulation of mRNA splicing, via spliceosome. | 6.35638915571438e-05 | Dmel\PepProtein on ecdysone puffs [Ecdysone Puffs: part of a healthy, balanced breakfast] Exhibits single-stranded DNA binding activity and single-stranded RNA binding activity. Involved in regulation of mRNA splicing, via spliceosome. Localizes to catalytic step 2 spliceosome and precatalytic spliceosome. Colocalizes with polytene chromosome puff. Is expressed in adult head and adult heart. |
| DS10_00010368 | SIFR | Molecular function: neuropeptide receptor activity. Biological process: determination of adult lifespan. | 6.45097327025398e-05 | Dmel\SIFaR SIFamide receptor ([SIFaR](http://flybase.org/search/SIFaR)) encodes a G-protein coupled peptide receptor involved in both the neuropeptide and the G-protein coupled receptor signaling pathways. It is required for sensory perception of pain, determination of lifespan and sexual behavior. |
| DS10_00012768 | NUCB1 | Molecular function: DNA binding; calcium ion binding. Biological process: multicellular organism reproduction. | 6.45834311978663e-05 | Dmel\NUCB1 NUCB1 ([NUCB1](http://flybase.org/search/NUCB1)) encodes a protein involved in the defense response to Gram-negative bacteria. |
| DS10_00013208 | N/A | Molecular function: aminopeptidase activity. Biological process: proteolysis | 6.49306768673548e-05 | Dmel\CG5849 Predicted to have metalloaminopeptidase activity; peptide binding activity; and zinc ion binding activity. Predicted to be involved in peptide catabolic process and proteolysis. Predicted to localize to cytoplasm |
| DS10_00007343 | disco | Molecular function: DNA binding. Biological process: brain development; eclosion rhythm; locomotor rhythm; positive regulation of transcription from RNA polymerase II promoter. | 6.51892749041358e-05 | Dmel\disco disconnected ([disco](http://flybase.org/search/disco)) encodes a C2H2 zinc finger transcription factor involved in development of ventral appendages (e.g. legs and some mouthparts) during embryogenesis and metamorphosis |
| DS10_00002717 | Ipk1 | Molecular function: inositol pentakisphosphate 2-kinase activity. Biological process: inositol phosphate biosynthetic process. | 6.60510586130242e-05 | Dmel\Ipk1 Exhibits inositol pentakisphosphate 2-kinase activity. Involved in inositol phosphate biosynthetic process and inositol phosphorylation. Predicted to localize to nucleus. I |
| DS10_00003311 | Pmm45A | Molecular function: phosphomannomutase activity. Biological process is unknown. | 6.66676567043112e-05 | Dmel\Pgm2a Predicted to have intramolecular transferase activity, phosphotransferases and magnesium ion binding activity. Predicted to be involved in carbohydrate metabolic process. |
| DS10_00006886 | N/A | Molecular function: serine-type peptidase activity. Biological process is unknown | 6.70801600222673e-05 | Dmel\CG2145 Exhibits endoribonuclease activity. Predicted to be involved in RNA phosphodiester bond hydrolysis, endonucleolytic. Is expressed in adult head. |
| DS10_00007512 | stnB | Molecular function: protein binding. Biological process: synaptic transmission; regulation of synaptic vesicle endocytosis; synaptic vesicle transport; synaptic vesicle endocytosis. | 6.69084827766323e-05 | Dmel\stnB stoned B Exhibits scaffold protein binding activity. Involved in several processes, including regulation of synaptic vesicle endocytosis; synaptic vesicle endocytosis; and synaptic vesicle transport. Localizes to endocytic vesicle; presynaptic active zone; and synaptic vesicle The stoned proteins, stoned A and B, are encoded by a dicistronic locus in insecta. They are structurally different, but both contain motifs that are found in adaptor proteins. The stoned proteins are believed to act in synaptic vesicle recycling pathways. |
| DS10_00008829 | N/A | Molecular function: protein binding. Biological process: multicellular organism reproduction. | 6.70528384550723e-05 | Dmel\CG18135 Exhibits myosin binding activity. Involved in multicellular organism reproduction. Localizes to extracellular space. Is expressed in several structures, including amnioserosa; gut section; head; and yolk nucleus |
| DS10_00010220 | N/A | Molecular function: translation initiation factor activity. Biological process: autophagic cell death; salivary gland cell autophagic cell death. | 6.70988483838036e-05 | Dmel\eIF3f1 Predicted to contribute to translation initiation factor activity. Involved in positive regulation of Notch signaling pathway. Predicted to localize to eukaryotic translation initiation factor 3 complex, eIF3m. |
| DS10_00004444 | N/A | Molecular function: structural constituent of chitin-based cuticle. Biological process is unknown. | 6.75267317375269e-05 | Dmel\Cpr62Bc Predicted to be a structural constituent of chitin-based larval cuticle. Predicted to be involved in chitin-based cuticle development. |
| DS10_00008661 | N/A | Molecular function: cysteine-type peptidase activity. Biological process: proteolysis. | 6.77813488247841e-05 | Dmel\CG11459 Predicted to have cysteine-type endopeptidase activity. Predicted to be involved in proteolysis involved in cellular protein catabolic process. Predicted to localize to extracellular space and lysosome |
| DS10_00001872 | N/A | Molecular function is unknown. Biological process is unknown. | 6.82132009592562e-05 | Dmel\CG33129 Involved in response to hyperoxia. Localizes to endomembrane system. Is expressed in several structures, including adult heart; embryonic/larval salivary gland; extended germ band embryo; presumptive embryonic/larval system; and ventral ectoderm. |
| DS10_00003766 | SrpRbeta | Molecular function: GTPase activity; signal recognition particle binding. Biological process: chitin-based larval cuticle pattern formation; larval chitin-based cuticle development; neurogenesis. | 7.00105395344601e-05 | Dmel\SrpRβ Signal recognition particle receptor β Signal recognition particle receptor β ([SrpRβ](http://flybase.org/search/SrpR%CE%B2)) encodes a protein that contributes to cuticle secretion by epidermal cells. |
| DS10_00000767 | N/A | Molecular function: oxidoreductase activity, acting on the CH-OH group of donors, NAD or NADP as acceptor. Biological process: metabolic process. | 7.18847618625849e-05 | Dmel\CG31937 Is expressed in several structures, including anterior endoderm anlage; embryonic/larval midgut; embryonic/larval midgut primordium; embryonic/larval visceral muscle; and germ layer. |
| DS10_00000306 | N/A | Molecular function: glycerate kinase activity. Biological process: protein phosphorylation. | 7.27260257803294e-05 | Dmel\CG9886 Predicted to have glycerate kinase activity. Predicted to be involved in protein phosphorylation. Predicted to localize to cytoplasm |
| DS10_00001816 | RluA-1 | Molecular function: diaminohydroxyphosphoribosylaminopyrimidine deaminase activity. Biological process: riboflavin biosynthetic process. | 7.32617542279809e-05 | Dmel\RluA-1 RluA pseudouridine synthase 1 ([RluA-1](http://flybase.org/search/RluA-1)) encodes a pseudouridine synthase, an enzyme catalyzing the formation of the most abundant nucleoside modification of RNA, pseudouridine |
| DS10_00005866 | eIF5 | Molecular function: translation initiation factor activity. Biological process: neurogenesis. | 7.32678379237806e-05 | Dmel\eIF5 eukaryotic translation initiation factor 5 Predicted to have GDP-dissociation inhibitor activity; eukaryotic initiation factor eIF2 binding activity; and translation initiation factor activity. Predicted to be involved in formation of cytoplasmic translation initiation complex and formation of translation preinitiation complex |
| DS10_00007897 | N/A | Molecular function is unknown. Biological process is unknown. Top protein BLAST: PREDICTED: prominin-like protein [Drosophila suzukii] top nucleotide BLAST: PREDICTED: Drosophila suzukii prominin-like protein (LOC108007101), mRNA | 7.35500067087339e-05 | Multiple Results, See Wednesday, June 17, 2020. Dmel\CG45066, Dmel\CG45067 Predicted to localize to integral component of membrane |
| DS10_00006566 | N/A | Molecular function is unknown. Biological process is unknown. Top nucleotide BLAST: PREDICTED: Drosophila suzukii uncharacterized LOC108019737 (LOC108019737), mRNA top protein BLAST: PREDICTED: uncharacterized protein LOC108019737 [Drosophila suzukii] | 7.42248014270216e-05 | Dmel\CG13332 |
| DS10_00011589 | lt | Molecular function: zinc ion binding. Biological process: Notch receptor processing; dsRNA transport; endocytosis; determination of adult lifespan; ommochrome biosynthetic process. | 7.5372053729319e-05 | Dmel\lt light ([lt](http://flybase.org/search/lt)) encodes a cellular-protein trafficking protein that forms part of the homotypic fusion and vacuolar protein sorting (HOPS) and class C core vacuole/endosome tethering (CORVET) complexes. It is required for fusion of autophagosomes with lysosomes, endocytic down-regulation of Notch signaling, and eye pigment biogenesis. |
| DS10_00012651 | Cad87A | Molecular function: calcium ion binding. Biological process: calcium-dependent cell-cell adhesion | 7.61989961363978e-05 | Dmel\Cad87A Cadherin 87A Predicted to have calcium ion binding activity. Predicted to be involved in calcium-dependent cell-cell adhesion via plasma membrane cell adhesion molecules and cell-cell adhesion mediated by cadherin. Predicted to localize to integral component of plasma membrane. |
| DS10_00002672 | N/A | Molecular function is unknown. Biological process is unknown. Top nucleotide BLAST: PREDICTED: Drosophila suzukii uncharacterized LOC108009459 (LOC108009459), mRNA top protein BLAST:PREDICTED: uncharacterized protein LOC108009459 [Drosophila suzukii] | 7.66375601595579e-05 | Dmel\CG13220 Is expressed in several structures, including adult head; adult heart; embryonic/larval muscle system; extended germ band embryo; and gut section. |
| DS10_00000508 | N/A | Molecular function: actin-dependent ATPase activity; protein homodimerization activity; structural constituent of muscle. Biological process: single-organism developmental process; cellular component organization or biogenesis; muscle cell differentiation; actin filament-based process; localization; cellular component movement; organelle organization; biological regulation; flight; system process. | 7.71587698964585e-05 | Dmel\Mhc Myosin heavy chain ([Mhc](http://flybase.org/search/Mhc)) encodes the motor protein that provides the force for muscle contraction through its ATP-dependent interaction with actin filaments. It functions with essential and regulatory light chains |
| DS10_00003901 | N/A | Molecular function: sphingomyelin phosphodiesterase activity. Biological process is unknown. | 7.82350166317058e-05 | Dmel\CG32052 Predicted to have phosphoric diester hydrolase activity. Predicted to localize to extracellular space. Is expressed in adult head and embryonic head sensory system. |
| DS10_00006630 | N/A | Molecular function: carnitine transmembrane transporter activity. Biological process: transmembrane transport. | 7.81234254245939e-05 | Dmel\Balat Beta-alanine transporter ([Balat](http://flybase.org/search/Balat)) is predicted to encode a carnitine transporter, which catalyzes the movement of acyl-carnitine across the inner mitochondrial membrane. It is essential for metabolism of long-chain fatty acids. |
| DS10_00008255 | N/A | Molecular function: serine-type endopeptidase activity. Biological process: proteolysis. | 8.15413303706872e-05 | Dmel\ CG5246 Predicted to have serine-type endopeptidase activity. Predicted to be involved in proteolysis. |
| DS10_00002817 | N/A | Molecular function is unknown. Biological process: peroxisome fission. | 8.33464017777621e-05 | Dmel\CG33474 Predicted to be involved in peroxisome fission. Predicted to localize to integral component of peroxisomal membrane. |
| DS10_00002908 | Adam | Molecular function: translation initiation factor activity. Biological process: neurogenesis | 8.32418803518579e-05 | Dmel\eIF3j Exhibits translation initiation factor activity. Involved in regulation of translational initiation. Localizes to eukaryotic translation initiation factor 3 complex. Is expressed in several structures, including ectoderm anlage; extended germ band embryo; germ layer; gut section; and stage 5 embryo. |
| DS10_00004426 | N/A | Molecular function is unknown. Biological process is unknown.Top nucletoide BLAST: PREDICTED: Drosophila suzukii uncharacterized LOC108012876 (LOC108012876), transcript variant X2, mRNAtop Protein BLAST: PREDICTED: uncharacterized protein LOC108012876 isoform X1 [Drosophila suzukii] | 8.40732502307065e-05 | Dmel\CG1927 Is expressed in embryonic/larval midgut and organism |
| DS10_00013514 | N/A | Molecular function: oxidoreductase activity, acting on the CH-OH group of donors, NAD or NADP as acceptor. Biological process: metabolic process. | 8.45559165330937e-05 | Dmel\CG6012 Is expressed in adult head. Steroid dehydrogenases are NAD or NADP dependent oxidoreductases with steroid as a substrate. Most members of the family contain the tyrosine and lysine of the catalytic triad in a YxxxK sequence |
| DS10_00002345 | N/A | Molecular function is unknown. Biological process: lateral inhibition. | 8.71764208572109e-05 | Dmel\side-VIII Is expressed in RP neuron; adult head; and pCC neuron. SIDE is a closely related family encoding immunoglobulin superfamily proteins. They can interact with BEAT family members. Interactions between [side](http://flybase.org/search/side) and [beat-Ia](http://flybase.org/search/beat-Ia) have been shown to play a role in the control of motor axon guidance. |
| DS10_00003042 | N/A | Molecular function: catalytic activity. Biological process: metabolic process. | 8.71351556349893e-05 | Dmel\CG3663 Predicted to have catalytic activity. Predicted to localize to cytoplasm. Is expressed in several structures, including anterior endoderm; anterior endoderm anlage; embryonic/larval midgut; embryonic/larval muscle system; and extended germ band embryo. |
| DS10_00001736 | nimC1 | Molecular function: Wnt-protein binding. Biological process: phagocytosis. | 8.78735957973993e-05 | Dmel\NimC1 Nimrod C1 Involved in defense response to bacterium and phagocytosis. Localizes to plasma membrane. Is expressed in adult head; cortical zone of lymph gland primary lobe; embryonic/larval lymph gland; hemocyte; and plasmatocyte. The Nimrod family of genes is characterised by the presence of NIM repeats, closely related to the EGF-repeat. They encode proteins which are secreted or integral to the plasma membrane. The Nimrod genes have been linked to the innate immune response and some members of this family have been shown to act as receptors for bacterial or apoptic cell phagocytosis |
| DS10_00009030 | Atpalpha | Molecular function: sodium:potassium-exchanging ATPase activity; cation transmembrane transporter activity. Biological process: biological regulation; response to stimulus; locomotory behavior; cellular component organization or biogenesis; single-organism developmental process; regulation of anatomical structure size; system process; determination of adult lifespan; synaptic transmission; response to mechanical stimulus; open tracheal system development; response to abiotic stimulus; localization; neuromuscular process; response to temperature stimulus. | 8.85368844165426e-05 | Dmel\Atpα Na pump α subunit ([Atpα](http://flybase.org/search/Atp%CE%B1)) encodes an integral membrane cation antiporter protein that utilizes ATP to shuttle Na[+] and K[+] across the plasma membrane to maintain ion homeostasis. |
| DS10_00005942 | N/A | Molecular function: low-density lipoprotein receptor activity. Biological process: proteolysis. | 8.93425524559937e-05 | Dmel\CG1632 Predicted to be involved in proteolysis. Is expressed in spermatozoon. Serine protease homologs of the S1A family are similar in amino acid sequence to S1A serine proteases but are enzymatically inactive due to the lack of catalytic residue(s). Their functions are generally poorly understood, though at least some of them have known regulatory functions. |
| DS10_00012092 | elgi | Molecular function: protein binding. Biological process: regulation of meiotic cell cycle. | 8.97205688529278e-05 | Elgi early girl ([elgi](http://flybase.org/search/elgi)) encodes a myosin binding protein involved in the regulation of meiosis and Fat signaling pathway. |
| DS10_00010100 | Aats-tyr | Molecular function: tyrosine-tRNA ligase activity. Biological process: tyrosyl-tRNA aminoacylation. | 9.28302806164537e-05 | Dmel\TyrRS Exhibits tyrosine-tRNA ligase activity. Predicted to be involved in tyrosyl-tRNA aminoacylation. Predicted to localize to cytoplasm. Used to study Charcot-Marie-Tooth disease. |
| DS10_00000858 | N/a | Molecular function is unknown. Biological process is unknown. | 9.34235999560662e-05 | Dmel\CG10947 Predicted to have calmodulin-lysine N-methyltransferase activity. Predicted to be involved in regulation of translation. Predicted to localize to cytoplasm and nucleus |
| DS10_00010895 | N/A | Molecular function: oxidoreductase activity. Biological process: metabolic process. | 9.36636432830131e-05 | Dmel\CG31549 Predicted to have estradiol 17-beta-dehydrogenase activity and testosterone 17-beta-dehydrogenase (NADP+) activity. Is expressed in adult head and organism. |
| DS10_00009421 | N/A | Molecular function: metalloendopeptidase activity. Biological process: proteolysis. | 9.49615016464926e-05 | Dmel\Nepl12 Predicted to have metalloendopeptidase activity. Predicted to be involved in proteolysis |
| DS10_00007210 | N/A | Molecular function: lysozyme activity. Biological process is unknown. | 9.68176953728873e-05 | Dmel\CG14823 Predicted to have lysozyme activity. Lysozymes belong to the glycoside hydrolase family 22. Lysozymes damage bacterial cell walls by hydrolyzing the β-(1,4)-glycosidic bond between N-acetyl-muraminic acid and N-acetyl-D-glucosamine of peptidoglycan, causing cell lysis. |
| DS10_00002237 | MESK2 | Molecular function is unknown. Biological process: positive regulation of Ras protein signal transduction. | 9.72376083147255e-05 | Dmel\MESK2 Misexpression suppressor of KSR 2 Predicted to be involved in signal transduction. Predicted to localize to cytoplasm. Human ortholog(s) of this gene implicated in Charcot-Marie-Tooth disease type 4D. Is expressed in adult head; wing hinge primordium; and wing pouch. |
| DS10_00011543 | N/A | Molecular function: alditol:NADP+ 1-oxidoreductase activity. Biological process: mushroom body development. | 9.7950816011045e-05 | Dmel\CG6083 Predicted to have alditol:NADP+ 1-oxidoreductase activity. Involved in mushroom body development. Predicted to localize to cytosol. Human ortholog(s) of this gene implicated in 46,XY sex reversal 8; B-lymphoblastic leukemia/lymphoma; T-cell acute lymphoblastic leukemia; and leukemia. |
| DS10_00007403 | N/A | Molecular function: unfolded protein binding. Biological process: tubulin complex assembly. | 9.85242770762415e-05 | Dmel\CG1890 Predicted to have tubulin binding activity. Predicted to be involved in protein folding and tubulin complex assembly. Predicted to localize to cytosol and microtubule cytoskeleton. |
| DS10_00004080 | mop | Molecular function: protein tyrosine phosphatase activity. Biological process: positive regulation of epidermal growth factor receptor signaling pathway; antimicrobial peptide production; spindle assembly; regulation of growth; positive regulation of Toll signaling pathway. | 9.86856572654089e-05 | Dmel\mop myopic ([mop](http://flybase.org/search/mop)) encodes an endocytic protein that is required for normal Egfr signaling and for the progression of the product of [Egfr](http://flybase.org/search/Egfr) through the endocytic pathway. It also controls the endosomal localization and signaling activity of the product of [yki](http://flybase.org/search/yki). |
| DS10_00008911 | N/A | Molecular function: protein binding. Biological process: multicellular organism reproduction. | 9.91651842535299e-05 | Dmel\CG18135 Exhibits myosin binding activity. Involved in multicellular organism reproduction. Localizes to extracellular space. Is expressed in several structures, including amnioserosa; gut section; head; and yolk nucleus. |
| DS10_00004138 | N/A | Molecular function is unknown. Biological process is unknown. Top nucleotide BLAST:PREDICTED: Drosophila suzukii uncharacterized LOC108012986 (LOC108012986), mRNA top Protein BLAST: PREDICTED: uncharacterized protein LOC108012986 [Drosophila suzukii] | 0.00010019863492946 | Dmel\CG9760 |
| DS10_00008488 | N/A | Molecular function: electron carrier activity. Biological process: oxidative phosphorylation. | 0.000101444647320566 | Dmel\CG7834 Predicted to have electron transfer activity. Predicted to be involved in oxidative phosphorylation. Localizes to mitochondrion Electron transferring flavoproteins (ETFs) are heterodimeric FAD-containing proteins that function primarily as soluble electron carriers between various flavoprotein dehydrogenases to membrane-bound ETF ubiquinone oxidoreductase, which reduces ubiquinone to ubiquinol which enters the respiratory chain |
| DS10_00012265 | desat1 | Molecular function: stearoyl-CoA 9-desaturase activity. Biological process: cuticle hydrocarbon biosynthetic process; regulation of lipid metabolic process; pheromone biosynthetic process; male mating behavior; mating behavior, sex discrimination; regulation of autophagy. | 0.000102130314871233 | Dmel\Desat1 Desaturase 1 ([Desat1](http://flybase.org/search/Desat1)) encodes a transmembrane fatty acid desaturase that utilizes myristate and stearate ( C16:0 and C18:0 ) to synthesize myristoleic and oleic acids ( C16:1 and C18:1 ). It is involved in the synthesis of unsaturated fatty acids. |
| DS10_00005735 | N/A | Molecular function is unknown. Biological process is unknown. | 0.000104508858721658 | Dmel\BomT2 Bomanin Tailed 2 Bomanins are a family of small, secreted immune-induced peptides that are induced by Toll signalling. These proteins may confer resistance to bacterial infection. Secreted immune-induced peptide induced by Toll signaling ([PubMed:25915418](http://www.ncbi.nlm.nih.gov/pubmed/25915418/?report=Abstract&tool=FlyBase), [PubMed:29920489](http://www.ncbi.nlm.nih.gov/pubmed/29920489/?report=Abstract&tool=FlyBase)). Has a role in resistance to bacterial and fungal infections ([PubMed:25915418](http://www.ncbi.nlm.nih.gov/pubmed/25915418/?report=Abstract&tool=FlyBase), [PubMed:29920489](http://www.ncbi.nlm.nih.gov/pubmed/29920489/?report=Abstract&tool=FlyBase)). The strength of antimicrobial activity appears to correlate with the overall level of expression ([PubMed:29920489](http://www.ncbi.nlm.nih.gov/pubmed/29920489/?report=Abstract&tool=FlyBase)). |
| DS10_00006171 | Sirup | Molecular function is unknown. Biological process is unknown | 0.000104238770683326 | Dmel\Sirup Starvation-upregulated protein ([Sirup](http://flybase.org/search/Sirup)) encodes a critical assembly factor for Complex II in the electron transport chain of mitochondria. Loss of [Sirup](http://flybase.org/search/Sirup) results in motility defects, neurodegeneration, and oxidative stress. |
| DS10_00011763 | Obp83ef | Molecular function: odorant binding. Biological process: sensory perception of chemical stimulus. | 0.000104431066995731 | Dmel\Obp83ef Predicted to have odorant binding activity. Predicted to be involved in sensory perception of smell. Predicted to localize to extracellular region. |
| DS10_00005691 | scra | Molecular function: actin binding; microtubule binding. Biological process: cellular component organization or biogenesis; cellular process; single-organism developmental process; sensory organ development; system development; cell cycle; multicellular organismal development; organelle organization; cell cycle process; biological regulation. | 0.000106094987920699 | Dmel\scra scraps ([scra](http://flybase.org/search/scra)) encodes a homolog of anilin, a conserved pleckstrin homology domain (PLEKH) containing protein that binds actin, nonmuscle myosin II and microtubules. It stabilizes the contractile ring and is required for completion of cytokinesis |
| DS10_00002525  A lot is known about this gene, and it plays a role in several pathways | phyl | Molecular function: protein binding; protein self-association; protein binding, bridging; binding, bridging. Biological process: sensory organ development; single-organism developmental process; biological regulation; cell fate commitment; neuron differentiation; regulation of cellular process; regulation of signal transduction; system development; formation of organ boundary; compound eye morphogenesis. | 0.000107266938417552 | Dmel\phyl phyllopod Exhibits protein binding activity, bridging involved in substrate recognition for ubiquitination. Involved in several processes, including negative regulation of signal transduction; positive regulation of proteasomal ubiquitin-dependent protein catabolic process; and sensory organ development. |
| DS10_00009481 | N/A | Molecular function is unknown. Biological process: olfactory behavior. | 0.000108620978695117 | Dmel\jus julius seizure ([jus](http://flybase.org/search/jus)) encodes a two-transmembrane domain protein expressed in selected neurons in the optic lobe, subesophageal and thoracic-abdominal ganglia. It is mainly localized to axons and its developmental expression prevents epileptogenesis. Involved in mechanosensory behavior and regulation of membrane potential. Used to study epilepsy. Is expressed in several structures, including adult head; central nervous system; and embryonic/larval nervous system.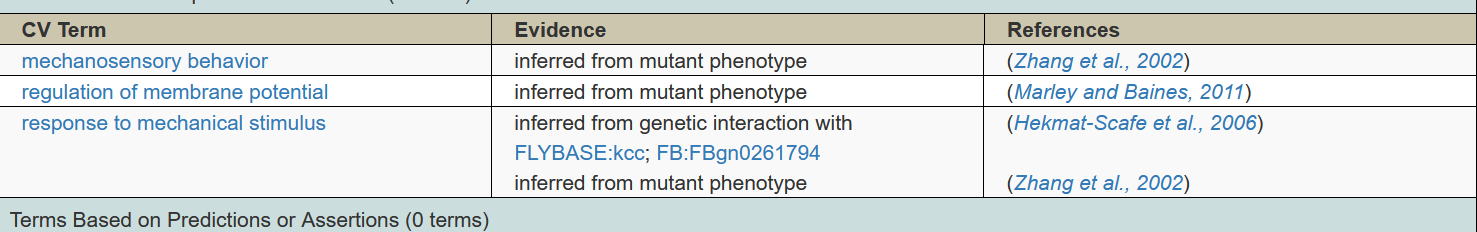 |
| DS10_00008205 | N/A | Molecular function: serine-type endopeptidase activity. Biological process: proteolysis. | 0.000110870892177822 | Dmel\CG6067 Predicted to be involved in proteolysis. Is expressed in adult head. |
| DS10_00007856  This antifungal is downregulated, interestingly | Drsl6 | Molecular function is unknown. Biological process: defense response to fungus. | 0.000111293780966742 | Dmel\Drsl6 Drosomycin-like 6 ([Drsl6](http://flybase.org/search/Drsl6)) encodes a secreted peptide with homology to the antifungal peptide encoded by [Drs](http://flybase.org/search/Drs). |
| DS10_00005581 | Dpit47 | Molecular function is unknown. Biological process is unknown. | 0.000111780017417587 | Dmel\Dpit47 DNA polymerase interacting tpr containing protein of 47kD ([Dpit47](http://flybase.org/search/Dpit47)) encodes a TPR motif containing protein that appears to act as a co-chaperone protein in the Hsp90 pathway. |
| DS10_00001401 | N/A | Molecular function: glutaryl-CoA dehydrogenase activity. Biological process: lysine catabolic process; hydroxylysine catabolic process; tryptophan metabolic process. | 0.000112806975341535 | Dmel\CG9547 Predicted to have glutaryl-CoA dehydrogenase activity. Predicted to be involved in hydroxylysine catabolic process; lysine catabolic process; and tryptophan metabolic process. Predicted to localize to mitochondrial matrix. |
| DS10_00011441 | N/A | Molecular function: alditol:NADP+ 1-oxidoreductase activity. Biological process: mushroom body development | 0.000113894411176824 | Dmel\CG6083 Predicted to have alditol:NADP+ 1-oxidoreductase activity. Involved in mushroom body development. Predicted to localize to cytosol. |
| DS10_00009595 | N/A | Molecular function: glycerol-3-phosphate O-acyltransferase activity. Biological process: plasma membrane organization; phospholipid biosynthetic process. | 0.000116187807858323 | Dmel\mino minotaur ([mino](http://flybase.org/search/mino)) encodes a member of the conserved glycerol-3-phosphate O-acyltransferase family, which converts glycerol-3-phosphate into lysophosphatidic acid. It functions in phosphatidic acid biosynthesis. It is also essential for piRNA biogenesis in gonads, with [mino](http://flybase.org/search/mino) mutants showing transposon de-repression and sterility phenotypes. |
| DS10_00001860 | Dnz1 | Molecular function: protein-cysteine S-palmitoleyltransferase activity. Biological process: lateral inhibition. | 0.000117107895024265 | Dmel\Dnz1 DNZDHHC/NEW1 zinc finger protein 11 Predicted to have protein-cysteine S-palmitoyltransferase activity. Predicted to be involved in peptidyl-L-cysteine S-palmitoylation and protein targeting to membrane. Localizes to endoplasmic reticulum |
| DS10_00012919 | ird5 | Molecular function: protein binding; protein serine/threonine kinase activity. Biological process: peptidyl-serine phosphorylation; positive regulation of antibacterial peptide biosynthetic process; regulation of innate immune response; defense response to virus; protein catabolic process; antibacterial humoral response. | 0.000116993827640339 | Dmel\IKKβ I-kappaB kinase β ([IKKβ](http://flybase.org/search/IKK%CE%B2)) encodes a a component of the IκB Kinase (IKK) complex together with the product of [key](http://flybase.org/search/key). IKK complex participates in the activation of the transcription factor encoded by [Rel](http://flybase.org/search/Rel) in the immune deficiency pathway. This pathway regulates the antibacterial response and other less characterized cellular processes |
| DS10_00004550 | N/A | Molecular function is unknown. Biological process is unknown. Top nucleotide result: PREDICTED: Drosophila suzukii uncharacterized LOC108013571 (LOC108013571), mRNA top protein BLAST: PREDICTED: uncharacterized protein LOC108013571 [Drosophila suzukii] | 0.00011905651352989 | Dmel\CG13912 Is expressed in several structures, including embryonic/larval nervous system; extended germ band embryo; germ layer; presumptive embryonic/larval system; and stage 5 embryo. |
| DS10_00008238 | slgA | Molecular function: proline dehydrogenase activity. Biological process: phototaxis; locomotory behavior; proline catabolic process. | 0.000119726800456512 | Dmel\slgA sluggish A Exhibits proline dehydrogenase activity. Involved in several processes, including aggressive behavior; phototaxis; and proline catabolic process. Localizes to mitochondrion. Used to study schizophrenia. |
| DS10_00003738 | N/A | Molecular function: endopeptidase inhibitor activity. Biological process is unknown. | 0.000122916263172105 | Dmel\CG32354 Predicted to be involved in cell differentiation and multicellular organism development. |
| DS10_00008000 | N/A | Molecular function: metalloendopeptidase activity. Biological process: proteolysis. | 0.00012361121515647 | Dmel\Nepl6 Predicted to have metalloendopeptidase activity. Predicted to be involved in proteolysis. |
| DS10_00000432 | obst-B | Molecular function: chitin binding; structural constituent of peritrophic membrane. Biological process: chitin metabolic process. | 0.000124848785125841 | Dmel\obst-B Predicted to have chitin binding activity. Predicted to be involved in chitin metabolic process. Predicted to localize to chitin-based extracellular matrix. Is expressed in epithelium and foregut. |
| DS10_00009512 | N/A | Molecular function is unknown. Biological process is unknown. Top nucleotide BLAST: PREDICTED: Drosophila suzukii uncharacterized LOC108018518 (LOC108018518), mRNA top protein BLAST: PREDICTED: uncharacterized protein LOC108018518 [Drosophila suzukii] | 0.00012511973955492 | Dmel\CG9837 Is expressed in circular visceral muscle fiber; embryonic/larval fat body; embryonic/larval midgut; and germ layer derivative. |
| DS10_00004631 | N/A | Molecular function: serine-type endopeptidase inhibitor activity. Biological process: regulation of proteolysis. | 0.000125646846589779 | TWO RESULTS: Dmel\CG34454 Predicted to localize to extracellular region and mitochondrion. AND Dmel\CG42846 |
| DS10_00000475 | Idgf2 | Molecular function: imaginal disc growth factor receptor binding. Biological process: imaginal disc development. | 0.000125832343716265 | Dmel\Idgf2 Imaginal disc growth factor 2 ([Idgf2](http://flybase.org/search/Idgf2)) encodes a secreted glycoprotein that is a member of the chitinase-like protein family mainly expressed in fat body and hemocytes. [Idgf2](http://flybase.org/search/Idgf2) product is induced under stress conditions |
| DS10_00009850 | N/A | Molecular function: metalloendopeptidase activity. Biological process: proteolysis. | 0.000127571969487139 | Dmel\Nepl21 Neprilysin-like 21Predicted to have metalloendopeptidase activity. Predicted to be involved in proteolysis. Localizes to extracellular region. Is expressed in adult head. |
| DS10_00012241 | Hexim | Molecular function: 7SK snRNA binding. Biological process: negative regulation of transcription from RNA polymerase II promoter. | 0.000128179157924259 | HeximHexamethylene bisacetamide inducible ([Hexim](http://flybase.org/search/Hexim)) encodes an RNA binding protein. When associated with [snRNA:7SK](http://flybase.org/search/snRNA%3A7SK), it recruits and inhibits the positive transcription elongation factor encoded by [Cdk9](http://flybase.org/search/Cdk9). Regulated release and re-sequestration of the product of [Cdk9](http://flybase.org/search/Cdk9) by the [snRNA:7SK](http://flybase.org/search/snRNA%3A7SK) snRNP plays an essential role in RNA polymerase II elongation control. |
| DS10_00001868 | dpr2 | Molecular function is unknown. Biological process: sensory perception of chemical stimulus. | 0.000128775524220724 | Dmel\dpr2 defective proboscis extension response 2 Involved in synapse organization. Predicted to localize to integral component of plasma membrane and neuron projection membrane. Defective proboscis extension response (Dpr) cell surface proteins are members of the immunoglobulin superfamily. They form a complex interaction network with Dpr-interacting proteins (DIPs), acting as neuronal surface labels in the specificity of synaptic connections between neurons and target cells. (Adapted from [FBrf0230454](http://flybase.org/reports/FBrf0230454.html) and [FBrf0149073](http://flybase.org/reports/FBrf0149073.html)). |
| DS10_00001219 | N/A | Molecular function is unknown. Biological process is unknown. Top nucleotide BLAST: PREDICTED: Drosophila suzukii uncharacterized LOC108005540 (LOC108005540), mRNA top protein BLAST: PREDICTED: uncharacterized protein LOC108005540 [Drosophila suzukii] | 0.000129729376664598 | Dmel\CG17321 Is expressed in atrium; dorsal epidermis primordium; embryonic/larval nervous system; and presumptive embryonic/larval system. |
| DS10_00002630 | N/A | Molecular function: RNA binding; S-adenosylmethionine-dependent methyltransferase activity. Biological process: rRNA processing. | 0.000130196197198201 | Dmel\CG8545 Predicted to have rRNA (cytosine-C5-)-methyltransferase activity. Predicted to be involved in maturation of LSU-rRNA and rRNA base methylation. Predicted to localize to nucleolus. |
| DS10_00000780 | N/A | Molecular function is unknown. Biological process is unknown. Top nucleotide result: PREDICTED: Drosophila suzukii uncharacterized LOC108021204 (LOC108021204), transcript variant X2, mRNA top protein BLAST: PREDICTED: uncharacterized protein LOC108021204 isoform X2 [Drosophila suzukii] | 0.000130553570936699 | Dmel\CG31663 Is expressed in adult head; embryonic Malpighian tubule; embryonic brain; lateral cord neurons; and ventral nerve cord. |
| DS10_00002323 | Alas | Molecular function: 5-aminolevulinate synthase activity. Biological process: chitin-based cuticle development | 0.000135900923708309 | Alas Aminolevulinate synthase ([Alas](http://flybase.org/search/Alas)) encodes a mitochondrial enzyme involved in cuticle barrier formation and thought to have a role in Heme biosynthesis |
| DS10_00011944 | Lsd-1 | Molecular function is unknown. Biological process: triglyceride mobilization; regulation of lipid storage; lipid particle organization; positive regulation of triglyceride catabolic process; neurogenesis. | 0.000136959085290609 | Dmel\Lsd-1 Lipid storage droplet-1 ([Lsd-1](http://flybase.org/search/Lsd-1)) encodes a protein associated with lipid droplets. It protects lipid droplets from lipase mediated remobilization and facilitates lipolysis by serving as an anchoring point for lipases such as the one encoded by [Hsl](http://flybase.org/search/Hsl). It is involved in lipid storage amount regulation and energy homeostasis in concert with the product of [Lsd-2](http://flybase.org/search/Lsd-2). |
| DS10_00011386 | N/A | Molecular function: transferase activity, transferring phosphorus-containing groups. Biological process is unknown. | 0.000138435682939356 | Dmel\CG10514 Is expressed in adult head. |
| DS10_00007488 | N/A | Molecular function: L-tyrosine:2-oxoglutarate aminotransferase activity. Biological process: aromatic amino acid family metabolic process; biosynthetic process. | 0.000138867253597907 | Dmel\CG1461 Predicted to have L-tyrosine:2-oxoglutarate aminotransferase activity and pyridoxal phosphate binding activity. Predicted to be involved in aromatic amino acid family metabolic process and biosynthetic process |
| DS10_00003832 | N/A | Molecular function: pyridoxal kinase activity. Biological process: pyridoxal 5'-phosphate salvage. | 0.000139205992140457 | Dmel\Pdxk Pyridoxal kinase ([Pdxk](http://flybase.org/search/Pdxk)) encodes the enzyme that generates Pyridoxal-5-phosphate, the metabolically active form of vitamin B6. It contributes to chromosome integrity and glucose homeostasis. [ |
| DS10_00010991 | N/A | Molecular function: serine-type endopeptidase activity. Biological process: proteolysis. | 0.000140778458743284 | Dmel\CG11841 Predicted to have serine-type endopeptidase activity. Predicted to be involved in proteolysis. |
| DS10_00012213 | GABA-B-R2 | Molecular function: G-protein coupled GABA receptor activity. Biological process: olfactory behavior; negative regulation of insulin secretion; negative regulation of synaptic vesicle fusion to presynaptic membrane | 0.000141044476272084 | Dmel\GABA-B-R2 Exhibits G protein-coupled GABA receptor activity and protein heterodimerization activity. Involved in several processes, including G protein-coupled receptor signaling pathway; cellular response to mechanical stimulus; and negative regulation of secretion by cell. Localizes to G protein-coupled receptor heterodimeric complex. Metabotropic GABA(B) receptors are Class C GPCRs. They function as obligate heterodimers, binding the inhibitory neurotransmitter GABA (γ-aminobutyric acid) |
| DS10_00007042 | No results | No results Top nucleotide BLAST: PREDICTED: Drosophila suzukii histone acetyltransferase p300-like (LOC108004615), mRNA top protein BLAST: PREDICTED: histone acetyltransferase p300-like [Drosophila suzukii] | 0.000145807769792894 | No results |
| DS10_00011203 | N/A | Molecular function: sodium:iodide symporter activity. Biological process: transmembrane transport. | 0.000148266931828292 | Dmel\CG7720 Predicted to have transmembrane transporter activity. Predicted to be involved in transmembrane transport. Predicted to localize to membrane. Is expressed in adult head; embryonic dorsal epidermis; embryonic large intestine; embryonic ventral epidermis; and large intestine primordium |
| DS10_00002357 | Mgat1 | Molecular function: acetylglucosaminyltransferase activity; alpha-1,3-mannosylglycoprotein 2-beta-N-acetylglucosaminyltransferase activity. Biological process: determination of adult lifespan; protein N-linked glycosylation via asparagine; protein N-linked glycosylation; adult locomotory behavior; encapsulation of foreign target; mushroom body development. | 0.00014971366463009 | Dmel\Mgat1 Mannosyl (α-1,3-)-glycoprotein β-1,2-N-acetylglucosaminyltransferase ([Mgat1](http://flybase.org/search/Mgat1)) encodes an enzyme involved in the synthesis of paucimannose N-glycans. It is required in the CNS, with involvement in locomotion, brain development and lifespan |
