# Supplementary material for: Molecular and behavioral studies reveal differences in olfaction between winter and summer morphs of Drosophila suzukii
Source: PeerJ. 2022 Sep 16;10:e13825. doi: 10.7717/peerj.13825 (PMC9484457; doi:10.7717/peerj.13825)
Supplement: Supplemental Information 3 — Olfactory related genes with ID from gene databases [file peerj-10-13825-s003.docx]

**Olfactory and Metabolism Genes of Interest**

**Total Olfactory Genes List with GO Biological Process Label from *D. suzukii* annotation:**

1. DS10_00007574 Gad1
2. DS10_00006717 Obp44a
3. DS10_00011764 Obp83g
4. DS10_00009743 CheA87a
5. DS10_00009134 Obp56h
6. DS10_00012656 Obp83g
7. DS10_00012654 Obp83cd
8. DS10_00011763 Obp83ef
9. DS10_00009481 N/A [Dmel\jus]
10. DS10_00001868 dpr2
11. DS10_00012213 GABA-B-R2
12. DS10_00005329 N/A [Dmel\CG13377]

**Potential Olfactory Genes that do NOT have a GO Biological Process Label from *D. suzukii* annotation (but do have something listed on FlyBase):**

1. DS10_00012948 Bap55
2. DS10_00002723 Bap55
3. DS10_00010769 Bap55
4. DS10_00006652 nemy
5. DS10_00009056 N/A [Dmel\meigo]
6. DS10_00010808 N/A [Dmel\meigo]

**Olfactory Genes of Interest**

| **Gene Number** | **Sym-bol** | ***D. suzukii* annotation (SpottedWingFlyBase)** | **P-value** | ***D. melanogaster***  **Annotation (FlyBase)** | **Log2FC-value** |
| --- | --- | --- | --- | --- | --- |
| DS10_00007574 | Gad1 | Molecular function: glutamate decarboxylase activity. Biological process: response to mechanical stimulus; olfactory learning; synapse assembly; gamma-aminobutyric acid biosynthetic process; neuromuscular junction development; neurotransmitter receptor metabolic process; glutamate catabolic process; larval locomotory behavior. | 1.17859689029e-06 | Gad1 Glutamic acid decarboxylase 1 ([Gad1](about:blank)) encodes an essential, nervous system-specific glutamic acid decarboxylase, which is the synthetic enzyme for the major inhibitory neurotransmitter gamma-Aminobutyric acid (GABA). It is required for a multitude of physiological functions and adult behaviors dependent on GABA, including sleep, memory, circadian rhythms and egg hatching | 0.85450714344393 |
| DS10_00012948 | Bap55 | Molecular function: transcription coactivator activity. Biological process: positive regulation of gene silencing by miRNA; dendrite morphogenesis; mitosis; cytokinesis; muscle organ development; positive regulation of transcription, DNA-dependent; dendrite guidance | 2.99926009454867e-06 | Dmel\Bap55 Brahma associated protein 55kD (Bap55) encodes a member of two chromatin remodeling complexes. As part of the Brahma complex, it is needed for cell growth and survival in the wing imaginal disc; as a member of the TIP60 complex, it is thought to regulate dendrite wiring specificity in olfactory projection neurons. | 1.42405811701696 |
| DS10_00002723 | Bap55 | Molecular function: transcription coactivator activity. Biological process: positive regulation of gene silencing by miRNA; dendrite morphogenesis; mitosis; cytokinesis; muscle organ development; positive regulation of transcription, DNA-dependent; dendrite guidance. | 7.66316318090589e-06 | Dmel\Bap55 Brahma associated protein 55kD ([Bap55](about:blank)) encodes a member of two chromatin remodeling complexes. As part of the Brahma complex, it is needed for cell growth and survival in the wing imaginal disc; as a member of the TIP60 complex, it is thought to regulate dendrite wiring specificity in olfactory projection neurons.    The Drosophila olfactory system exhibits very precise and stereotyped wiring that is specified predominantly by genetic programming. Dendrites of olfactory projection neurons (PNs) pattern the developing antennal lobe before olfactory receptor neuron axon arrival, indicating an intrinsic wiring mechanism for PN dendrites. These wiring decisions are likely determined through a transcriptional program. We find that loss of Brahma associated protein 55 kD (Bap55) results in a highly specific PN mistargeting phenotype. In Bap55 mutants, PNs that normally target to the DL1 glomerulus mistarget to the DA4l glomerulus with 100% penetrance. Loss of Bap55 also causes derepression of a GAL4 whose expression is normally restricted to a small subset of PNs. Bap55 is a member of both the Brahma (BRM) and the Tat interactive protein 60 kD (TIP60) ATP-dependent chromatin remodeling complexes. The Bap55 mutant phenotype is partially recapitulated by Domino and Enhancer of Polycomb mutants, members of the TIP60 complex. However, distinct phenotypes are seen in Brahma and Snf5-related 1 mutants, members of the BRM complex. The Bap55 mutant phenotype can be rescued by postmitotic expression of Bap55, or its human homologs BAF53a and BAF53b.Our results suggest that Bap55 functions through the TIP60 chromatin remodeling complex to regulate dendrite wiring specificity in PNs. The specificity of the mutant phenotypes suggests a position for the TIP60 complex at the top of a regulatory hierarchy that orchestrates dendrite targeting decisions. | 1.02554365345163 |
| DS10_00010769 | Bap55 | Molecular function: transcription coactivator activity. Biological process: positive regulation of gene silencing by miRNA; dendrite morphogenesis; mitosis; cytokinesis; muscle organ development; positive regulation of transcription, DNA-dependent; dendrite guidance. | 6.24315255258891e-18 | Dmel\Bap55  Brahma associated protein 55kD ([Bap55](about:blank)) encodes a member of two chromatin remodeling complexes. As part of the Brahma complex, it is needed for cell growth and survival in the wing imaginal disc; as a member of the TIP60 complex, it is thought to regulate dendrite wiring specificity in olfactory projection neurons. [Date last reviewed: 2018-10-18] | 2.42262773206725 |
| DS10_00006717  SEE Tuesday, May 19, 2020 for comprehensive gene info | Obp44a | Molecular function: odorant binding. Biological process: sensory perception of chemical stimulus. | 1.69259074585476e-24 | Dmel\Obp44a | 2.54474759137789 |
| DS10_00011764 | Obp83g | Molecular function: odorant binding. Biological process: sensory perception of chemical stimulus. | 7.21435487310578e-10 | Dmel\Obp83g  Odorant-binding protein 83g | 1.55122489902249 |
| DS10_00009743 | CheA87a | Molecular function is unknown. Biological process: sensory perception of chemical stimulus. | 6.67295438624134e-08 | Dmel\CheA87a  Chemosensory protein A 87a | 2.65107791656186 |
| DS10_00009134 | Obp56h | Molecular function: odorant binding. Biological process: sensory perception of smell. | 3.82383318511854e-06 | Dmel\Obp56h Predicted to have odorant binding activity. Involved in mating behavior; mating pheromone secretion; and sensory perception of smell. Predicted to localize to extracellular region. Is expressed in Bolwig organ; embryonic antennal sense organ; and labral sensory complex.Present in the aqueous fluid surrounding olfactory sensory dendrites and are thought to aid in the capture and transport of hydrophobic odorants into and through this fluid (By similarity). May function in both olfactory and gustatory systems. | -0.745662096605553 |
| DS10_00012656 | Obp83g | Molecular function: odorant binding. Biological process: sensory perception of chemical stimulus | 8.15613381973254e-06 | Dmel\Obp83g Predicted to have odorant binding activity. Predicted to be involved in sensory perception of smell. Predicted to localize to extracellular region. Odorant Binding Proteins (OBP) are small (10 to 30 kDa) proteins secreted by auxiliary cells surrounding the olfactory receptor neurons. OBPs are characterized by a specific protein domain that comprises six α-helices joined by three disulfide bonds. Although the full function of the OBPs is not well established, it is believed that they may act as molecular carriers that transport odorants and deliver them to the olfactory receptors located on the sensory neurons. | 1.48218889180986 |
| DS10_00012654 | Obp83cd | Obp83cd Molecular function: odorant binding. Biological process: sensory perception of chemical stimulus | 2.9609723996339e-05 | Dmel\Obp83cd Predicted to have odorant binding activity. Predicted to be involved in sensory perception of smell. Predicted to localize to extracellular region | -1.79399460157585 |
| DS10_00011763 | Obp83ef | Molecular function: odorant binding. Biological process: sensory perception of chemical stimulus. | 0.000104431066995731 | Dmel\Obp83ef Predicted to have odorant binding activity. Predicted to be involved in sensory perception of smell. Predicted to localize to extracellular region. | -1.57371127932424 |
| DS10_00009481 | N/A | Molecular function is unknown. Biological process: olfactory behavior. | 0.000108620978695117 | Dmel\jus julius seizure ([jus](about:blank)) encodes a two-transmembrane domain protein expressed in selected neurons in the optic lobe, subesophageal and thoracic-abdominal ganglia. It is mainly localized to axons and its developmental expression prevents epileptogenesis. Involved in mechanosensory behavior and regulation of membrane potential. Used to study epilepsy. Is expressed in several structures, including adult head; central nervous system; and embryonic/larval nervous system.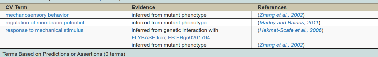 | 0.871823219300531 |
| DS10_00001868 | dpr2 | Molecular function is unknown. Biological process: sensory perception of chemical stimulus. | 0.000128775524220724 | Dmel\dpr2 defective proboscis extension response 2 Involved in synapse organization. Predicted to localize to integral component of plasma membrane and neuron projection membrane. Defective proboscis extension response (Dpr) cell surface proteins are members of the immunoglobulin superfamily. They form a complex interaction network with Dpr-interacting proteins (DIPs), acting as neuronal surface labels in the specificity of synaptic connections between neurons and target cells. (Adapted from [FBrf0230454](about:blank) and [FBrf0149073](about:blank)). | 0.920900602167635 |
| DS10_00012213 | GABA-B-R2 | Molecular function: G-protein coupled GABA receptor activity. Biological process: olfactory behavior; negative regulation of insulin secretion; negative regulation of synaptic vesicle fusion to presynaptic membrane | 0.000141044476272084 | Dmel\GABA-B-R2 Exhibits G protein-coupled GABA receptor activity and protein heterodimerization activity. Involved in several processes, including G protein-coupled receptor signaling pathway; cellular response to mechanical stimulus; and negative regulation of secretion by cell. Localizes to G protein-coupled receptor heterodimeric complex. Metabotropic GABA(B) receptors are Class C GPCRs. They function as obligate heterodimers, binding the inhibitory neurotransmitter GABA (γ-aminobutyric acid) | 0.988527984794906 |
| DS10_00005329 | N/A | Molecular function: 3-hydroxybutyrate dehydrogenase activity. Biological process: inter-male aggressive behavior; olfactory behavior; mushroom body development. | 3.78446349263536e-05 | Dmel\CG13377 Is expressed in embryonic/larval muscle system; gut section; and ventral nerve cord. The phenotypes of these alleles manifest in: mushroom body alpha-lobe; adult mushroom body alpha'-lobe. The phenotypic classes of alleles include: smell perception defective; stress response defective; behavior defective; viable | 0.782736343647319 |
| DS10_00006652 | nemy | Molecular function: carbon-monoxide oxygenase activity. Biological process: locomotory behavior; memory; imaginal disc-derived wing morphogenesis. | 1.47883374443273e-06 | Dmel\nemy no extended memory (nemy) likely encodes a human cytochrome b561 homolog and is involved in middle-term olfactory associative memory and courtship conditioning memory. | 0.907951847617436 |
| DS10_00009056 | N/A | Molecular function: UDP-galactose transmembrane transporter activity. Biological process: transmembrane transport. | 5.65440973280237e-07 | Dmel\meigo medial glomeruli (meigo) encodes an evolutionarily conserved, multi-membrane pass protein that is mainly localized at the endoplasmic reticulum. [meigo](about:blank) genetically interacts with [Ephrin](about:blank) in dendrite targeting of olfactory projection neurons | -1.16369114035183 |
| DS10_00010808 | N/A | Molecular function: UDP-galactose transmembrane transporter activity. Biological process: transmembrane transport. | 2.29992748073955e-09 | Dmel\meigo  medial glomeruli (meigo) encodes an evolutionarily conserved, multi-membrane pass protein that is mainly localized at the endoplasmic reticulum. [meigo](about:blank) genetically interacts with [Ephrin](about:blank) in dendrite targeting of olfactory projection neurons. [Date last reviewed: 2019-03-14] | -1.17829460412383 |

**Metabolism Genes of Interest**

| Gene Number | Sym-bol | *D. suzukii* annotation | P-value | *D. melanogaster*  annotation | Log2FC-value |
| --- | --- | --- | --- | --- | --- |
| DS10_00006223 | Ahcy13 | Molecular function: adenosylhomocysteinase activity. Biological process: one-carbon metabolic process. | 4.15368845535494e-16 | Dmel\Ahcy  Adenosylhomocysteinase (Ahcy) encodes S-adenosyl-L-homocysteine hydrolase, the rate-limiting enzyme in methionine metabolism. This tetrameric enzyme catalyzes the reversible hydrolysis of S-Adenosylhomocysteine (SAH) to adenosine and L-homocysteine. The function of [Ahcy](about:blank) product is required to maintain proper concentrations of SAH, which serves as an inhibitor of S-adenosylmethionine-dependent methylation reactions. [Date last reviewed: 2018-09-13] | -1.8503513381349 |
| DS10_00005254 | N/A | Molecular function: UDP-N-acetylmuramate dehydrogenase activity; flavin adenine dinucleotide binding. Biological process: oxidation-reduction process. | 2.13913347487572e-08 | Dmel\D2hgdh  D-2-hydroxyglutaric acid dehydrogenase ([D2hgdh](about:blank)) encodes an enzyme involved in oxidation-reduction metabolism. [Date last reviewed: 2019-09-19] | -1.1715773713588 |
| DS10_00004961 | su(r) | Molecular function: dihydropyrimidine dehydrogenase (NADP+) activity. Biological process: 'de novo' pyrimidine nucleobase biosynthetic process. | 1.00250748826756e-07 | Dmel\su(r)  suppressor of rudimentary (su(r)) encodes a dihydropyrimidine dehydrogenase, the first step of pyrimidine catabolism. [su(r)](about:blank) depletion does not produce visible phenotypes, but synergistic effects are exhibited by combining mutants with genes affecting pyrimidine biosynthesis and beta-alanine metabolism. [Date last reviewed: 2019-09-26] | -1.00050190150556 |
| DS10_00001045 | Mtp | Molecular function: phosphatidylcholine transporter activity. Biological process: synaptic target recognition; lipoprotein metabolic process. | 6.76571074678629e-06 | Dmel\Mtp Microsomal triacylglycerol transfer protein (Mtp) encodes a phosphatidylcholine transporter involved in lipoprotein metabolism, tracheal system lumen formation and neuron development. | -1.44929264155421 |
| DS10_00011805 | Indy | Molecular function: citrate transmembrane transporter activity; succinate transmembrane transporter activity. Biological process: regulation of sequestering of triglyceride; determination of adult lifespan. | 1.28679519886463e-05 | Dmel\Indy I'm not dead yet ([Indy](about:blank)) encodes a plasma membrane transporter of Krebs cycle intermediates (citrate, succinate, fumarate, alpha-ketoglutarate) of the SLC13A family that is important in organismal intermediary metabolism. Mutations in [Indy](about:blank) create a favorable metabolic state, similar to calorie restriction, and extend life span. | -0.622321406154984 |
| DS10_00012811 | N/A | Molecular function: 2 iron, 2 sulfur cluster binding; iron ion binding; electron carrier activity; oxidoreductase activity, acting on CH-OH group of donors; flavin adenine dinucleotide binding. Biological process: oxidation-reduction process. | 1.45566911591617e-05 | Dmel\AOX2  Aldehyde oxidase 2 ([AOX2](about:blank)) encodes a pyridoxal oxidase involved in pyridoxal metabolism | -0.838234916722379 |

**Vitellogenin Genes of Interest**

| DS10_00004890 | Yp1 | Molecular function: structural molecule activity. Biological process: vitellogenesis. | 4.0038961584228e-29 | Dmel\Yp1 Structural gene for the yolk protein YP1 found in recently-emerged female flies. Protein migrates at different rates in SDS-polyacrylamide gels when encoded by the electrophoretic variants Yp1F (fast) and Yp1S (slow), alleles that are female fertile and produce normal amounts of YP1. Yp1ts1, which maps near the Yp1 locus and is believed to be an allele, produces a slow-migrating translation product that is present in reduced amounts in the hemolymph and the ovaries (Bownes and Hodson, 1980); this mutant is female sterile.  Its molecular function is described by: lipase activity; carboxylic ester hydrolase activity. It is involved in the biological process described with: sex differentiation; lipid catabolic process. | -3.882157821 |
| --- | --- | --- | --- | --- | --- |
| DS10_00004891 | Yp2 | Molecular function: structural molecule activity. Biological process: neurogenesis. | 2.13292529170438e-28 | Dmel\Yp2 carboxylic ester hydrolase activity; lipase activity. It is involved in the biological process described with: lipid catabolic process; sex differentiation.  Structural gene for the yolk protein YP2 found in recently-emerged female flies. Protein migrates at different rates in SDS-polyacrylamide gels when encoded by the electrophoretic variants Yp2F (fast) and Yp2S (slow), alleles that are female fertile and produce normal amounts of YP2. A mutant Yp2M (=Yp212-1245) is female fertile but lays fewer eggs than normal (Mohler, Postlethwait, and Shirk) and does not contain yolk protein in the hemolymph or ovaries.  Vitellogenin is the major yolk protein of eggs where it is used as a food source during embryogenesis. | -4.102114874 |
